# Multi-trait polygenic risk scores improve genomic prediction of atrial fibrillation across diverse ancestries

**DOI:** 10.1038/s41467-026-72708-x

**Published:** 2026-05-05

**Authors:** Poeya Haydarlou, Daria R. Kramarenko, Nobuyuki Enzan, Marie Klevjer, Oliver B. Vad, Marre E. Corver, Dominic S. Zimmerman, the BioBank Japan Project, Koichi Matsuda, Takayuki Morisaki, Yukinori Okada, Yoichiro Kamatani, Kaori Muto, Akiko Nagai, Yoji Sagiya, Natsuhiko Kumasaka, Yoichi Furukawa, Yuji Yamanashi, Yoshinori Murakami, Yusuke Nakamura, Wataru Obara, Ken Yamaji, Kazuhisa Takahashi, Satoshi Asai, Yasuo Takahashi, Shinichi Higashiue, Shuzo Kobayashi, Hiroki Yamaguchi, Yasunobu Nagata, Satoshi Wakita, Chikako Nito, Yu-ki Iwasaki, Shigeo Murayama, Kozo Yoshimori, Yoshio Miki, Daisuke Obata, Masahiko Higashiyama, Akihide Masumoto, Yoshinobu Koga, Yukihiro Koretsune, Koichi Matsuda, Søren Z. Diederichsen, Anja Bye, Jesper H. Svendsen, Kaoru Ito, Patrick T. Ellinor, Connie R. Bezzina, Sean J. Jurgens

**Affiliations:** 1 Department of Experimental Cardiology, Amsterdam Cardiovascular Sciences, Cardiomyopathy & Arrhythmia, Amsterdam UMC location University of Amsterdam, Amsterdam, the Netherlands; 2 European Reference Network for rare low prevalence and complex diseases of the heart (ERN GUARD-Heart), Amsterdam, the Netherlands; 3 Cardiovascular Disease Initiative, Broad Institute of MIT and Harvard, Cambridge, MA USA; 4 Laboratory for Cardiovascular Genomics and Informatics, RIKEN Center for Integrative Medical Sciences, Yokohama, Japan; 5 Department of Advanced Biomedical Data Science, Graduate School of Medicine, Chiba University, Chiba, Japan; 6 Department of Circulation and Medical Imaging, Faculty of Medicine and Health Sciences, Norwegian University of Science and Technology (NTNU), Trondheim, Norway; 7 Department of Cardiology, St.Olavs Hospital, Trondheim University Hospital, Trondheim, Norway; 8 Department of Cardiology, The Heart Centre, Copenhagen University Hospital–Rigshospitalet, Copenhagen, Denmark; 9 Department of Biomedical Sciences, University of Copenhagen, Copenhagen, Denmark; 10 Laboratory of Clinical Genome Sequencing, Department of Computational Biology and Medical Sciences, Graduate School of Frontier Sciences, The University of Tokyo, Tokyo, Japan; 11 Department of Clinical Medicine, University of Copenhagen, Copenhagen, Denmark; 12 Cardiovascular Research Center, Massachusetts General Hospital, Boston, MA USA; 13 Department of Clinical Cardiology, Amsterdam UMC location University of Amsterdam, Amsterdam, the Netherlands; 14 Laboratory of Genome Technology, Human Genome Center, Institute of Medical Science, The University of Tokyo, Tokyo, Japan; 15 Laboratory of Clinical Genome Sequencing, Graduate School of Frontier Sciences, The University of Tokyo, Tokyo, Japan; 16 The Institute of Medical Science, The University of Tokyo, Tokyo, Japan; 17 Department of Genome Informatics, Graduate School of Medicine, The University of Tokyo, Tokyo, Japan; 18 Laboratory of Complex Trait Genomics, Graduate School of Frontier Sciences, The University of Tokyo, Tokyo, Japan; 19 Department of Public Policy, Institute of Medical Science, The University of Tokyo, Tokyo, Japan; 20 Division of Digital Genomics, Institute of Medical Science, The University of Tokyo, Tokyo, Japan; 21 Division of Clinical Genome Research, Institute of Medical Science, The University of Tokyo, Tokyo, Japan; 22 Department of Urology, Iwate Medical University, Iwate, Japan; 23 Department of Internal Medicine and Rheumatology, Juntendo University Graduate School of Medicine, Tokyo, Japan; 24 Department of Respiratory Medicine, Juntendo University Graduate School of Medicine, Tokyo, Japan; 25 Division of Pharmacology, Department of Biomedical Science, Nihon University School of Medicine, Tokyo, Japan; 26 Division of Genomic Epidemiology and Clinical Trials, Clinical Trials Research Center, Nihon University School of Medicine, Tokyo, Japan; 27 Tokushukai Group, Tokyo, Japan; 28 Department of Hematology, Nippon Medical School, Tokyo, Japan; 29 Laboratory for Clinical Research, Collaborative Research Center, Nippon Medical School, Tokyo, Japan; 30 Department of Cardiovascular Medicine, Nippon Medical School, Tokyo, Japan; 31 Tokyo Metropolitan Geriatric Hospital and Institute of Gerontology, Tokyo, Japan; 32 Fukujuji Hospital, Japan Anti-Tuberculosis Association, Tokyo, Japan; 33 The Cancer Institute Hospital of the Japanese Foundation for Cancer Research, Tokyo, Japan; 34 Center for Clinical Research and Advanced Medicine, Shiga University of Medical Science, Shiga, Japan; 35 Department of General Thoracic Surgery, Osaka International Cancer Institute, Osaka, Japan; 36 Iizuka Hospital, Fukuoka, Japan; 37 National Hospital Organization Osaka National Hospital, Osaka, Japan

**Keywords:** Cardiovascular genetics, Predictive medicine, Genetics research

## Abstract

Polygenic scores can improve atrial fibrillation risk prediction. However, limited accuracy and cross-ancestry transferability hinder clinical translation. Here, we explore several ensemble approaches to generate ancestry-optimized polygenic scores, with development in diverse participants from the All of Us Research Program, BioBank Japan, and three additional cohorts. Our ancestry-specific multi-trait approach particularly improves prediction in South-Asian (odds-ratio/standard deviation 1.5–1.8; area under curve 0.60-0.64; relative *R²* +71%), Admixed-American (1.5; 0.60; +34%) and African ancestry groups (1.4; 0.57; +56%). Nevertheless, performance remains highest in European and East-Asian ancestries (1.8–2.2; 0.65–0.68), where >50% of SNP-heritability is explained. Improved risk stratification is also observed at the extremes, identifying European and East-Asian ancestry individuals with risk comparable to rare *TTN* variants (e.g., 6–11% with >4-fold odds). Finally, our scores improve incident risk prediction alongside clinical models. Together, we show that our ancestry-tailored multi-trait polygenic scores advance atrial fibrillation risk prediction and stratification, providing an equitable foundation for implementation.

## Abstract (web summary)

Polygenic scores show promise for atrial fibrillation risk prediction but lack accuracy across ancestries. Here, the authors develop ancestry-specific, multi‑trait ensemble polygenic scores that improve risk prediction and stratification in diverse populations, advancing more equitable clinical implementation.

## Introduction

Polygenic scores (PGSs) have gained considerable interest as tools to predict an individual’s genetic propensity to diverse phenotypes and diseases^1–3^. Generated from large genome-wide association studies (GWAS), PGSs aim to condense multiple common risk alleles into a singular quantitative score^4^. As GWAS sample sizes have grown and PGS prediction has improved, PGSs have garnered substantial interest for potential clinical implementation. Indeed, as of late 2025, Mass General Brigham (MGB) hospital has started offering a PGS testing service, allowing for genetic risk estimation across eight common diseases, among which atrial fibrillation (AF)^5,6^.

AF is the most common sustained cardiac arrhythmia, affecting over 30 million people worldwide^7^. AF is associated with over three-fold risk of both ischemic stroke and heart failure (HF)^8,9^, making it a major contributor to global cardiovascular morbidity and mortality. Large GWAS have successfully established hundreds of common risk loci for AF^10^, and have estimated that common genetic variants explain over 22% of variance in AF susceptibility^11^. Subsequently, PGSs have been developed that aim to estimate genetic risk of AF. In a landmark paper, Roselli et al. built an AF PGS from a GWAS of around 180,000 AF cases, which showed notable prediction of incident AF when used in conjunction with age and sex (C-index ≈ 0.8), and arguably serves as the current gold standard^12^. As opposed to clinical models (such as the CHARGE-AF score^13,14^), a PGS may indicate risk long before traditional clinical risk factors emerge, highlighting their potential for early prediction and prevention^15^.

In spite of the current movement towards clinical implementation, however, PGSs face several major barriers that limit their clinical and research utility. For instance, current AF PGSs show only modest predictive accuracy and lack well-validated clinical use-cases, limiting their potential as prediction tools for actionability in the clinic. Perhaps more importantly, current PGSs transfer poorly across ancestries, with reduced accuracy in non-European populations, which may lead to worsening of existing inequities in healthcare. The moderate transferability in part stems from a persistent lack of diversity in GWAS training datasets and a predominant focus on individuals of European descent^1^. This pattern is evident in existing PGSs for AF reported in the PGS catalog, as most are trained on European data only and are rarely validated across diverse ancestries.

Here, taking AF as exemplary trait, we sought to assess and address these challenges through three key strategies: First, we enhanced ancestral diversity of our training data by integrating GWAS summary statistics from the AFGen Consortium^12^ with the Million Veteran Program (MVP)^16^, the latter being distinguished by its broad ancestral representation comprising approximately 29% non-European individuals^17^. Second, we applied SBayesRC, a Bayesian framework that produces dense genome-wide PGSs that leverage functional genomic annotations to enhance prediction accuracy, particularly across ancestries^18^. Third, we investigated three complementary strategies for polygenic score integration: A multi-ancestry approach combining PGSs derived from different ancestral backgrounds; a multi-methods approach, combining PGS derived from different statistical architectures; and finally, a multi-trait approach integrating AF PGS with data from multiple correlated traits, similar to the GPSmult and PRSmix+ methods, which improved coronary artery disease (CAD) prediction^2,19^ across various ancestry groups.

Using training data from 269,746 AF cases and nearly 10 million individuals, and leveraging GWAS data from correlated traits, we developed ancestry-optimized multi-PGSs and tested these in the diverse All of Us Research Program, BioBank Japan (BBJ), and several additional cohorts. Our goal was to assess and improve genomic prediction of AF across ancestries, thereby providing a more equitable and interpretable foundation for clinical translation.

## Results

### Creating and testing multi-ancestry and multi-method AF PGS

To generate our PGSs for AF, we first meta-analyzed large-scale GWAS data from the AFGen consortium^12^ (*N* = 181,446 cases) with GWAS data from the MVP^16^ (*N* = 88,300 cases), creating large ancestrally-diverse GWAS datasets for AF (Supplementary Note 3). Of note, while we acknowledge that ancestry is not truly categorical^20^, data were grouped into distinct continental ancestries; these groups were largely based on genetically-determined similarity to such continental ancestries: European (EUR), Admixed-American (AMR), African (AFR), South-Asian (SAS), and East-Asian (EAS) ancestry groups. Building on the GWAS meta-analyses, we applied the SBayesRC algorithm^18^ to the ancestry-specific GWAS summary statistics, resulting in EURmeta, AMRmeta, and AFRmeta PGSs; we also used SBayesRC to create an all-ancestry PGS (ALLmeta), using the pan-ancestry GWAS meta-analysis as training data (Supplementary Fig. 1; “Methods”).

PGS tuning and validation was initially focused on genetically-determined EUR, AMR, and AFR ancestry groups in the All of Us Research Program (Supplementary Fig. 1; Supplementary Data 1), an ancestrally-diverse cohort from the United States^20–22^. Interestingly, we found that the ancestry-specific scores did not consistently outperform the gold-standard PGS from Roselli et al. This was reflected by ΔOR/SD values among EUR (+0.13), AMR (−0.26), and AFR (−0.04) validation sets (Supplementary Fig. 2; Supplementary Data 2). Therefore, we used the multi-PGS framework^18^ from SBayesRC to combine each ancestry-specific score (e.g., EURmeta or AFRmeta) with the all-ancestry PGS (ALLmeta), using weights derived from the joint predictive performance in a tuning set (Supplementary Fig. 1).

The resulting multi-ancestry - or “Mult-a” - PGSs outperformed the Roselli PGS across EUR, AMR and AFR ancestry, in the validation set (Supplementary Fig. 2). Specifically, the OR per SD increase of the PGS improved from 1.59 to 1.82 in EUR, from 1.34 to 1.39 in AMR, and from 1.21 to 1.30 in AFR. Interestingly, we found that the Mult-a scores showed nearly identical predictive accuracy relative to the ALLmeta score alone, showing similar prediction within EUR, AMR and AFR ancestry validation sets (Supplementary Fig. 2; Supplementary Data 2).

We also explored a similar pipeline to combine the ALLmeta score (based on SBayesRC) with PGSs derived from several other statistical architectures^23^ (CT-SLEB and PROSPER)^24,25^, which integrate both ancestry-specific and all-ancestry training data, using a custom multi-PGS tool (Supplementary Fig. 3; “Methods”). Specifically, this generated multi-method or “Mult-m” scores (Supplementary Fig. 4). Mult-m scores provided small improvements in EUR and AMR groups—and slightly worse prediction in AFR - at the cost of substantial analytical and computational complexity (Supplementary Fig. 5; Supplementary Data 3). Given the limitations of the Mult-a and Mult-m approaches, we consequently continued model development using the simpler ALLmeta PGS.

### Developing and testing multi-trait PGS for AF

In the next stage, we integrated the ALLmeta PGS with PGSs for seven traits genetically-correlated with AF (“Methods”). Similar to the Mult-a and Mult-m approaches, PGSs were combined using performance-based weights (or mixing weights) from tuning sets in All of Us. We evaluated two tuning strategies for constructing the multi-trait—or “Mult-t”—scores: one using ancestry-specific tuning and another using all-ancestry tuning (Supplementary Fig. 6; “Methods”). As expected, the ancestry-tuned (or ancestry-optimized) Mult-t scores consistently outperformed the all-tuned versions across the EUR (ΔOR/SD = 0.01) and AMR (0.03) validation sets, with the strongest improvement observed in the AFR group (0.07) (Supplementary Fig. 7; Supplementary Data 4). Based on these results, we selected the Mult-t-EUR, Mult-t-AMR, and Mult-t-AFR scores as the final models for the respective ancestries.

**Fig. 1:**
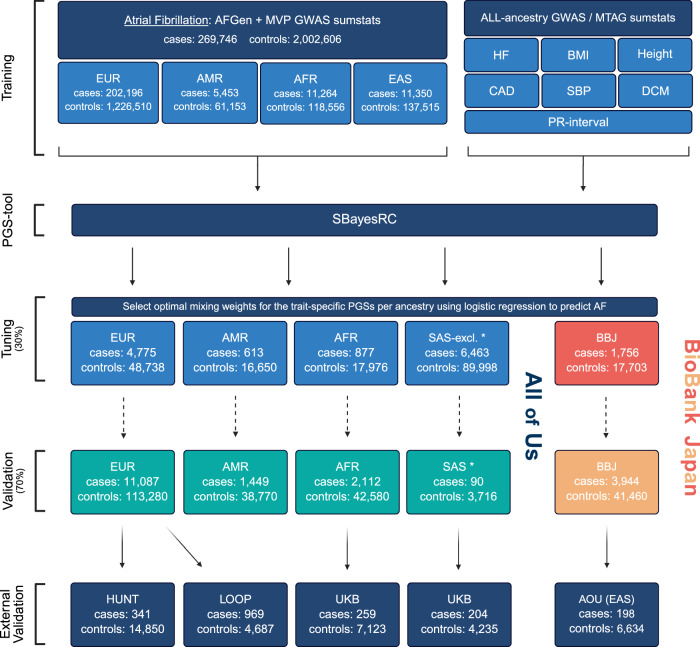
Multi-trait polygenic score development and testing, an overview. This flowchart illustrates the development of the Mult-t PGS across five key stages: Training, PGS tool, Tuning (30%), Validation (70%), and External Validation. The scores were derived from (i) all-ancestry GWAS summary statistics for AF from the AFGen and MVP cohorts across four ancestral groups (EUR, AMR, AFR, and EAS; top left), and (ii) all-ancestry training data from seven traits correlated with AF (top right). SBayesRC was used to generate polygenic score files for each trait. These were combined in five different ways using unadjusted logistic regression models to predict AF in each ancestry-specific tuning set, and weights were given based on that. The resulting ancestry-tuned multi-trait PGSs were evaluated in the corresponding ancestry-specific testing sets using logistic regression adjusted for the first 20 PCs, age and sex. Tuning and validation were primarily performed in the All of Us dataset; due to limited South Asian (SAS) sample size, SAS samples were used exclusively for validation, while 30% of EUR, AMR, and AFR samples and 100% of EAS samples were used for tuning (SAS-excl.). For EAS, tuning and validation were performed in BioBank Japan. External validation was conducted in HUNT (Norway), LOOP (Denmark; clinical trial), UK Biobank (AFR and SAS), and All of Us (EAS). Ancestries: EUR European, AFR African, AMR Admixed American, EAS East Asian, SAS South Asian, SAS-excl. SAS-excluded. Traits: HF heart failure, BMI body mass index, Height, CAD coronary artery disease, SBP systolic blood pressure, DCM dilated cardiomyopathy. Cohorts: AOU All of Us Research Program, BBJ BioBank Japan, HUNT Trøndelag Health Study, LOOP Implantable loop recorder detection of atrial fibrillation to prevent stroke, UKB UK Biobank. Created in BioRender. Lahrouchi, N. (2026) https://BioRender.com/4tz6zwj.

We then aimed to create similar multi-trait PGSs for Asian ancestry groups. For SAS, a unique strategy was applied to ensure a sufficiently large validation set: We used a multi-ancestry tuning set from All of Us excluding SAS individuals, enabling evaluation in a 100% SAS validation cohort (Supplementary Data 1 and 4). For development of Mult-t-EAS, we turned to BBJ (Supplementary Data 5)^26^, where we followed a 30% tuning and 70% testing approach (Fig. 1) with additional validation in EAS participants from All of Us (Supplementary Data 1).

An overview of the final PGS construction, including case/control counts for the training, tuning, and validation sets, is provided in a flowchart (Fig. 1). Additional details on the GWAS training data, including GWAS data for the correlated traits, are available in Supplementary Data 6.

### Enhanced prediction of AF using multi-trait PGS

**Fig. 2:**
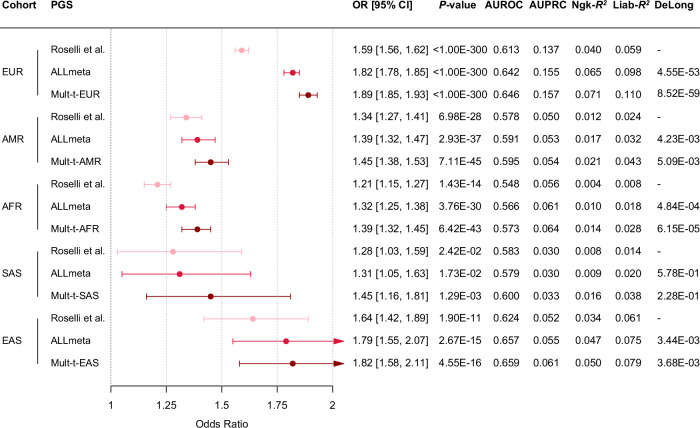
Comparison of the Roselli et al., ALLmeta, and Mult-t PGSs for AF prediction in All of Us. The left panel displays a forest plot of the OR/SD increase in PGS for AF, with 95% CIs on the x-axis (Supplementary Data 7). The y-axis lists ancestry-specific cohorts from the All of Us validation dataset (Supplementary Data 1): EUR (*n* = 124,367 of which 11,087 cases), AMR (*n* = 40,219 of which 1449 cases), AFR (*n* = 44,692 of which 2112 cases), SAS (*n* = 3806 of which 90 cases), and EAS (*n* = 6832 of which 198 cases), each paired with its PGSs. EUR, AMR, and AFR are based on the 70% validation subset, whereas the full 100% validation set was used for SAS and EAS due to limited sample size. The right panel summarizes key performance metrics. All estimates are based on logistic regression models adjusted for the first 20 PCs, age, and sex, except for AUROC and AUPRC values which are based on univariate models (to show prediction from PGS alone). *P*-values were calculated using two-sided Wald tests, except for comparisons with the Roselli et al. PGS, which used a one-sided DeLong test. Source Data are provided (Supplementary Data 7).

We assessed performance of the final multi-trait PGSs compared to the ALLmeta and Roselli et al. PGSs in All of Us. Across all ancestry groups, the ALLmeta score outperformed the previously published Roselli PGS, when judged by various performance metrics in the validation sets (Fig. 2; Supplementary Data 7; Supplementary Fig. 7). The Mult-t scores consistently demonstrated further improvements over the ALLmeta score, although confidence intervals were wide for EAS and SAS ancestry due to small case numbers.

Specifically, multi-trait PGS performance was highest in EUR (OR/SD = 1.89 [1.85–1.93]; AUROC = 0.646 [0.64–0.65]; AUPRC = 0.157) and EAS (1.82 [1.58–2.11]; 0.66 [0.62–0.70]; 0.061) validation sets, followed by AMR (1.45 [1.38–1.53]; 0.60 [0.58–0.61]; 0.054), SAS (1.45 [1.16–1.81]; 0.57 [0.54–0.66]; 0.033), and AFR (1.39 [1.32–1.45]; 0.57 [0.56–0.59]; 0.064) ancestries. The largest absolute gains over the Roselli score were also observed in EUR (ΔOR/SD = 0.30; ΔAUROC = 0.033; ΔAUPRC = 0.020) and EAS (0.18; 0.035; 0.009) validation sets, followed by AFR (0.18; 0.025; 0.008), SAS (0.17; 0.017; 0.003), and AMR (0.11; 0.017; 0.004) (Fig. 2; Supplementary Data 7; Supplementary Fig. 7).

**Fig. 3:**
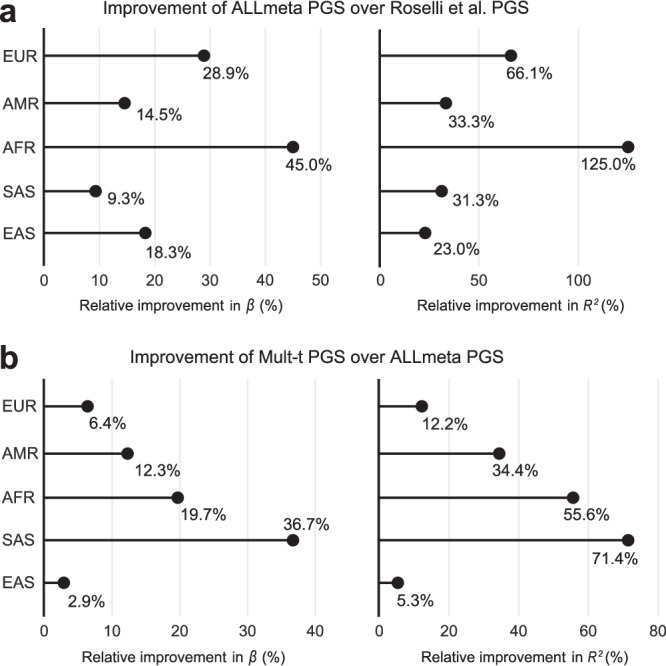
Relative improvements of our PGSs per ancestry group in All of Us. All panels represent lollipop charts, with relative improvements (%) in prediction on the x-axes and different ancestry groups from the All of Us validation dataset (Supplementary Data 1) on the y-axes: EUR (*n* = 124,367 of which 11,087 cases), AMR (*n* = 40,219 of which 1449 cases), AFR (*n* = 44,692 of which 2112 cases), SAS (*n* = 3806 of which 90 cases), and EAS (*n* = 6832 of which 198 cases). EUR, AMR, and AFR are based on the 70% validation subset, whereas the full 100% validation set was used for SAS and EAS due to limited sample size. In both parts, the left plot shows prediction as measured by logistic regression beta coefficients, while the right plots shows prediction as measured by liability-scale variance explained. Part **a** shows the relative prediction improvements of the ALLmeta SBayesRC score over the Roselli et al. PGS, while part **b** shows relative prediction improvements of our ancestry-optimized Mult-t PGSs over the ALLmeta PGS. All estimates are based on logistic regression models adjusted for the first 20 PCs, age, and sex. Source Data are provided (Supplementary Data 8).

Nevertheless, we found that our multi-trait approach provided relatively larger improvements in ancestries with worse baseline prediction (Fig. 3; Supplementary Data 8). For instance, when comparing the Mult-t scores to the ALLmeta score, relative improvements were strongest for SAS (beta improvement = 36.7%; AUROC improvement = 26.6%; liability-scale*R²* improvement = 71.4%), AFR (19.7%; 10.6%; 55.6%) and AMR (12.3%; 4.4%; 34.4%), with only minor relative improvements in EUR (6.4%; 2.8%; 12.2%) and EAS (2.9%; 1.3%; 5.3%).

Taken together, these results demonstrate that leveraging all-ancestry training data, paired with a multi-trait framework, improves genomic AF prediction across ancestries compared to the existing gold standard. While absolute performance remains highest in Europeans, the multi-trait approach offers particularly meaningful improvements in non-EUR populations with weaker baseline prediction.

### Comparison to PRSmix+

MGB will be implementing a PGS built using the PRSmix+ approach^2^, which also uses a multi-trait framework. We therefore also assessed the currently available PRSmix+ PGS for AF, making sure to exclude samples from All of Us that were potentially used in model development (Supplementary Fig. 8). We found that the PRSmix+ PGS marginally outperformed the Roselli et al. score across ancestries, although notably the Mult-t PGSs outperformed the available PRSmix+ score (Supplementary Fig. 8; Supplementary Data 9).

### Decomposition of the Mult-t scores by trait

**Fig. 4:**
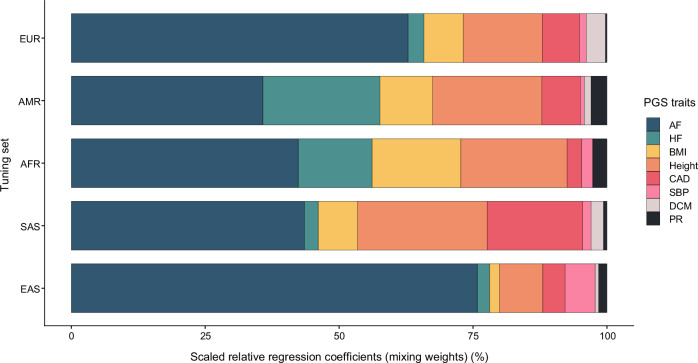
Scaled relative regression coefficients of the Mult-t PGSs across ancestry-specific tuning sets. The plot illustrates the relative contribution of each of the eight traits (legend) to the final Mult-t scores across ancestry-specific tuning sets (y-axis): EUR (*n* = 53,513 of which 4775 cases), AMR (*n* = 17,263 of which 613 cases), AFR (*n* = 18,853 of which 877 cases), SAS (*n* = 96,461 of which 6463 cases), and EAS (*n* = 19,459 of which 1756 cases). EUR, AMR, and AFR are based on the 30% tuning subset in All of Us, whereas the SAS tuning set combines these subsets with 100% of EAS samples (Supplementary Data 1). EAS is based on its 30% tuning subset in BioBank Japan (Supplementary Data 5). The x-axis represents the mixing weights, scaled regression coefficients expressed as percentages and normalized to 100% to reflect the trait composition of the Mult-t PGSs. Mixing weights were derived from unadjusted logistic regression models based on each trait’s predictive accuracy for AF in the corresponding tuning set. Source Data are provided (Supplementary Data 10). Unscaled coefficients are available in Supplementary Fig. 9.

To better understand the predictive basis of the Mult-t PGSs, we examined the components and relative contributions (Fig. 4). In each tuning set, the AF PGS contributed the most to the Mult-t score (36–76%) (Supplementary Data 10 and 11). Height was the second-largest contributor in most ancestry groups (15–24%), except in AMR, where heart failure (HF) ranked second. The third-largest contributor varied across HF, body mass index (BMI), and CAD. AF had the highest relative contribution in the EUR and EAS tuning sets compared to the other ancestry groups, while HF, BMI, and CAD had the largest relative contribution in AMR (21.8%), AFR (16.6%), and SAS (17.8%) compared to the other ancestry groups, respectively. Overall, the heterogeneity in trait contributions highlights that the correlated traits capture ancestry-relevant effects on AF risk that are not fully reflected by the AF-derived PGS alone.

We also evaluated the trait-specific PGSs individually in the ancestry-specific validation sets of All of Us, to better understand the contributions of these PGSs to AF risk (Supplementary Figs. 10–14; Supplementary Data 12). AF PGS generally showed the strongest predictive performance across all ancestries, particularly in EUR. HF PGS ranked second (1.2–1.5) and BMI PGS third (1.2–1.4) in most ancestry groups. DCM PGS and PR interval PGS generally had minimal predictive value across ancestries.

### Validation of multi-trait PGS in additional cohorts

**Fig. 5:**
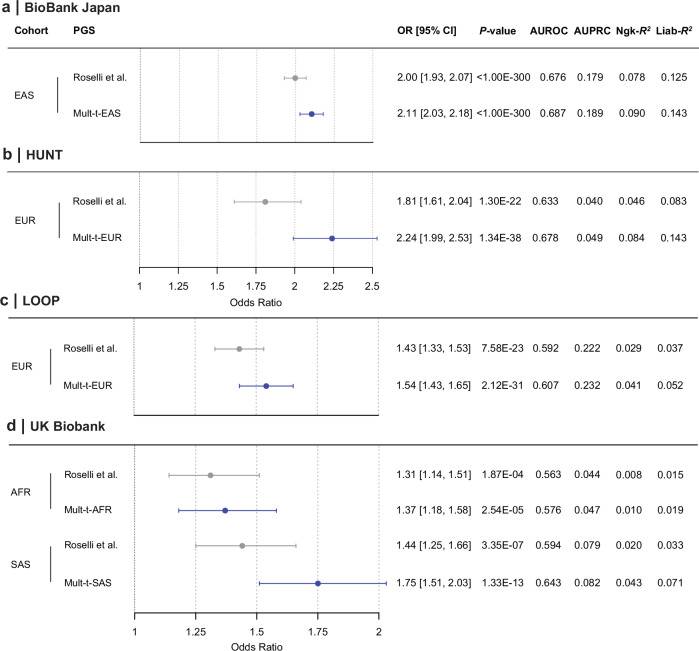
Comparison of the Roselli et al. and Mult-t PGSs for AF prediction across four additional datasets. In each panel, the left part displays a forest plot of the OR/SD increased risk of AF, with 95% CIs, on the x-axis (Supplementary Data 13–16). The y-axis displays the respective ancestry groups tested, as well as the specific PGSs assessed. The right part summarizes key performance metrics. All estimates are based on logistic regression models adjusted for relevant covariates, including ancestry PCs, age and sex, except for AUROC and AUPRC values which are based on univariate models (to show prediction from PGS alone). *P*-values were calculated using two-sided Wald tests. **a** shows results from EAS ancestry samples within BioBank Japan (*n* = 45,404 of which 3944 cases); **b**, **c** show results for EUR ancestry samples from HUNT 4th survey (*n* = 15,191 of which 341 cases) and LOOP (*n* = 5656 of which 969 cases), respectively; **d** shows results from AFR (*n* = 7382 of which 259 cases) and SAS (*n* = 4439 of which 204 cases) ancestry from UK Biobank. In (**d**), PGSs are based on simplified forms to prevent overfitting (“Methods”). Source Data are provided (Supplementary Data 13–16).

In the validation set of BBJ (Fig. 5a), we observed a strong performance of the Mult-t-EAS score for prediction of AF (OR/SD 2.11; AUROC 0.687), which improved over the Roselli et al. score (ΔOR/SD = 0.10; ΔAUROC = 0.011; ΔAUPRC = 0.010). Mult-t-EAS only minimally improved over ALLmeta, likely because this PGS already provided strong performance in this dataset (Supplementary Data 13).

We also validated the Mult-t-EUR score in external cohorts (Fig. 1). In the Norwegian HUNT4 cohort, a population-based cohort from Norway^27^, we observed a stepwise improvement in predictive performance from the Roselli score to the Mult-t-EUR score, which demonstrated substantial performance for AF prediction in this cohort (OR/SD = 2.24; AUROC = 0.678) (Fig. 5b; Supplementary Data 14).

We also assessed the Mult-t-EUR score in the LOOP study, a Danish clinical trial assessing implantable loop recorder (ILR) screening in older participants without AF but at elevated cardiovascular risk^28^. In this high-risk population, the Mult-t-EUR score strongly outperformed the Roselli score (OR/SD = 1.54; AUROC = 0.607; Fig. 5c; Supplementary Data 15). Furthermore, recapitulating recent findings^29^, we found that ILR screening was associated with lower rates of stroke or systemic embolism in participants with higher Mult-t-EUR PGS (HR = 0.66, *P* = 0.048), with no benefit in participants with lower Mult-t-EUR PGS (HR = 1.00, *P* = 0.98) (Supplementary Fig. 15).

We then aimed to evaluate the performance of Mult-t-AFR and Mult-t-SAS in the UK Biobank. Given that non-European UK Biobank participants contributed to some of the PGSs incorporated into the Mult-t scores, we assessed simplified scores that excluded these specific PGSs (“Methods”). Among individuals of AFR and SAS ancestry in UK Biobank, the simplified Mult-t scores outperformed the Roselli et al. score in AFR and SAS ancestry individuals, respectively. The most pronounced improvement was observed in SAS ancestry (ΔOR/SD = 0.31; ΔAUROC = 0.049; ΔAUPRC = 0.003). In AFR, performance was also nominally improved (ΔOR/SD = 0.06; ΔAUROC = 0.013; ΔAUPRC = 0.003) compared with the Roselli et al. PGS (Fig. 5d; Supplementary Data 16).

Taken together, across the additional validation cohorts of diverse ancestries, we find consistent patterns of improved prediction of AF with the Mult-t scores over the Roselli et al. score. The general trend indicates that the multi-trait scores yield stable and transferable prediction improvements over the current gold-standard PGS for AF.

### Risk stratification at the extremes of the PGS distribution

**Fig. 6:**
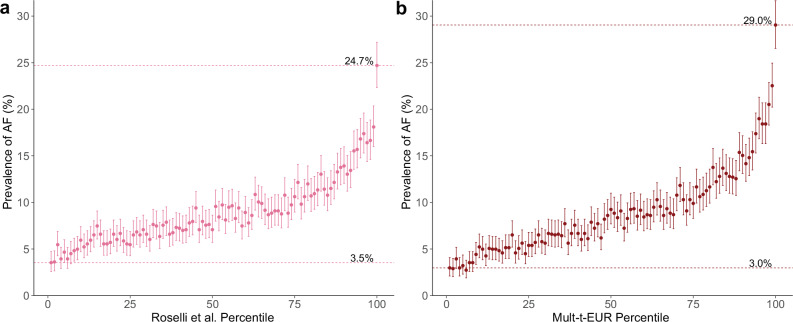
AF prevalence across 100 PGS percentile groups among European individuals from All of Us. Each panel shows the PGS percentile on the x-axis and AF prevalence with 95% CI on the y-axis (Supplementary Data 17). Dotted lines indicate AF prevalence in the lowest (bottom) and highest (top) percentiles. Participants were grouped by percentile using either the Roselli et al. PGS in pink (**a**) or the Mult-t-EUR PGS in dark red (**b**). Both scores were evaluated in the 70% European ancestry validation dataset of All of Us (*n* = 124,367 of which 11,087 cases). Source Data are provided (Supplementary Data 17).

To further benchmark the performance of our EUR PGS, we performed several additional analyses to directly compare the Mult-t-EUR PGS and the Roselli PGS, within the EUR validation set of All of Us. First, we found that AF cases on average had higher Mult-t-EUR PGS values than Roselli et al. PGS values, with no difference among controls (Supplementary Fig. 16). Second, when plotted by PGS percentile, we observed better stratification across the continuum of PGS values (Fig. 6; Supplementary Data 17): Prevalence ranged from 3.0% in the lowest percentile to 29.0% in the highest percentile, compared to 3.5% and 24.7% for the Roselli PGS.

**Fig. 7:**
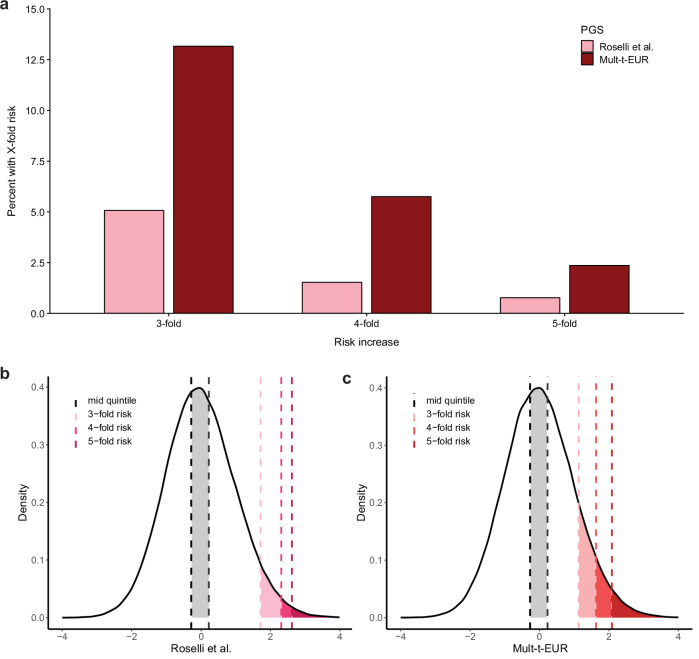
Proportion of European individuals from All of Us with 3-, 4-, or 5-fold increased AF risk compared to the middle quintile. All panels show the percentage of European ancestry participants in the 70% validation dataset of All of Us (*n* = 124,367 of which 11,087 cases) with a 3-, 4-, or 5-fold higher risk of AF relative to the middle PGS quintile. **a** Bar plots display the proportions in each risk group for the Roselli et al. PGS (pink) and the Mult-t-EUR PGS (dark red). **b**, **c** Density plots of the Roselli et al. (**b**) and Mult-t-EUR (**c**) PGSs. The x-axis displays polygenic score values, and the y-axis indicates density, representing the relative frequency of individuals with a specific polygenic score value. The middle quintile is shaded in gray, while regions corresponding to 3-, 4-, and 5-fold risk are highlighted in progressively darker tones. Odds ratios were estimated using logistic regression adjusted for age, sex, and the first 20 ancestry PCs. Source Data are provided (Supplementary Data 18).

Third, to further evaluate stratification at the extremes of the PGS distribution, we compared AF risk for individuals in the tails of the PGS distribution to those in the middle quintile, using logistic regression (“Methods”). We found that the Mult-t-EUR PGS captured a larger segment of the population at elevated risk than the Roselli score (Fig. 7a; Supplementary Data 18). Specifically, Mult-t-EUR could identify 13.2%, 5.8%, and 2.4% of individuals at 3-, 4-, and 5-fold increased odds of AF compared to the median, as compared to only 5.1%, 1.5%, and 0.8% with the Roselli PGS (Fig. 7b, c; Supplementary Data 18). We applied a similar approach to identify individuals with substantially reduced risk. The Mult-t-EUR PGS identified 7.0% of individuals at ⅓-fold odds of AF, compared to only 0.6% with the Roselli PGS (Supplementary Fig. 17).

Given the strong prediction of the Mult-t-EAS score in BBJ, we repeated similar analyses for Mult-t-EAS in the BBJ validation set. Here, stratification at the extremes was even more pronounced, finding 22.6%, 11.3%, and 6.6% of individuals with at least 3-fold, 4-fold, and 5-fold elevated risk compared to the median (which improved over Roselli; Supplementary Fig. 18); we identified 9.1% at ⅓-fold risk (substantially improving over Roselli; Supplementary Fig. 19). These results indicate enhanced risk stratification for Mult-t-EUR and Mult-t-EAS at both ends of the distribution, as the scores more effectively shift high-risk individuals toward the upper tail and low-risk individuals toward the lower tail, while reducing their presence in the middle quintile.

### Integration of PGS into existing clinical risk models

Finally, we assessed whether our PGSs improve prediction of AF on top of existing clinical risk prediction tools. Within the All of Us validation sets, focusing on incident AF, we found that both the ALLmeta and Mult-t scores remained strongly associated with AF when conditioned on the CHARGE-AF clinical model^30^, across EUR, AMR and AFR ancestries (Supplementary Figs. 20 and 21; Supplementary Data 19 and 20). We found that Hazard ratios for the Mult-t scores were more attenuated than for ALLmeta upon conditioning for CHARGE-AF; nevertheless, Mult-t scores still yielded higher Hazard ratios than ALLmeta across the three ancestry groups, even after conditioning (Supplementary Fig. 20; Supplementary Data 19). Similar patterns were observed for the HARMS2-AF clinical risk score^31^ (Supplementary Figs. 22 and 23; Supplementary Data 21 and 22).

In terms of C-index, we found that the addition of ALLmeta or Mult-t scores to CHARGE-AF improved prediction of incident AF (Supplementary Fig. 21; Supplementary Data 20). For instance, the C-index among EUR participants increased from 0.80 (95%CI [0.78; 0.82]) to 0.82 ([0.80; 0.84]) when adding Mult-t-EUR to the model, a significant difference by Noether’s test (one-sided *P* = 7.7 × 10^−34^; Supplementary Data 23). For AMR and AFR, non-significant (one-sided *P* = 0.09) and significant (one-sided *P* = 8.2 × 10^−5^) improvements were observed upon adding Mult-t-AMR and Mult-t-AFR, respectively. The Mult-t scores also improved over ALLmeta PGS in models alongside CHARGE-AF (one-sided *P* < 0.05 in EUR and AFR). Similar patterns were observed for the HARMS2-AF clinical score (Supplementary Data 23). These findings indicate that our PGSs represent independent risk indicators that improve discrimination of AF alongside clinical risk models. Future work should now assess whether clinical model-informed reweighting of PGS components may improve prediction further.

### Sensitivity analyses

Several additional sensitivity analyses and subgroup analyses (including sex-specific analyses), performed within the All of Us cohort, are provided in the supplements (Supplementary Figs. 24 and 25; Supplementary Data 24 and 25).

## Discussion

In this study, we aimed to construct improved PGSs for AF by combining several large multi-ancestry GWAS datasets with multiple methodological approaches. First, we used the SBaysesRC algorithm to create dense genome-wide PGSs from GWAS data from the AFGen Consortium and MVP. We found that the SBayesRC score, trained on a pan-ancestry meta-analysis, led to improved prediction as compared to the previous publicly-available gold-standard PGSs. We subsequently evaluated three complementary ensemble strategies for PGS construction: A multi-ancestry, a multi-method, and a multi-trait approach. The multi-trait approach in particular provided improved prediction for populations with worse baseline prediction (e.g., AMR, AFR, and SAS), although prediction for these groups was still diminished as compared to EUR and EAS ancestries. These findings allow for several conclusions.

First, we developed (to our knowledge) the strongest PGS for AF among EUR and EAS ancestries, to date. The Mult-t-EUR and Mult-t-EAS PGSs both demonstrated predictions with OR/SD of ~1.8–2.2 and AUROC of ~0.65-0.68, within population-based EUR and EAS datasets. Notably, both scores showed improved case stratification in a direct comparison to the PRSmix+ PGS^2^ and the Roselli et al. PGS^12^ (the latter shown to strongly improve over the widely-used^32,33^ PGS from Khera et al.^34^; Supplementary Note 1). Our PGSs provided improved prediction both across the continuous score distribution and when comparing extremes to the median. For instance, in the extremes, we identified ~6% of EUR individuals and ~11% of EAS individuals at over 4-fold risk of AF. These effects are comparable to the strongest known clinical predictors of AF, such as heart failure (OR = 3.6)^35^, and to pathogenic rare variants in *TTN* (OR/HR = 2.06–4.41)^36–38^, which have lower prevalences (~5% in older individuals^39^ and 0.44% in the general population^40^, respectively). Moreover, Mult-t-EUR explained 11-14% of AF variance on the liability scale, as compared to 6–8% explained by the Roselli et al. PGS. Nevertheless, we note that the Mult-t-EUR score still only captures ~50-60% of the estimated common variant heritability of AF in EUR (~22%^11^). These findings therefore illustrate how improved GWAS data and PGS methodology continue to improve the genetic prediction of AF, even in EUR, with additional improvements likely still possible.

Second, our PGS framework provides important improvements to the genetic prediction of AF in underrepresented populations. Notably, aside from EAS, the final Mult-t scores also outperformed the Roselli et al. score across ancestries where baseline prediction was poor, reaching prediction with OR/SD of ~1.5 in AMR, ~1.4 in AFR, and ~1.5–1.8 in SAS. Our PGSs also surpassed recent multi-ancestry scores for AF developed by Gunn et al.^41^. (Supplementary Note 1). Consistent with data from Gunn et al., and consistent with data from other heritable traits^41,42^, we observed that all-ancestry training data improved AF prediction across ancestries. Nevertheless, prediction remains relatively diminished in these ancestries, particularly in AMR and AFR. This finding may partially be explained by the relatively small sample size of non-European GWAS datasets for AF, as well as poor transferability of European GWAS data to these ancestry groups^20,43,44^. In this light, we note that most AF PGSs currently available in the PGS Catalog remain derived from EUR cohorts^45–48^. Therefore, our ancestry-optimized PGSs represent an important step towards more equitable genomic prediction of AF, especially for Asian ancestry groups, although our findings also highlight the ongoing need for additional diverse GWAS cohorts.

Third, our work shows how PGS performance can be enhanced by integrating correlated traits within a multi-PGS framework. Specifically, we used a custom adaptation of the SBayesRC-multi tool (Code Availability), which allowed for integration and ancestry-specific tuning of PGSs for multiple AF-related traits. This multi-trait approach yielded the largest relative gains among SAS (71% improvement in *R*^2^), AFR (56% improvement), and AMR ancestries (34% improvement). These findings suggest a particular utility of multi-trait PGSs, paired with ancestry-specific tuning, in underrepresented populations (Supplementary Note 1). We note that multi-trait approaches for PGSs have been explored in previous literature. For instance, one study applied LASSO to combine 937 European-trained PGSs, which yielded improved genomic prediction for several psychiatric disorders^49^. A landmark study on CAD integrated ancestry-specific PGSs and genetically-correlated traits - using stepwise model selection (stepAIC) within a European tuning set^19^—leading also to substantially improved prediction. For AF, prior multi-trait approaches have used elastic net regression, including RFDiseasemetaPRS (OR/SD = 1.30)^50^ and PRSmix+^2^. However, both of these models were evaluated exclusively in EUR individuals in prior work. In contrast, our study shows the value of ancestry-informed multi-trait approaches for genomic prediction of AF across diverse populations.

We note that the PRSmix+ PGS represents a score similar to the PGS that will be implemented clinically at MGB, as of late 2025^5,6^. Importantly, our Mult-t PGS substantially outperformed the currently-available PRSmix+ PGS for AF (PGScatalog: PGS004706). Nevertheless, given that PRSmix+ inherently leverages prediction power from other PGSs^2^, we acknowledge that future ancestry-specific optimization of PRSmix+—incorporating also our PGSs—should prove fruitful to improve genomic prediction of AF further.

Fourth, our analyses highlight the power of the initial SBayesRC model^18^, a genome-wide Bayesian approach that incorporates functional annotations and leverages dense genome-wide markers. In previous work, SBayesRC showed superior prediction, compared to several other methods, across many heritable traits^23^. While genome-wide PGSs have previously been developed for AF^34,51^, most recent AF scores in the PGS Catalog remain limited, either not genome-wide or based on only ~1 M SNVs^45,46,52^. In contrast, leveraging ~7 M functionally-annotated variants, as we did, has been shown to substantially improve predictive accuracy as compared to using 1 M unannotated SNVs^18^, further supported by substantially improved prediction using the SBayesRC model for AF in our analyses. Several other technical aspects of our PGS methodology are further discussed in the Supplementary Note 1.

We note that our study has several limitations. First, despite focusing on genetically-predicted continental ancestries, results for several non-European groups (AMR, AFR, and SAS) are based on U.S. and European cohorts. As such, some degree of EUR admixture is possible for these participants^53,54^. Additional work in continental cohorts is therefore required to extend our findings, in the future. Second, in contrast to our multi-trait approach, the SBayesRC algorithm actually led to relatively large prediction improvements among EUR, despite our goal to reduce disparities. This likely reflects the large contribution of EUR to our base GWAS; indeed, we observed a specific order in the Mult-t score’s predictive performance in All of Us (EUR > EAS > SAS > AMR > AFR), a pattern that loosely mirrors the ancestry sample sizes reported in the base GWAS data and other large genetic datasets^55,56^. Nevertheless, prediction in AFR lags behind AMR and SAS despite larger GWAS case numbers, a finding potentially due to the greater genetic diversity and shorter haplotype blocks seen in Africans^57^. Differences in European admixture may also affect AMR and AFR ancestries specifically^53,54^. In any case, similarly reduced performance in AMR and AFR groups has been reported in two CAD PGS studies^19,42^.

Looking ahead, we again stress that more adequately powered and diverse GWASs are needed, guided by key strategies outlined in a prior study^56^. This is particularly critical for AMR, AFR and SAS ancestries, which could be better represented through expanding efforts such as the Mexico City Prospective Cohort Study^58^, MVP^59^, H3Africa^60^, and the Genes & Health initiative^61^, respectively, each of which require sustained funding to grow. Additionally, statistical methods that enhance cross-ancestry portability, such as SBayesRC, along with diverse evaluation cohorts like All of Us^62^, are essential for completing the PGS development process. In a similar fashion, additional work should assess how sex-stratified and sex-optimized PGSs may improve individualized genomic prediction (Supplementary Note 1).

As genomic prediction of AF improves, and as AF PGSs enter the clinic, it has to be considered how PGSs may be used to concretely improve AF care or screening. Consistent with our findings, AF PGS has been shown to enhance risk prediction when added to clinical models such as HARMS2-AF and CHARGE-AF in other population-based cohorts^14^, and in a clinical cohort of cardiovascular disease patients, where combining an AF PGS with CHARGE-AF and NT-proBNP improved the C-index from 0.67 to 0.70^63^. In CAD, prospective studies in primary care have demonstrated that adding PGSs to clinical scores improves prediction and is generally well-accepted by both clinicians and patients^64,65^. For AF, a care pathway can be envisioned where individuals with a high PGS are prioritized for aggressive risk factor management and/or earlier routine AF screening^66^, potentially to prevent strokes^29^. Still, further research in real-world settings is needed to assess the clinical utility, implementation, and ethical considerations of AF PGSs across diverse populations. At the same time, the field will need to firmly establish those clinical use-cases where PGS implementation stands to change standard care^1^.

In conclusion, we demonstrate that integrating diverse GWAS data and correlated traits in a multi-PGS framework substantially improves genomic prediction of AF. Our multi-trait approach was particularly effective for individuals of Asian genetic ancestry, with further gains expected in other non-European ancestries as diverse training datasets expand. As AF PGSs enter the clinic, our ancestry-optimized scores may lay the groundwork for more equitable and interpretable genomic prediction of AF.

## Methods

### Ethics statement

This study was conducted in accordance with all relevant ethical regulations, and approved by the institutional review boards and ethics committees of all participating cohorts, as detailed in the “Ethics” section.

### Meta-analysis of AF GWAS summary statistics

To improve the genomic prediction of AF across diverse ancestry cohorts, we increased the training sample size by combining GWAS summary statistics from the AFGen and MVP cohorts using METAL. This tool performed an inverse-variance weighted meta-analysis, accounting for differences in standard errors, sample sizes, and allele frequencies between studies. Details on METAL’s input parameters, the execution script, and file pre- and post-processing steps are provided in the Supplementary Note 3.

The following provides an overview of the cohorts included in the meta-analysis. AFGen, published by Roselli et al., is the largest meta-analysis to date focused on AF. It includes over 180,000 cases and approximately 1.5 million controls from multiple cohorts, with representation from EUR, AMR, AFR, and EAS ancestries^12^. The MVP is a U.S.-based cohort study comprising more than 635,000 individuals and providing GWAS summary statistics for over 1200 traits, including AF. MVP also includes participants from EUR, AMR, AFR, and EAS backgrounds, enabling analyses across diverse ancestries^16^. Details on the case and control counts across ancestries are provided (Supplementary Data 6). In this work, all reported *P*-values are two-sided, unless explicitly mentioned to be one-sided.

### Genome-wide PGS modeling using SBayesRC

To maximize the value of the diverse training data, we applied the SBayesRC algorithm (https://github.com/zhilizheng/SBayesRC)^67^, a Bayesian polygenic score tool that integrates functional genomic annotations to enhance prediction across ancestries. Specifically, SBayesRC (v0.2.6) incorporates data on 8,140,664 SNVs annotated with features such as enhancer and promoter status from the Baseline-LD v2.2^68^ model. For linkage disequilibrium (LD) reference, we used panels matched to the ancestry of the training data. Three LD panels were available, constructed from the imputed genotypes of UK Biobank participants of European (EUR; *n* = 347,800), African (AFR; *n* = 7006), or East Asian (EAS; *n* = 2252) ancestry. When no ancestry-matched LD panel was available, the EUR reference was used by default. An overview of SBayesRC’s input parameters, the execution script, and file pre- and post-processing steps is provided in the Supplementary Note 4.

### Main cohorts used for PGS development

Further development of our PGSs, including tuning and testing, was performed in two cohorts. First, we used data from the All of Us Research Program. All of Us is a U.S.-based initiative aimed at improving equity and diverse representation through a large-scale and ancestrally-diverse biomedical biobank with enrollment across the U.S^21,22^. For this study, we used version 8 of the dataset, including only individuals with both high-quality short-read whole genome sequencing (srWGS) and electronic health record data, representing 76.8% of the 410,401 srWGS participants. Participants were labeled and grouped according to their genetically-inferred similarity to major continental ancestry groups: European (EUR), Admixed American (AMR), African (AFR), South Asian (SAS) and East-Asian (EAS). Ancestry-specific case/control numbers for both sets are provided in Supplementary Data 1. AF cases were broadly defined to include both atrial fibrillation and atrial flutter, with exact definitions specified by OMOP IDs listed in Supplementary Data 26. Details on the All of Us variant- and sample-level quality control procedures and ancestry inference are provided in the Supplementary Note 2. In all analyses across all ancestry groups, unless otherwise specified, All of Us data were split into 30% tuning and 70% testing sets.

Second, we used data from the BBJ project for PGS development for EAS ancestry. To this end we used the BBJ 2nd cohort from the BBJ Project^26^, which was not included in the base AF GWAS or any of the other GWASs included in our approach. The BBJ is a hospital-based national biobank project that collects DNA and serum samples and clinical information from 12 cooperative medical institutes throughout Japan (Supplementary Note 2). BBJ 2nd cohort collected approximately 80,000 patients with 38 target diseases collected between 2013 and 2018 to expand research outcomes from the first cohort. Atrial fibrillation or atrial flutter were determined by the physician’s diagnosis or electrocardiogram records. Details on genetic quality control and processing are provided in the Supplementary Note 2. In all BBJ analyses, data were split into 30% tuning and 70% testing sets.

### Multi-ancestry and multi-method polygenic score development

We first aimed to construct multi-ancestry PGSs (Supplementary Fig. 1), and assess their performance through tuning and testing in All of Us. To achieve this, we applied SBayesRC to ancestry-specific GWAS summary statistics (EUR, AMR, AFR) from the AFGen and MVP meta-analysis. This generated ancestry-matched scores, denoted as EURmeta, AMRmeta, and AFRmeta. Subsequently, we used the SBayesRC-multi^18^ tool to combine each ancestry-specific score (e.g., EURmeta) with the all-ancestry score (ALLmeta). Specifically, we weighted both scores based on AF predictive performance in an ancestry-specific tuning set (Supplementary Fig. 1). A detailed description on the SBayesRC-multi method, including the pipeline script, is provided in the Supplementary Note 5. This approach was motivated by the hypothesis that combining the broad statistical power of the ALLmeta score with the ancestry-specific signal of, for example, the EURmeta score, while tuning within the corresponding ancestry group, would optimize predictive performance. Consequently, this approach yielded the multi-ancestry—or “Mult-a”—scores. Mult-a PGS development was performed in the All of Us Research Program, and assessed for EUR, AMR and AFR ancestry groups; these groups were chosen due to the availability of sufficient participants in these groups and the availability of ancestry-matched GWAS data.

Previous work has indicated that combining PGS from different statistical architectures can also improve prediction^23^. Mirroring the Mult-a approach, we explored combining our ALLmeta score (based on a Bayesian regression architecture^18^) with PGSs constructed using two different statistical approaches, namely CT-SLEB (built on clumping-and-thresholding architecture^24^) and PROSPER (built on LASSO architecture^25^). This approach yielded the multi-method—or “Mult-m”—scores. To this end, we adapted the SBayesRC-multi tool to allow more flexible input and output handling, including the capacity to handle more than two PGS inputs (Supplementary Fig. 3; Supplementary Note 5)^69^. Again, development was assessed for EUR, AMR, and AFR ancestry groups in All of Us. To avoid overfitting, a two-step tuning approach was applied for development of the Mult-m scores (Supplementary Fig. 4).

Interestingly, we found that the Mult-a scores provided nearly identical predictive performance of the simpler ALLmeta score in the EUR, AMR, or AFR validation subsets of All of Us, while the Mult-m scores yielded small but inconsistent benefits across ancestry groups at the cost of substantial additional analytical complexity (which would not allow for model development in smaller ancestry groups; “Results”). Therefore, we continued model development using the simple but effective ALLmeta PGS.

### Multi-trait polygenic score development

Multi-trait modeling has recently been proposed as a method to improve PGS prediction^2,19^. We therefore applied the adapted SBayesRC-multi tool to combine the ALLmeta score with all-ancestry trained PGSs from seven traits genetically-correlated with AF (Supplementary Fig. 6). The traits were manually selected based on having strong genetic correlation to AF^70,71^, and there being large-scale GWAS data available for those traits (HF, BMI, height, CAD, SBP, PR-interval). We also scanned the Cardiovascular Disease Knowledge Portal^72^ to identify additional traits with genetic correlation (rg > 0.3) with AF; DCM was additionally included, while many other traits were pragmatically excluded (due to a high similarity to traits already included, or due to small size of the available GWAS). While we acknowledge that trait selection was not systematic, we posit that the final selection is a good representation of important AF-comorbidities and risk factors. The case/control counts for the GWAS training data for AF and the correlated traits (HF, BMI, height, CAD, SBP, DCM, PR-interval) are listed in Supplementary Data 6.

Unlike the multi-ancestry scores, we aimed to integrate information across traits rather than ancestries, making it unclear whether ancestry-specific or all-ancestry tuning would yield better performance. Therefore, we explored both approaches, generating multi-trait—or “Mult-t”—scores from ancestry-specific tuning and from all-ancestry tuning. As expected, the ancestry-tuned Mult-t scores outperformed their all-tuned counterparts across the EUR, AMR, and AFR validation sets of All of Us (“Results”). Accordingly, the Mult-t-EUR, Mult-t-AMR, and Mult-t-AFR scores were selected as final models for these ancestries.

We then also aimed to apply similar approaches to generate Mult-t scores for EAS and SAS ancestry groups. Given small numbers in All of Us, the Mult-t-SAS score was tuned using an all-ancestry set that excluded all SAS individuals. This allowed us to retain a fully independent 100% SAS validation set, which provided sufficient power for evaluation. To develop a Mult-t-EAS score, we leveraged data from BBJ for both tuning and testing (Fig. 1), again following a 30% tuning and 70% testing scheme, with additional validation in All of Us.

### PGS computation, standardization and assessment

PGSs were computed using the --score function in PLINK2. To account for population structure, each PGS was regressed on the first 20 principal components (PCs) within the all-ancestry testing set, and the residuals were extracted to remove PC effects. The residualized PGSs were then standardized to mean zero and SD one, after which ancestry-specific subsets were extracted. Finally, the PGS’s ability to distinguish AF cases from controls was assessed using logistic regression, and several performance metrics were derived. The (log-)odds ratio, *P*-value and *R*^*2*^ were derived from multivariate logistic regression models corrected for age and sex; AUROC and AUPRC values were derived from univariate logistic regression models. For *R²*, we used Nagelkerke’s and liability *R²*, calculated as delta *R²* (Δ*R²* = *R²*_full − *R²*_base) divided by the residual *R²* (1 − *R²*_base). Liability *R²* was estimated using the ancestry-specific AF prevalence within the All of Us dataset^73^.

### Comparisons to previous AF PGS

To contextualize the predictive capacity of our PGSs, we also assessed several previously-published scores. In all analyses, we assessed the PGS developed by Roselli et al.^12^, obtained from the Cardiovascular Disease Knowledge Portal. In All of Us, we also assessed the recently developed PRSmix+ PGS for AF^2^, obtained through the PGScatalog (PGS004706).

### External validations

Outside of internal testing, we also seeked external testing/validation of our Mult-t scores across various population-based datasets (Fig. 1). First, we validated the Mult-t-EAS score (developed in BBJ) among EAS samples from All of Us. Second, we validated the Mult-t-EUR score among participants from the Trøndelag Health Study (HUNT); to avoid overfitting, we only used samples from the 4th survey of HUNT (which were not included in the base GWAS data; Supplementary Note 2). We also aimed to externally validate the Mult-t-AFR and Mult-t-SAS scores in the UK Biobank (UKB); since non-European samples from UKB contributed to some of the base GWAS, we assessed simplified models that excluded PGSs derived from these GWAS (Supplementary Note 2).

We also externally validated the Mult-t-EUR score in the Atrial Fibrillation Detected by Continuous ECG-monitoring Using Implantable Loop Recorder to Prevent Stroke in High-risk Individuals (LOOP Study)^29^. LOOP was a randomized clinical trial, including 6004 participants at risk of AF but without diagnosed AF, with 1:3 randomization to screening with an implantable loop recorder (ILR) and anticoagulation upon detection of AF or usual care (Supplementary Note 2). Alongside prediction of AF as described before, we also assessed whether mult-t-EUR was associated with improved utility of ILR for stroke prevention^29^.

### PGS prediction alongside clinical risk models

Finally, we assessed whether our PGS would improve AF prediction on top of existing clinical primary risk prediction models. To this end, we assessed EUR, AMR and AFR ancestry groups in the All of Us dataset, assessing the utility of our Mult-t scores (Mult-t-EUR, Mult-t-AMR, Mult-t-AFR) alongside both the CHARGE-AF and HARMS2-AF clinical scores^30,31^, using Cox time-to-event regression models, testing for incident AF events (events after enrollment to All of Us). Models including the PGS as the sole predictor were adjusted for age, sex, and the first 20 PCs. Models including a clinical risk score alone, as well as models jointly including a clinical risk score and a PGS (residualized for the first 20 PCs), were adjusted for the first 10 PCs. The phenotypic components used to derive the CHARGE-AF and HARMS2-AF clinical risk scores are described in detail in Supplementary Data 27. In particular, we assessed (i) the change in hazard ratios for PGS upon adjusting for either clinical risk model, and (ii) the change in model C-statistic upon addition of PGS to either clinical risk model.

### Ethics

This study was conducted in accordance with all relevant regulations governing the use of human participants and was performed in compliance with the Declaration of Helsinki. Informed consent was obtained from all participants across all cohorts used in this study. Access to the All of Us resource was granted by the All of Us Institutional Review Board, with analyses conducted under a data use agreement between Amsterdam UMC and the All of Us program. The BBJ project was approved by the ethics committees of the Institute of Medical Sciences at the University of Tokyo and the RIKEN Center for Integrative Medical Sciences. Use of UK Biobank resources was approved by the UK Biobank Research Ethics Committee, with UKB data accessed under approved application 176602. Use of the HUNT cohort was approved by the Regional Committee for Medical Research Ethics (2019/29771), the HUNT, the Norwegian Data Inspectorate, and by the National Directorate of Health. The study is in conformity with Norwegian laws and the Helsinki Declaration. The LOOP trial was approved by the local Ethics Committee and Data Protection Agency. Participant compensation varied by cohort and was determined by local ethics protocols, ranging from no financial compensation to reimbursement of expenses or in-kind participation benefits.

### Reporting on sex

Sex was genetically inferred within all cohorts used in this study. While the potential of sex-stratified PGSs are discussed (Supplementary Note 1), they were beyond the scope of this study, as their implementation would require separate scores for each sex, which introduces complexity in practical application.

### Figure generation and underlying analyses

Details on the creation of the main and Supplementary Figures, as well as the associated analytical procedures, are provided in the Supplementary Note 6.

### Reporting summary

Further information on research design is available in the Nature Portfolio Reporting Summary linked to this article.

## Supplementary information

Supplementary Information

Description of Additional Supplementary Files

Supplementary Data 1-30

Reporting Summary

Transparent Peer Review file

## Data Availability

Data generated and processed in this study are provided as Source Data in the form of Supplementary Data. Detailed information on the GWAS training datasets, including article references and links to the data sources, is provided in Supplementary Data 6. The unrestricted MVP summary statistics used in this study are available in the dbGaP database without additional permissions under accession code phs002453.v1.p1 [https://www.ncbi.nlm.nih.gov/projects/gap/cgi-bin/study.cgi?study_id=phs002453.v1.p1]. Accordingly, the pan-ancestry and ancestry-specific GWAS summary statistics generated in this study from the AFGen and MVP meta-analysis have been deposited in the Cardiovascular Disease Knowledge Portal database [https://cvd.hugeamp.org/downloads.html]. These data can be used to reproduce the results shown in Supplementary Figs. 26 and 27. The BBJ GWAS genotype data used in this study are available in the NBDC Human Database under accession code hum0014 [https://humandbs.dbcls.jp/en/hum0014]. The Roselli et al. all-ancestry PGS used in this study is available in the Cardiovascular Disease Knowledge Portal database [https://cvd.hugeamp.org/downloads.html#polygenic] and the PRSmix+ PGS for atrial fibrillation used in this study is available in the Polygenic Score Catalog database under accession code PGS004706. The PGS generated in this study (five ancestry-tuned Mult-t PGSs, ALLmeta PGS, and the non-EUR PGSs excluding traits trained on UK Biobank data) have been deposited in the Cardiovascular Disease Knowledge Portal database [https://cvd.hugeamp.org/downloads.html] and in the Polygenic Score Catalog under accession codes PGS012531-12538 [https://www.pgscatalog.org/]. The raw phenotypic and genetic data from the All of Us Research Program and BioBank Japan are available under restricted access for protection of individual-level data due to data privacy laws. Access to both resources can be obtained by bona fide researchers with institutional data use agreements. For All of Us, access can be obtained through the Researcher Workbench, a cloud-based computing platform [https://www.researchallofus.org/register/]. Response to access requests is typically within a few days, and continued access requires completion of an annual training course and assessment. A publicly available data browser is also provided by the program [https://databrowser.researchallofus.org/]. For BioBank Japan, access can be obtained upon request [https://biobankjp.org/english/index.html]. Both biobanks restrict use to approved research purposes, prohibit re-identification and data sharing, and require secure data handling.

## Code availability

Quality control was performed with various software tools as described in the Supplementary Information, and mostly using PLINK2 (https://www.cog-genomics.org/plink/2.0/; various versions from May 2025 release onwards). A complete, reproducible workflow for generating the multi-trait polygenic scores, including all publicly available scripts, is provided at 10.5281/zenodo.19297684. This repository^69^ contains scripts for GWAS summary statistics meta-analyses of AF and SBP using METAL (release 2011-03-25; https://csg.sph.umich.edu/abecasis/metal/download/), polygenic score generation from all-ancestry AF summary statistics using SBayesRC (v0.2.6; https://github.com/zhilizheng/SBayesRC), allele alignment between polygenic score files and All of Us genotype data, scoring of PGS files per chromosome using PLINK2 (https://www.cog-genomics.org/plink/2.0/; various versions from February 2025 release onwards), merging of per-chromosome outputs, splitting the All of Us dataset into tuning and validation subsets, integrating multiple polygenic scores using the SBayesRC-multi tool and our adapted multi tool, both run in R v4.5.0, constructing the final combined polygenic score, and finally performing downstream analyses and extracting performance metrics. Performance metrics were extracted from the logistic regression models primarily using base R. AUROC was calculated with the pROC package, AUPRC with the PRROC package, and Nagelkerke’s *R²* with the fmsb package. Liability *R²* was derived using a custom function based on formulas described in Lee et al.^73^. Analyses, data pre- and post- processing steps, and figure generation performed in R were run using versions 4.4.1–4.5.0.
