# Supplementary material for: Multi-trait polygenic risk scores improve genomic prediction of atrial fibrillation across diverse ancestries
Source: Nat Commun. 2026 May 5;17:6059. doi: 10.1038/s41467-026-72708-x (PMC13350093; doi:10.1038/s41467-026-72708-x)
Supplement: Supplementary file 1 — Supplementary Information [file 41467_2026_72708_MOESM1_ESM.pdf]

Table of contents

Table of contents 1
Supplementary Acknowledgments 3
Supplementary Note 1 3
- Performance comparisons with previous AF PGSs 3
- Technical aspects of our PGS methodology 4
- Sex-stratified and sex-optimized polygenic scores 6

Supplementary Note 2 7
- All of Us data quality control 7
- The BioBank Japan Cohort 8
  - BioBank Japan validation cohort 8
  - Genotyping, imputation, and quality control 8
- The HUNT Cohort 8
- The LOOP Cohort 9
- The UK Biobank Cohort 10

Supplementary Note 3 13
- Meta-analysis using METAL 13
  - Preparation of summary statistics for meta-analysis 13
  - Input file formatting 13
  - Data conversion and preprocessing 14
    - AFGen+MVP meta-analysis 14
    - SBP meta-analysis 14
  - Running METAL 14
  - Output processing 15
  - BMI meta-analysis (excluded due to incompatibility) 15

Supplementary Note 4 17
- PGS generation using SBayesRC 17
  - Input file formatting 17
  - Other input parameters 17
  - Data conversion and preprocessing 17
    - BMI summary statistics conversions 18
    - Missing sample size (N) column 19
    - General preprocessing for all summary statistics 19
  - Running SBayesRC 19
  - Output processing 19
    - Quality Control 20
  - Full alignment with All of Us variants 20
- Reference Maps 21
  - Reference map selection by trait 21
  - Construction of the combined reference map 21
  - Reduction in variant loss 22

Supplementary Note 5 23
- Applying the SBayesRC-multi tool (and the adapted multi tool) 23
  - Running SBayesRC-multi 23

How the SBayesRC-multi tool works 23
Adapted multi tool 24
Running the adapted multi tool 24
Supplementary Note 6 25
Figure generation and underlying analyses 25
Development of Figures that include Forest Plots 25
Decomposition of the Mult-t Scores by Trait 25
Performance comparison: The Mult-t-EUR vs Roselli et al. Score 26
Risk Stratification at the Extremes of the Polygenic Score Distribution 26
Secondary Analyses 27
Supplementary Note 7 28
Locus discovery and bias assessment of AF GWAS 28
GWAS locus definitions and gene prioritisation methods 28
Description of locus discovery in AF GWAS 29
Supplementary Figures 31
Supplementary References 48

Supplementary Acknowledgments

The BioBank Japan (BBJ) was supported by the Tailor-Made Medical Treatment Program of the Ministry of Education, Culture, Sports, Science, and Technology (MEXT) and AMED under grant numbers JP17km0305002, JP17km0305001, and JP24tm0624002.

The Trøndelag Health Study (The HUNT Study) is a collaboration between HUNT Research Center (Faculty of Medicine and Health Sciences, NTNU, Norwegian University of Science and Technology), Trøndelag County Council, Central Norway Regional Health Authority, and the Norwegian Institute of Public Health. The genotyping in HUNT was financed by the National Institutes of Health; University of Michigan; the Research Council of Norway; the Liaison Committee for Education, Research and Innovation in Central Norway; and the Joint Research Committee between St Olav's Hospital and the Faculty of Medicine and Health Sciences, NTNU.

The LOOP study was supported by The Innovation Fund Denmark [12-135225], The Research Foundation for the Capital Region of Denmark [no grant number], The Danish Heart Foundation [11-04-R83-A3363-22625], Aalborg University Talent Management Programme [no grant number], Arvid Nilssons Fond [no grant number], Skibsreder Per Henriksen, R. og Hustrus Fond [no grant number], Medtronic [no grant number], and the AFFECT-EU consortium which has received funding from the European Union's Horizon 2020 research and innovation program under grant agreement No 847770.

Supplementary Note 1

Performance comparisons with previous AF PGSs

In this study, we demonstrated that our Mult-t polygenic scores achieve superior predictive performance for AF compared to the previously established gold standard by Roselli et al. To further validate the strength of our scores, we conducted two additional comparisons with existing AF PGSs.

First, within the Roselli et al. study[1], their PGS was benchmarked against the genome-wide PGS developed by Khera et al.[2], making our Mult-t score indirectly comparable to that model. Specifically, they evaluated performance using Cox proportional hazards models for incident AF and Harrell's C-index. The Roselli et al. PGS showed improved discrimination over the Khera et al. score in both the UK Biobank (0.77 vs. 0.75) and HUNT (0.87 vs. 0.85)

cohorts[1]. Given that our Mult-t PGS outperforms the Roselli et al. score, it is likely to exceed the predictive performance of the Khera et al. PGS as well.

Second, we compared our PGSs to recent multi-ancestry scores for AF developed by Gunn et al.[3], which were constructed using the PRS-CSx[4] framework trained on MVP data and evaluated in the All of Us cohort. In that study, the reported ORs per SD (95% CI) were 1.68 (1.61–1.76) in European ancestry, 1.41 (1.25–1.60) in Admixed-American ancestry, and 1.23 (1.11–1.36) in African ancestry[3], all of which were exceeded by our Mult-t scores.

Together, these comparisons underscore the robust predictive performance of our PGSs across both European and non-European ancestry groups compared to previous AF polygenic scores.

Technical aspects of our PGS methodology

During the construction of the polygenic scores (PGSs) and subsequent analyses, we made several observations regarding key methodological aspects that likely contributed to the performance of our models.

First, we leveraged the largest available training datasets for both European and non-European ancestries, exceeding the sample sizes used in prior AF PGS studies[1,3,5,6]. This large-scale training sample size, paired with the dense genome-wide SBayesRC model, was an important contributor to the improvements over the previously published Roselli et al. score.

Second, we used all-ancestry training data rather than ancestry-specific subsets, an approach that probably owed its superiority to increased statistical power. This was exemplified by the Mult-a scores that integrated the all-ancestry (ALLmeta) and the less well performing ancestry-specific scores (e.g. EURmeta), which introduced unnecessary complexity without added predictive value compared to the ALLmeta score.

Third, when implementing the multi-trait framework, we used ancestry-specific tuning datasets rather than a single all-ancestry tuning set. This approach consistently outperformed all-ancestry tuning, likely because it better captures ancestry-specific genetic architectures. Importantly, weight estimation in the tuning step requires substantially less statistical power than genome-wide discovery, making ancestry-matched tuning feasible and beneficial. This observation aligns with prior work showing improved performance when the ancestry of tuning and validation sets is matched in penalized regression–based integration of ancestry-specific PGSs[7].

Fourth, the relative benefit of the multi-trait approach differed across ancestries, with the largest gains observed in populations where baseline prediction was poor (e.g. AMR, AFR, and SAS). Comparatively, small gains were observed in EUR and EAS where baseline prediction was already relatively strong.

One possible explanation lies in differences in linkage disequilibrium (LD) patterns across populations. Single-trait PGSs trained predominantly on specific ancestry data (e.g. EUR) may transfer poorly to other ancestries due to LD mismatches[8]. In contrast, multi-trait models may be more robust across ancestries by leveraging pleiotropic variants that are less sensitive to population-specific LD structure, and by reducing noise in effect size estimation. Consequently, the AMR, AFR and SAS populations—where LD mismatch and effect size noise are more pronounced—derive greater benefit from the multi-trait framework, whereas EUR and EAS individuals, whose genetic structure is more closely-captured by existing large-scale GWAS data, experience more limited incremental gains. Supporting this interpretation, prior work on hematological traits has demonstrated high cross-ancestry consistency of multi-trait genetic effect sizes[9], providing a biological rationale for the enhanced transferability of multi-trait models.

Further insight into the ancestry-dependent performance of the Mult-t models was obtained by decomposing the ancestry-specific mixing weights across traits. We observed substantial heterogeneity in these weights between tuning populations, indicating that trait-specific PGSs capture ancestry-dependent signals beyond those contained in the AF-only score. Notably, the contribution of a given trait within the multivariate model did not necessarily correspond to its standalone predictive performance, suggesting that the multi-trait framework prioritizes complementary information rather than maximizing marginal effects. Some discrepancies may also reflect differences between tuning and validation datasets.

Inter-ancestral differences in the estimated mixing weights likely arise from a combination of epidemiological, statistical, and data-related factors. Epidemiologically, differences in trait prevalence and gene–environment interactions across populations may lead to ancestry-specific variation in AF etiology, such that distinct biological pathways contribute differentially to disease risk. In this context, ancestry-specific mixing weights may reflect genuine differences in underlying disease mechanisms. Statistically, even when biological pathways are shared, differences in genetic architecture—including causal variants and LD structure—affect effect size estimation and PGS performance (as mentioned above). As genetic distance between discovery GWAS and target populations increases, variant effect estimates become noisier; integrating multiple correlated PGSs may therefore stabilize

prediction by capturing shared biology through partially independent genetic signals. Which traits contribute most strongly within each ancestry is then influenced by ancestry-specific genetic correlations as well as stochastic variation. Finally, bias introduced by mismatches between discovery and target populations can reduce predictive accuracy overall. In this setting, correlated traits whose GWAS data better represent the ancestry of the target population may naturally receive higher mixing weights.

Sex-stratified and sex-optimized polygenic scores

As precision medicine advances, individualized PGSs, such as those developed in this study to target specific ancestry groups, are essential for capturing the considerable genetic variability across ancestries and for avoiding the perpetuation of health disparities in underrepresented groups. To further refine risk prediction, future research should also consider sex-stratified PGSs, as the performance of AF polygenic scores may differ by sex[10].

Our subgroup analysis supported this notion, showing a stronger association between our polygenic score and AF risk in males. Additionally, epidemiological evidence indicates that men have a higher prevalence of AF, along with a greater burden of coronary artery disease and a stronger association with body mass index, whereas women with AF face higher risks of heart failure and stroke[11].

These sex-specific differences in associated traits highlight the potential value of a sex-stratified multi-trait approach to AF risk prediction. However, further investigation is warranted to determine how sex-stratified and sex-optimized PGSs may improve individualized genomic prediction.

Supplementary Note 2

All of Us data quality control

We obtained genotypes in PLINK binary format from the jointly called ACAF (an Allele Count/Allele Frequency) call set (v8) provided by All of Us (AoU). This dataset includes only high-quality genotypes, with multi-allelic variants already split into bi-allelic variants and variants with allele frequency (AF) = 0 removed, as described in the AoU Genomic Quality Report (https://support.researchallofus.org/hc/en-us/articles/29390274413716-All-of-Us-Genomic-Quality-Report). We further filtered variants based on the following criteria: (1) monomorphic variants and (2) call rate < 90%. For sample-level quality control, we excluded individuals flagged or identified as having known issues by AoU. We retained only samples with dragen_sex_ploidy equal to XX or XY, excluded samples with genotype missingness > 5%, and removed potential duplicates. Duplicate resolution prioritized retaining individuals with linked electronic health record (EHR) data, followed by higher call rate (with greater weight given to EHR availability). Potential duplicates were identified using KING v2.3.2, with one sample from each pair flagged for removal if the heterozygous concordance exceeded 0.8.

To infer population structure across all samples, we conducted principal component analysis (PCA), as the components provided by the AoU were based on external reference datasets (HGDP and 1000 Genomes Project). We first generated a filtered set of autosomal variants for both relatedness estimation and PCA. Variants were required to have a minor allele frequency (MAF) > 0.01 and genotype missingness < 1%. Linkage disequilibrium (LD) pruning was performed using PLINK with the parameters --indep-pairwise 500 200 0.1 and --indep-pairwise 2000 400 0.1. Variants located in long-range LD regions were excluded, and 100,000 variants were randomly selected from the remaining set. Based on this variant set, we estimated pairwise relatedness with KING v2.3.2, and identified unrelated individuals as those with a kinship coefficient < 0.042. PCA was then performed using flashPCA v2.0. Principal components were computed on the unrelated individuals, and the resulting components were projected onto the remaining samples.

The BioBank Japan Cohort

BioBank Japan validation cohort

We tested the performance of PRS using the BBJ 2nd cohort from the BioBank Japan Project[12]. The BBJ is a hospital-based national biobank project that collects DNA and serum samples and clinical information from 12 cooperative medical institutes throughout Japan (Osaka Medical Center for Cancer and Cardiovascular Diseases, Cancer Institute Hospital of Japanese Foundation for Cancer Research, Juntendo University, Tokyo Metropolitan Geriatric Hospital, Nippon Medical School, Nihon University School of Medicine, Iwate Medical University, Tokushukai Hospitals, Shiga University of Medical Science, Fukujuji Hospital, National Hospital Organization Osaka National Hospital, and Iizuka Hospital). BBJ 2nd cohort collected approximately 80,000 patients with 38 target diseases collected between 2013 and 2018 to expand research outcomes from the first cohort. Atrial fibrillation or atrial flutter were determined by the physician's diagnosis or electrocardiogram records. Participants provided written informed consent and the research was approved by the ethics committees of the Institute of Medical Sciences at the University of Tokyo and the RIKEN Center for Integrative Medical Sciences.

Genotyping, imputation, and quality control

All participants in BBJ 2nd cohort were genotyped using Illumina Asian Screening Array. We excluded variants meeting any of the following criteria: (1) SNP call rate <98%, (2) a minor allele count of <5 and (3) Hardy–Weinberg equilibrium $P < 1.0 \times 10^{-6}$. Post-QC genotype data were prephased using SHAPEIT2 and imputed using minimac4 with the 1 KG Phase 3 reference panel and 3,256 Japanese in-house reference panel from BBJ. Prephasing and imputation of the X chromosome were performed using the same pipeline applied for autosomes. We selected variants with Minimac4 imputation quality score of > 0.3. We excluded samples with a call rate <0.98 and a heterozygosity rate > +4 s.d. We performed principal component analysis (PCA) using PLINK 2.0 and excluded PCA outliers from the East Asian population. Finally, 64,863 individuals (5,700 cases and 59,163 controls) were included in the replication study.

The HUNT Cohort

The Trøndelag Health Study (HUNT) is a large, population-based cohort study conducted in Trøndelag County, Norway. Since 1984, the study has collected questionnaire data, clinical measurements, and biological samples from ~229,000 participants through the four surveys

HUNT1 (1984-86), HUNT2 (1995-97), HUNT3 (2006-08), and HUNT4 (2017-19)[13]. Around 88,000 participants from the last three surveys have been genotyped using Illumina HumanCoreExome arrays. This data has been further imputed using the Haplotype Reference Consortium (HRC) and TOPMed imputation panels, resulting in around 33 million well-imputed variants. Genotyping and imputation procedures in HUNT are detailed in a previous publication[14]. Sample and variant quality control were performed using standard practices described in detail elsewhere[15]. We included all genotyped participants from the HUNT4 survey; participants from earlier HUNT surveys were excluded from this analysis, to avoid overlap between base and validation data.

Data on AF status were available for all participants through linkage to national health registries covering the period from 1985 to 2024. The 9th and 10th revision of the International Statistical Classification of Diseases and Related Health Problems (ICD-9 and ICD-10) codes derived from the Nord-Trøndelag Hospital Trust were used to construct AF status. AF was defined using ICD-10 codes I48, I48.0, I48.1, I48.2, I48.3, I48.4, I48.9, and ICD-9 code 427.3.

The LOOP Cohort

Atrial Fibrillation Detected by Continuous ECG-monitoring Using Implantable Loop Recorder to Prevent Stroke in High-risk Individuals (The LOOP Study), was a randomized clinical trial, including 6004 participants without diagnosed atrial fibrillation (AF). Participants were aged 70-90 at inclusion and had CHA_2DS_2-VASc ³2. At inclusion, participants were randomized 1:3 for screening with an implantable loop recorder (ILR) or usual care[16]. ILR-detected AF was adjudicated by two consultant cardiologists. A composite endpoint of stroke and systemic embolism (SE) was adjudicated by a clinical endpoint committee.

Participants provided blood samples at inclusion and underwent genetic sequencing using the Infinium Global Screening Array v2 (Illumina), performed at the Institute of Clinical Molecular Biology (IKMB), Kiel University, Germany. Sequencing and imputation have previously been described in detail[17]. Quality control was performed at sample level by missing genotypes >2%, samples with sex mismatch, by heterozygosity rate outliers, and by filtering out related individuals and outliers in a principal component analysis. Filtering at the variant level included removal of markers with a missingness >2% and filtering at a Hardy-Weinberg equilibrium P-value $<1x10^{-10}$. Post-imputation filtering retained variants with an imputation quality of $R^2 > 0.4$.

Among sequenced participants, 5,656 (94.2%) had available genetic data and passed QC. The median age at inclusion was 73.2 years (Q1-Q3: 71.6-77.0 years). The median follow-up period was 5.4 years (Q1-Q3: 4.9-5.2 years), with no participants lost to follow-up. During follow-up, 969 (17.1%) of participants with available genetic data were diagnosed with incident AF. Incident stroke/SE occurred 206 (3.6%) of participants. The cohort was stratified by Mult-t PRS at the median, and the effects of ILR screening versus usual care was assessed in participants with PRS above and below median Mult-t PRS. Hazard ratios were estimated using Cox regressions with adjustment for sex, age, body-mass index, kidney function, alcohol intake, smoking status, plasma thyroid stimulating hormone, and clinical comorbidities at enrollment (hypertension, diabetes, prior stroke, valvular heart disease, coronary heart disease, heart failure, and peripheral artery disease. Cumulative incidences were calculated using the Aalen-Johansen estimator, considering all-cause mortality as a competing risk.

The UK Biobank Cohort

Within the UK Biobank cohort, we evaluated the Roselli et al. polygenic score and simpler versions of the non-European ancestry-optimized Mult-t polygenic scores excluding the traits height, BMI, and DCM, since these contained non-European UKB training data.

The UK Biobank is a large-scale biomedical resource comprising extensive health and genetic data from approximately 500,000 participants across the United Kingdom. Whole-genome sequencing (WGS) data were processed through the DRAGEN pipeline, with additional quality corrections applied via a machine learning-based approach. Details of the processing pipeline are available at: https://community.ukbiobank.ac.uk/hc/en-gb/articles/26273057297949-ML-Corrected-DRAGEN-whole-genome-sequencing-WGS-release. The processed data were filtered at the cohort-level and converted from DRAGEN pVCFs to PLINK2 format (PGEN, PVAR, PSAM) for further analysis (Field 24310). All analyses were conducted under UK Biobank application number 176602. The initial dataset included 1,340,689,096 autosomal variants and 490,541 individuals. We applied variant- and sample-level QC and filtering steps to the PLINK files across all autosomal chromosomes.

First, we applied variant-level quality control. Variants were excluded if they failed the DRAGEN machine-learning filters or did not meet standard INFO field criteria (INFO ≠ PASS) as assessed using PLINK2 (v- linux_avx2_20250515). Additionally, variants with missingness ≥ 0.1, minor allele count < 1, or those with ExcessHet $P < 1\times10^{-20}$ were removed. This process resulted in a final set of 1,108,365,254 autosomal variants.

We then defined a high-quality subset of autosomal variants for downstream analyses using PLINK2 (v-linux_avx2_20250515). Variants were filtered to retain those with a minor allele frequency (MAF) ≥ 0.01 and genotype missingness < 0.01. Variants located in long-range linkage disequilibrium (LD) regions were excluded. We performed LD pruning in two stages using PLINK2's --indep-pairwise command: initially with 500 200 0.1, then using 2000 400 0.1. This procedure yielded a final set of 267,759 autosomal variants, which were then used to compute sample relatedness and principal component analysis (PCA).

For sample QC, we first excluded all samples with revoked consents (N = 245). Individuals with discrepancies between genetically inferred sex versus self-reported sex were identified using X-chromosome inbreeding coefficients (F) calculated with PLINK2 (v-linux_x86_64_20240818) on pruned variants (MAF > 0.005, missingness < 0.01, pruned with --indep-pairwise 500 200 0.1 and then --indep-pairwise 2000 400 0.1). Samples with an F < 0.5 were assigned female sex and those with F > 0.8 were assigned male sex. A total of 210 samples showed sex mismatch and were removed. Duplicate pairs were identified based on a heterozygote concordance rate > 0.8, and one individual per pair was removed (N=235). Additionally, samples failing centrally computed DNA quality metrics (N=239), samples with DNA contamination score ≥ 2 (N=67), samples with missingness > 0.01 (N=0), and outliers for PC-adjusted Ti/Tv ratio, PC-adjusted Het/Hom ratio, PC-adjusted SNV/indel ratio, and PC-adjusted number of singletons (N=1653) were also excluded. After completing this process, the dataset comprised 487,894 samples.

To assess relatedness within the UK Biobank cohort, samples were split into twenty subsets. Kinship coefficients were then estimated both within and between subsets using KING (v2.3.2). We ran PCA on unrelated individuals (kinship < 0.0442) using FlashPCA (v2.0), and then projected the resulting eigenvectors onto the remaining samples.

Following QC, we conducted ancestry inference to assign individuals from the UK Biobank cohort to continental ancestry groups. We used the 1000 Genomes Project (1KG) cohort, which contains individuals with well-characterized (super)populations, as a reference for ancestry inference. Variants present in both the UK Biobank and 1KG datasets were filtered to retain those with MAF ≥ 0.01 and genotype missingness < 0.01, and excluding variants located in long-range linkage disequilibrium regions (PLINK2 v-linux_avx2_20250515). Only variants common to both cohorts were kept. LD pruning in the 1KG cohort was performed sequentially within superpopulations: starting with pruning in the African subset (AFR), then extracting those AFR-pruned variants from European (EUR) subset, before pruning EUR variants, followed by the same approach for Admixed American (AMR), South Asian (SAS), and East Asian (EAS) populations. For each pruning step, the parameters were

--indep-pairwise 1000 100 0.2. This procedure resulted in a final prune set of 98,077 variants which were extracted from the 1KG and UKB datasets. Using ADMIXTURE (v1.3.0), ancestry components were inferred from the pruned 1KG data, and these components were then projected onto UKB samples. Individuals with ≥ 0.8 estimated ancestry proportion were assigned to the corresponding continental group.

To identify individuals diagnosed with atrial fibrillation (AF) within the UK Biobank dataset, we employed the ukbrapR package (v- 0.3.7). AF cases were defined by the presence of relevant ICD-10 codes (I48, I48.0, I48.1, I48.2, I48.3, I48.4, I48.9), or ICD-9 codes (427.3, 427.31, 427.32). Participants with at least one relevant diagnostic code were classified as AF cases, using only the earliest recorded diagnosis date for each individual.

Finally, polygenic scores were calculated for all individuals who passed sample quality control, using the Roselli et al. scoring file or the simpler Mult-t scoring files for AFR and SAS populations. PGSs were evaluated only within ancestry-specific subsets, which were restricted to AFR and SAS. Case and control numbers for the AFR and SAS validation sets can be derived from Supplementary Data 16.

Supplementary Note 3

Meta-analysis using METAL

We used METAL (version released on 2011-03-25) to perform an inverse-variance weighted meta-analysis of the AFGen and MVP summary statistics for both all-ancestry and ancestry-specific datasets (EUR, AMR, and AFR). EAS summary statistics were not included, as only AFGen data was available for this ancestry.

Data pre- and post-processing were carried out on a high-performance computing cluster (HPC) using Bash shell commands (primarily `awk`), with R (v4.4.2) used when specialized functions were needed.

Preparation of summary statistics for meta-analysis

To align variants between studies, we matched variants by chromosome, position, reference allele, and alternate allele (CPRA format). This step served two purposes: (i) ensuring correct alignment of alleles across input files, and (ii) determining whether ambiguous SNPs (A/T or C/G) needed exclusion.
If the initial overlap between datasets was high (typically ~90%), we assumed correct alignment and proceeded with the original configuration. If overlap was low, we swapped the reference and alternate alleles and reassessed. In all cases, one configuration resulted in ~90% overlap and the other in ~0%, indicating strand alignment was consistent and ambiguous SNPs did not require removal. Such SNPs would have caused partial, rather than binary, overlap in both configurations. Additionally, indels were checked and excluded where necessary.

Input file formatting

For METAL input, both summary statistics files were formatted to include the following columns:

- **CPRA:** variant ID in chromosome:position:reference:alternate format
- **A1:** effect allele (uppercase)
- **A2:** non-effect allele (uppercase)
- **freq:** frequency of A1
- **b:** beta or effect size

- **se:** standard error of the effect size
- **p:** *p*-value
- **N:** total sample size per variant

For the AFGen+MVP meta-analysis, we also included **N_cases** (number of AF cases) and **N_controls** (number of controls) per variant. All input files were sorted numerically by CPRA prior to meta-analysis.

Data conversion and preprocessing

AFGen+MVP meta-analysis

- For MVP, beta values were derived by log-transforming the reported odds ratios: `beta = log(OR)`
- Standard errors were calculated from beta and *p*-value using the formula:

```
z = qnorm(p / 2, lower.tail = FALSE) * sign(beta)
SE = beta / z
```

SBP meta-analysis

- Due to the large input file sizes (28M and 96M SNPs), a minor allele frequency (MAF) filter >0.0025 was applied to reduce runtime.
- For the Shi dataset, $-\log_{10}(p)$ values were converted to *p*-values, and variants with INFO score <0.8 were excluded.
- Shi reported a rank-based inverse normal transformation, while MVP applied a standard inverse normal transformation. Compatibility was confirmed by comparing effect sizes of the top SNPs (lowest *p*-values) and their beta distributions after filtering on MAF >0.05. These were comparable, allowing us to proceed without further transformation.

Both analyses included checks for missing values (NA), and variants with missing data were removed.

Running METAL

Meta-analyses were executed using METAL. The execution script for both the AFGen+MVP[18] and SBP[19] datasets is available in the associated GitHub repositories.

Output processing

Following meta-analysis, output files were sorted by CPRA. For the SBP meta-analysis, sample size values in scientific notation were converted to plain numbers using `awk` with the `sprintf` function.

METAL output included all input columns, along with:

- **FreqSE** (standard error of allele frequency)
- **Direction** (effect direction per study, e.g., ++ for concordant effects)

Since METAL does not output rsIDs when using CPRA format, we added a **SNP** column containing rsIDs, which was required for SBayesRC:

- For the AFGen+MVP meta-analysis output, rsIDs (hg38) were retrieved from the original summary statistics by matching on chromosome and position (hg38).
- For the SBP meta-analysis output, rsIDs (hg38) were retrieved from the AFGen+UKB reference map by matching on chromosome and position (hg38), as the original Shi dataset lacked rsIDs. Details on this map are provided in the *Reference Map* section.

BMI meta-analysis (excluded due to incompatibility)

We explored a meta-analysis of BMI summary statistics from Jonsdottir and MVP to increase the sample size with diverse ancestries. MVP included 424,221 EUR, 57,793 AMR, 118,993 AFR, and 6,384 EAS individuals (GCST90479521). Jonsdottir reported raw BMI, whereas MVP applied an inverse normal transformation.
To approximate a raw BMI scale for MVP, we multiplied beta values by 5.5, reflecting the standard deviation (SD) observed in a cohort of 6,242 Mexican American adults (mean BMI: 28.0 kg/m², SD: 5.5 kg/m²)[20]. We assessed compatibility before and after this transformation by comparing the effect sizes of the 10–20 most significant SNPs (lowest *p*-values) and the overall beta distributions after filtering on MAF >0.05. In both cases, comparability remained limited. Additionally, when running SBayesRC with this input, the MCMC iterations yielded unrealistically high SNP heritability estimates (around $h^2 = 0.7$).
These findings suggested scale incompatibility between the datasets. Therefore, we

excluded the MVP data and proceeded with the Jonsdottir data alone for downstream analyses.

Supplementary Note 4

PGS generation using SBayesRC

Input file formatting

For SBayesRC (v0.2.6) input, summary statistics files were formatted to include the following columns:

- **SNP:** variant ID in rsID format
- **A1:** effect allele (uppercase)
- **A2:** non-effect allele (uppercase)
- **freq:** frequency of A1
- **b:** beta or effect size
- **se:** standard error of the effect size
- **p:** *p*-value
- **N:** total sample size per variant

Other input parameters

- **LD reference panel**: Linkage disequilibrium (LD) reference panels for three ancestries were available, based on imputed genotypes from the UK Biobank (UKB): European (EUR, n = 347,800), African (AFR, n = 7,006), and East Asian (EAS, n = 2,252). We used ancestry-matched LD reference panels for the EUR, AFR, and EAS summary statistics. For summary statistics resulting in the AMRmeta, ALLmeta, or trait-specific PGSs, the European LD reference was used due to the absence of AMR- and all-ancestry LD panels.
- **Annotation file**: Functional annotations for the 8,140,664 UKB imputed SNPs (Baseline-LD model v2.2[21]) were used.

Both the LD reference files and annotation file were downloaded from the Zhili Zheng GitHub repository[22].

Data conversion and preprocessing

The HF summary statistics required no conversion, as all required columns were present. The same applied to the SBP, as well as the ancestry-specific (EUR, AMR,

AFR) and all-ancestry AF summary statistics derived from METAL, which outputs the columns needed for SBayesRC. An rsID column was added afterward; see the *METAL output processing* section for details.

Data pre- and post-processing were carried out on a HPC using Bash shell commands (primarily `awk`), with R (v4.4.2) used when specialized functions were needed.

SBayesRC requires variant identifiers in rsID format. Therefore we converted the summary statistics that lacked this column:

- **Height, CAD, and PR interval:** rsIDs (hg38) were retrieved from the AFGen+UKB reference map by matching on chromosome and position (hg19).
- **BMI:** rsIDs (hg38) were retrieved from the AFGen+UKB reference map by matching on chromosome and position (hg38).
- **DCM:** rsIDs (hg38) were retrieved from a separate UKB reference map by matching on chromosome and position (hg19).

Details on these reference maps are provided in the *Reference Map* section.

BMI summary statistics conversions

- In the CPRA column, we removed the "chr" prefix and converted the delimiter from "_" to ":".
- We added a freq1 column by matching on CPRA format and A1 allele from the all-ancestry AFGen+MVP summary statistics. If A2 in the BMI data matched the A1 in the reference, alleles were flipped and the beta value multiplied by -1.
- Variants were filtered on MAF >0.0025, as the file contained ~27 million SNPs after frequency imputation and was initially prepared for METAL input. This filter was retained in subsequent steps. This was considered acceptable, as the final BMI scoring file contained a number of SNPs comparable to those of the other traits.

- Outlier filtering: variants with extreme beta values (>1 SD from the median; -0.845 < b > 0.845) and variants with implausibly high sample sizes (N > 2,005,180; the total sample size for BMI) were removed.
- Standard errors were calculated from beta and p-value using the formula:

```
z = qnorm(p / 2, lower.tail = FALSE) * sign(beta)
SE = beta / z
```

Missing sample size (N) column

For BMI and PR interval, we calculated effective sample sizes (Neff) from MAF and SE using:

```
MAF = ifelse(freq > 0.5, 1 - freq, freq)
Neff = 1 / (2 * MAF * (1 - MAF) * (SE ^ 2))
```

General preprocessing for all summary statistics

- Duplicate rsIDs from input files were removed unless they had identical alleles and effect sizes or represented multi-allelic variants.
- Removed variants with non-ACGT alleles.
- Removed Indels.
- Excluded variants with missing values (NA).

Running SBayesRC

We used Apptainer (formerly Singularity) to run SBayesRC within a pre-built containerized environment provided by the tool's developer, which included all required packages and dependencies. SBayesRC was run with 16 CPUs and ~70 GB of memory, with a typical runtime of approximately 4.5 hours.
An example execution script for the all-ancestry AFGen+MVP summary statistics is available in the associated GitHub repository[23].

Output processing

Since SBayesRC does not output CPRA format, this was added manually, as it was required for downstream analyses in All of Us. We distinguished between input SNPs, present in the original summary statistics, and imputed SNPs, added by SBayesRC due to presence in the annotation file but absence in the input:

- For AF ancestry-specific and all-ancestry summary statistics, CPRA format (hg38) for input SNPs was retrieved from the original summary statistics by matching on rsIDs (hg38). For imputed SNPs, rsIDs from the UKB reference map were used for matching.
- For other trait-specific summary statistics (excluding DCM), CPRA format (hg38) for both input and imputed SNPs was retrieved from the AFGen+UKB reference map by matching on rsIDs (hg38). For DCM, the UKB reference map alone was sufficient, as no variant loss was observed. The original summary statistics were not used here, as most lacked rsIDs.
- Thereafter, for trait-specific summary statistics (excluding AF), A1 and A2 alleles from the original summary statistics were re-added into the CPRA column in alignment with the reference map. This ensured allele consistency with the original summary statistics. For AF, this step was not necessary, as CPRA format was derived directly from the original summary statistics.

Quality Control

- Duplicate rsIDs present in the (AFGen+)UKBrefmap were absent in the files generated after adding CPRA format to the SBayesRC output by matching on rsIDs. Duplicates in these files could have led to incorrect CPRA assignments.
- No duplicate rsIDs or CPRA identifiers were found in the final scoring files.
- Indels were removed from the final scoring files if necessary.
- Checked CPRA overlap with the (AFGen+)UKB reference map and aligned reference and alternate alleles accordingly, as this map was harmonized as much as possible with the All of Us variant set.

Full alignment with All of Us variants

To minimize variant loss when applying the final scoring files in All of Us, we first concatenated all `.pvar` chromosome files to generate a merged reference file containing all variants present in All of Us participants with available short-read whole genome sequencing (srWGS) data. We then assessed overlap between this reference and the final PGS file by matching variants using the CPRA format. All steps were performed using Bash shell commands on the All of Us cloud computing platform.

Variants that did not match were separated, and their reference and alternate alleles were flipped. These flipped variants were appended to the bottom of the PGS file. A bash shell script incorporating these steps has been made available[24]. The resulting file was fully aligned with the All of Us variant set and ready for polygenic scoring using the `--score` flag in PLINK2.

Reference Maps

We constructed reference maps to facilitate several essential data conversions:

- Converting CPRA format to rsIDs following METAL output.
- Converting rsIDs back to CPRA format after SBayesRC output.
- Lifting over summary statistics from genome build GRCh37 (hg19) to GRCh38 (hg38).

Each reference map included the following columns: CPRA (hg19), CPRA (hg38), and rsID (hg38). Reference and alternate alleles were aligned to maximize concordance with allele representations in the All of Us dataset.

Reference map selection by trait

The combined AFGen+UKB reference map was used for all traits except AF and DCM, for which the UKB reference map alone was sufficient. For these two traits, variant-level missingness was minimal or nonexistent, either when recovering CPRA format from imputed SNPs from the SBayesRC output (AF) or during build conversion from GRCh37 to GRCh38 (DCM). In contrast, applying the UKB reference map to other traits led to substantial missingness during build conversion: CAD (33.6%), Height (7.1%), and PR-interval (4.0%). This motivated the construction of the AFGen+UKB reference map, which was applied to the remaining traits.

Construction of the combined reference map

To build the combined reference map, we merged reference files derived from the AFGen summary statistics and the UK Biobank. We retained only the unique rows

and appended AFGen entries below those of UKB. This was done on a HPC using Bash shell commands.

Reduction in variant loss

Using the AFGen+UKB reference map reduced variant loss during genome build liftover. For example, from 33.6% to 23.0% for CAD. Although some variants were still lost, the number included in the final scoring files was comparable across traits, even in traits with minimal loss. Therefore, we considered the persisting gap acceptable.

Supplementary Note 5

Applying the SBayesRC-multi tool (and the adapted multi tool)

We applied the SBayesRC-multi function by tweaking the publicly available code from Zhili Zheng's GitHub repository[22]. Several adjustments were made to tailor the script to our data and study design:

- Renamed the score column from `SCORE` to `SCORE1_SUM` to match our input files.
- Replaced linear regression with logistic regression to accommodate the binary outcome (AF presence vs. absence).
- Substituted adjusted R^2 with Nagelkerke's R^2 for model evaluation in the tuning set.
- Modified the script to read CSV input files instead of TSV.

Running SBayesRC-multi

We ran the tool in RStudio (v4.5.0) on the All of Us cloud computing platform. The execution script is available in the associated GitHub repository[25].

How the SBayesRC-multi tool works

The SBayesRC-multi tool takes as input a tuning set file, two PGS files, and a phenotype file. It first merges these datasets to retain only individuals present in the tuning set, with columns for each PGS and the phenotype (AF case = 1, control = 0). A logistic regression model is then fitted in the tuning set, using AF status as the outcome and both PGSs as predictors. This yields an intercept and a regression coefficient (weight) for each PGS, so that the scores are mutually adjusted and weighted according to their predictive value in the tuning set. PGSs with higher predictive accuracy receive higher weights.

Using the fitted model and the `predict()` function in R, the tool computes a new, weighted PGS for each individual in the full dataset. To prevent overfitting, individuals from the tuning set were excluded from downstream evaluation; this step was performed outside the tool. The tool outputs (i) the unscaled regression coefficients for each PGS, (ii) Nagelkerke's R^2 in the tuning set for each input PGS and the weighted PGS, and (iii) the final weighted PGS values for each individual.

Adapted multi tool

We later developed an adapted version of the SBayesRC-multi tool to improve flexibility in both input and output handling. The key differences compared to the SBayesRC-multi implementation are:

- Support for more than two input PGS files, allowing integration of multiple scores simultaneously.
- Two output files:
    1. **`.unscaled.regcoef.csv`**, containing:
        - Unscaled regression coefficients, based on the raw PGS inputs (i.e., without normalization or standardization).
        - Proportions: the coefficients normalized to sum to 100% using `abs(regrcoef) / sum(abs(regrcoef)) * 100`
        - Nagelkerke's R^2.
    2. **`.mixing.weights.csv`**, containing:
        - Scaled regression coefficients (mixing weights), calculated by multiplying each unscaled coefficient by the standard deviation of its corresponding PGS in the tuning set.
        - Proportions
        - Standard deviations

This replaces the original **`.weight`** file, which included only the unscaled regression coefficients and Nagelkerke's R^2.

Running the adapted multi tool

The execution script is available in the associated GitHub repository[26].

Supplementary Note 6

Figure generation and underlying analyses

The following analyses were performed in RStudio (v4.5.0) on the All of Us cloud computing platform. Figure generation was done in RStudio (≥ v4.4.1), either on the cloud or locally. However, flowcharts (Figure 1, and Supplementary Figures 1, 3, 4, and 6) were created using BioRender.com.

Development of Figures that include Forest Plots

Figures displaying forest plots were created using the `forest()` function from the metafor package. Each figure shows a forest plot (left) and additional performance metrics in a Table-like layout (right), both generated using a single call to `metafor::forest()`.

Most performance metrics were extracted using base R, while AUROC was calculated with the pROC package, AUPRC with the PRROC package, and Nagelkerke's R^2 with the fmsb package. Liability R^2 was derived using a custom function based on the formulas from this study[27].

Decomposition of the Mult-t Scores by Trait

The figure showing the scaled relative regression coefficients (mixing weights) of the Mult-t PGSs across ancestry-specific tuning sets (Figure 4) was based on the mixing proportions per trait from the `.mixing.weights.csv` output file (Supplementary Data 10) of the adapted multi tool (Supplementary Figure 3). The figure showing the unscaled relative regression coefficients (Supplementary Figure 9) used the proportions from the `.unscaled.regcoef.csv` file (Supplementary Data 11). The derivation of these proportions is described in the Adapted multi tool section. Both bar plots were generated using `ggplot()` and `geom_bar()` from the ggplot2 package, with styling from `theme_cowplot()` in the cowplot package.

Trait-specific PGSs evaluated individually (Supplementary Figures 10–14; Supplementary Data 12) were visualized using forest plots created with

`metafor::forest()` from the metafor package, following the same approach as in the main results.

Performance comparison: The Mult-t-EUR vs Roselli et al. Score

Both the Roselli et al. and Mult-t-EUR polygenic scores were first normalized by adjusting for the first 20 principal components (PCs) of ancestry, then standardized to a mean of 0 and a standard deviation of 1. Subsequently, the PGSs were divided into 100 percentile groups using `percent_rank()` from the dplyr package.

To create the figure showing AF prevalence with 95% confidence intervals (**Figure 6**), we calculated the prevalence per percentile group as the proportion of cases `(cases / N * 100)`. The 95% confidence intervals were estimated using `binom.test()`, and bounds were multiplied by 100 to report percentages. The graph was created using `ggplot()`, with `geom_point()` for the prevalence dots, `geom_errorbar` for the confidence intervals, and `geom_hline` for the horizontal dashed lines displaying the prevalence values at the lowest and highest percentile.

For the figure showing the distribution of cases and controls across percentiles (Supplementary Figure 16), we created separate data frames for cases and controls, stratified by PGS percentile. These were combined and labeled, and plotted using `geom_boxplot()`, which internally calculates and displays the median, interquartile range, and whiskers. To report exact summary values for cases, we also calculated the median and interquartile range using `median()`, and `quantile()` with 0.25 and 0.75 as input values.

Risk Stratification at the Extremes of the Polygenic Score Distribution

To identify individuals with a 3-, 4-, or 5-fold increased risk (Figure 7 and Supplementary Figure 18) or a ⅓-fold decreased risk (Supplementary Figure 17 and 19) of AF compared to the middle quintile, we sorted individuals by their normalized and standardized PGS values from high to low. This was done separately for both the Roselli et al. and Mult-t scores. For each risk threshold, we iteratively defined the top group as individuals with PGS values above a moving threshold. Starting from the highest PGS value, we progressively included more individuals by lowering this threshold in fixed steps. At each step, the top group was compared to the middle

quintile group (defined using the `quantile()` function at the 40th and 60th percentiles) using logistic regression adjusted for age and sex. The process continued until the smallest group with an odds ratio (OR) ≥3, 4, or 5 was identified. The corresponding PGS threshold and the proportion of individuals within each group were stored. For the decreased risk analysis (⅓-fold), we initiated the search at the 40th percentile and expanded downward to identify the largest group with OR ≤ ⅓ compared to the middle quintile. Groups identified with an even lower risk (e.g., <¼- or ⅕-fold) were too small for reliable interpretation and were therefore excluded.

Density plots were generated using ggplot2, with vertical dashed lines marking the PGS thresholds for each risk group added via `geom_vline()`. Regions corresponding to >3-, 4-, or 5-fold increased risk (or <⅓-fold decreased risk) were shaded in progressively darker reds using `geom_area()`, while the middle quintile region (40th–60th percentile) was similarly shaded in grey with `geom_area()` and bounded by vertical dashed lines.

Histograms displaying the proportion of individuals within the increased risk groups (Figure 7 and Supplementary Figure 18) or the decreased risk group (Supplementary Figure 17 and 19) were created using `ggplot()` and `geom_col()`, with styling from `theme_cowplot()` in the cowplot package.

Secondary Analyses

Figures for the sensitivity (Supplementary Figure 24) and subset analyses (Supplementary Figure 25) were generated using `metafor::forest()` from the metafor package. Exclusion of individuals from Massachusetts or affiliated with the Veteran Affairs system (for the sensitivity analysis), as well as subset extraction for the Mult-t-EUR PGS, was performed after PGS normalization and standardization, and following ancestry-specific subset selection, but prior to fitting the logistic regression model.

Supplementary Note 7

Locus discovery and bias assessment of AF GWAS

GWAS locus definitions and gene prioritisation methods

To comprehensively characterise genome-wide significant signals and prioritise candidate genes, we applied two complementary approaches for locus definition and gene assignment:

1. a distance-based nearest-gene approach using GWAS summary statistics, and
2. a fine-mapping– and machine-learning-informed gene prioritisation approach.

For the distance-based approach, genome-wide significant loci were defined from AFib GWAS summary statistics using the `get_loci()` function from the gwasRtools R package. Variants were first filtered to retain those with valid chromosome, position, *P*-value, and allele frequency information. Genome-wide significant variants ($P < 5 \times 10^{-8}$) were identified, and for each variant a ±500 kb window (1 Mb total) was constructed. Overlapping windows were merged to define independent GWAS loci. Within each locus, the lead SNP was defined as the variant with the smallest *p*-value.

For each lead SNP, the nearest gene was annotated based on physical distance to the gene body using `get_nearest_gene()` (GRCh37 build). This approach provides a simple and interpretable baseline gene assignment based solely on genomic proximity to the association signal.

For machine-learning-based gene prioritization, in parallel, we applied FLAMES, a fine-mapping–based gene prioritisation framework that integrates statistical fine-mapping results with gene-level aggregation[28]. FLAMES leverages posterior inclusion probabilities derived from SuSiE fine-mapping to quantify the contribution of variants within a locus and assigns gene-level scores by aggregating evidence across variants mapped to each gene. This approach prioritises genes based on the overall fine-mapping signal rather than reliance on a single lead variant.

Fine-mapping and FLAMES analyses were performed using linkage disequilibrium estimated from the UK Biobank European (EUR) reference panel, providing a consistent LD structure for probabilistic inference across loci.

To assess concordance between GWAS-defined loci and FLAMES fine-mapping results, we implemented a positional overlap strategy. For each GWAS locus defined by `get_loci()`, a ±500 kb window was constructed around the lead SNP. FLAMES fine-mapped variants from credible sets with FLAMES score > 0 and *P* < 5e-8 were then considered overlapping with lead `get_loci()` variants if they fell within this window of 500 kb base-pairs on the same chromosome. For loci with multiple overlapping FLAMES variants, a single representative hit was selected based on the highest SuSiE posterior inclusion probability, with ties further prioritised by higher FLAMES score and lower GWAS *P*-value. Loci without any FLAMES variants in the ±500 kb window were retained and marked as non-overlapping.

This dual strategy allowed us to contrast simple proximity-based gene assignment with a more sophisticated fine-mapping–informed approach, and to identify loci where gene prioritisation is sensitive to LD reference structure and fine-mapping resolution. The summary of this approach is provided in Supplementary Data 30.

Description of locus discovery in AF GWAS

The distance-based approach led to the discovery of 486 distinct loci in our AF GWAS meta-analysis (Supplementary Figure 26 and 27). FLAMES identified 1029 credible sets that (i) contain at least one genome-wide significant variant and (ii) that could be mapped to a machine-learning prioritized gene (FLAMES_score > 0). Of the 486 distinct loci based on distance, 416 loci (85.6%) overlapped with such a credible-set with a FLAMES-prioritized gene (Supplementary Data 30). Of note: (i) several credible sets may map to a single locus - explaining why 1029 credible sets become 416 loci - and (ii) some loci cannot be reliably fine-mapped (due to mismatches between LD reference and our multi-ancestry GWAS data) and therefore did not undergo FLAMES prioritization.

Compared to a recent AF GWAS with comparable case numbers[29], we identified fewer loci: Yuan et al. analyzed 252,438 AF cases and identified 525 significant loci.

However, we note that Yuan et al. reported a substantial LD score regression intercept of 1.34 (with an attenuation ratio of 15%). In contrast, using LD score regression and using a European LD reference, we estimate an LD score intercept of our GWAS of 1.11 (SE=0.018; attenuation ratio 7.3%). Therefore, our GWAS data seem to contain 2-3-fold less bias, potentially explaining some difference in locus discovery and hinting at better locus specificity in our GWAS as compared to Yuan et al.

Supplementary Figures

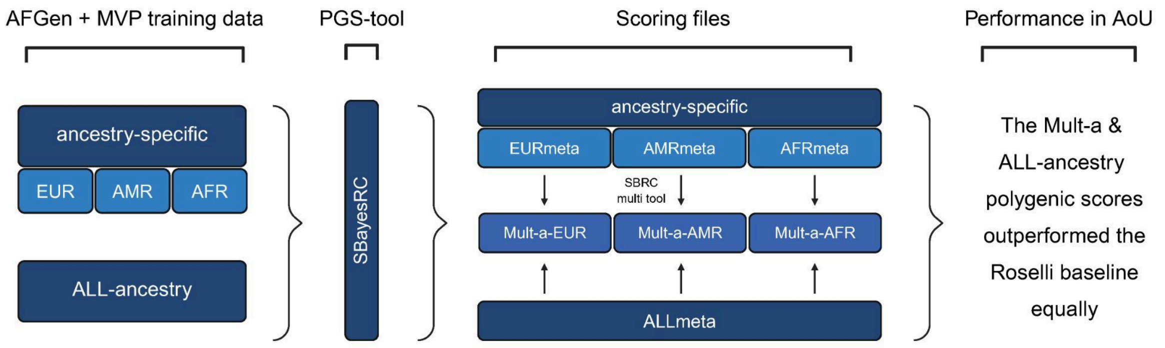

Supplementary Figure 1 | Mult-a polygenic scores development, an overview. This flowchart outlines the development of the Mult-a polygenic scores from left to right. Ancestry-specific (EUR, AMR, AFR) and all-ancestry GWAS summary statistics from the AFGen and MVP cohorts were first processed using SBayesRC to generate scoring files. We then used the SBayesRC-multi tool to combine each ancestry-specific file with the all-ancestry file, weighted by predictive accuracy in the 30% tuning dataset of All of Us, to create ancestry-matched Mult-a scores. In the final step, the scoring files were evaluated in the 70% validation dataset of All of Us (Supplementary Data 1), where both the Mult-a scores and the all-ancestry (ALLmeta) score performed equally well and outperformed the all-ancestry trained Roselli baseline. The Mult-a-EAS and Mult-a-SAS scores were not constructed because ancestry-specific tuning would have resulted in insufficient sample sizes for validation. Created in BioRender. Lahrouchi, N. (2026) https://BioRender.com/4tz6zwj.

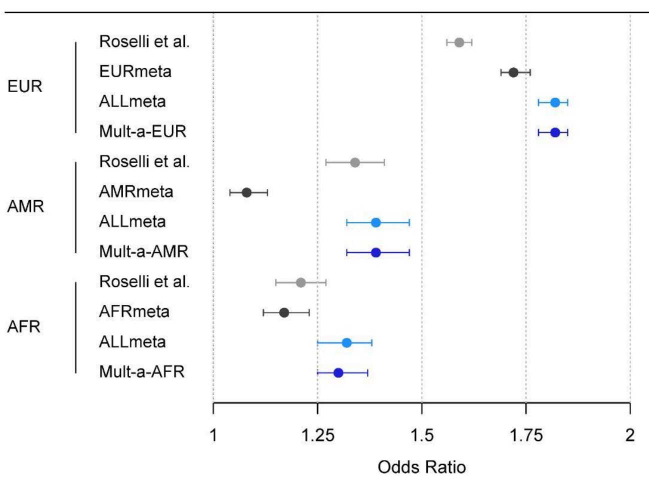

Cohort	PGS	OR [95% CI]	*P*-value	AUROC	AUPRC	Ngk-R^2	Liab-R^2
EUR	Roselli et al.	1.59 [1.56, 1.62]	<1.00E-300	0.613	0.137	0.040	0.059
	EURmeta	1.72 [1.69, 1.76]	<1.00E-300	0.631	0.150	0.058	0.091
	ALLmeta	1.82 [1.78, 1.85]	<1.00E-300	0.642	0.155	0.065	0.098
	Mult-a-EUR	1.82 [1.78, 1.85]	<1.00E-300	0.644	0.158	0.068	0.103
AMR	Roselli et al.	1.34 [1.27, 1.41]	6.73E-28	0.578	0.050	0.012	0.024
	AMRmeta	1.08 [1.04, 1.13]	7.98E-05	0.525	0.040	0.002	0.003
	ALLmeta	1.39 [1.32, 1.47]	2.84E-37	0.591	0.053	0.017	0.032
	Mult-a-AMR	1.39 [1.32, 1.47]	2.84E-37	0.591	0.053	0.017	0.032
AFR	Roselli et al.	1.21 [1.15, 1.27]	1.41E-14	0.548	0.056	0.004	0.008
	AFRmeta	1.17 [1.12, 1.23]	3.14E-12	0.541	0.056	0.004	0.007
	ALLmeta	1.32 [1.25, 1.38]	3.67E-30	0.566	0.061	0.010	0.018
	Mult-a-AFR	1.30 [1.25, 1.37]	5.84E-30	0.567	0.060	0.010	0.018

Supplementary Figure 2 | Comparison of the Roselli et al., ancestry-specific, ALLmeta, and Mult-a polygenic scores. The left panel displays a forest plot of the OR/SD increase in PGS for AF, with 95% CIs on the x-axis. The y-axis lists ancestry-specific cohorts from the All of Us 70% validation dataset (Supplementary Data 1): EUR (n=124,367 of which 11,087 cases), AMR (n=40,219 of which 1,449 cases), and AFR (n=44,692 of which 2,112 cases), each paired with its PGSs. The right panel summarizes key performance metrics. All estimates are based on logistic regression models adjusted for the first 20 PCs, age, and sex, except for AUROC and AUPRC values which are based on univariate models (to show prediction from PGS alone). *P*-values were calculated using two-sided Wald tests. The Mult-a-EAS and Mult-a-SAS scores were not evaluated because ancestry-specific tuning would have resulted in insufficient sample sizes for validation. Source Data are provided (Supplementary Data 2).

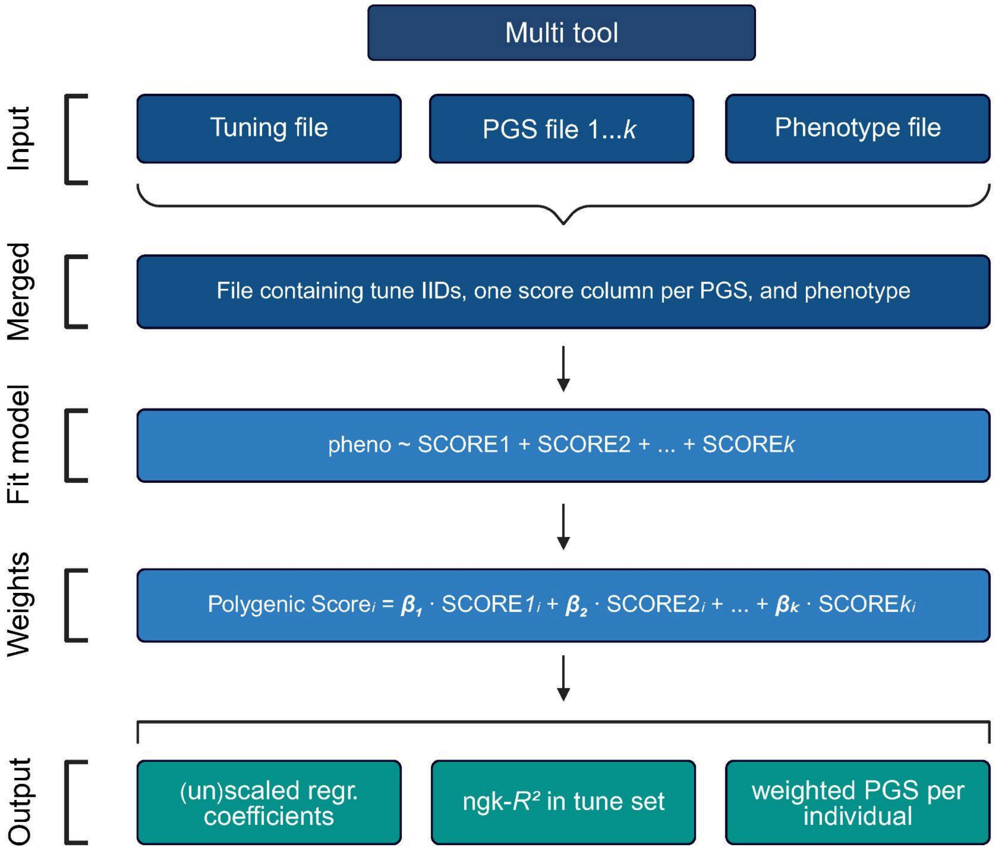

Supplementary Figure 3 | Workflow of the adapted multi tool. The adapted tool accepts a tuning file, multiple PGS files, and a phenotype file, merges these data, and fits a logistic regression model using AF status as the outcome and the PGS inputs as predictors. It outputs (i) unscaled regression coefficients based on the raw PGS inputs (i.e., without normalization or standardization), (ii) the scaled coefficients, or mixing weights, obtained by multiplying each unscaled coefficient by the standard deviation (SD) of its corresponding PGS in the ancestry-specific tuning set, (iii) Nagelkerke's R^2 for each input PGS and the weighted score, and (iv) the final weighted PGS per individual. Created in BioRender. Lahrouchi, N. (2026) https://BioRender.com/4tz6zwj.

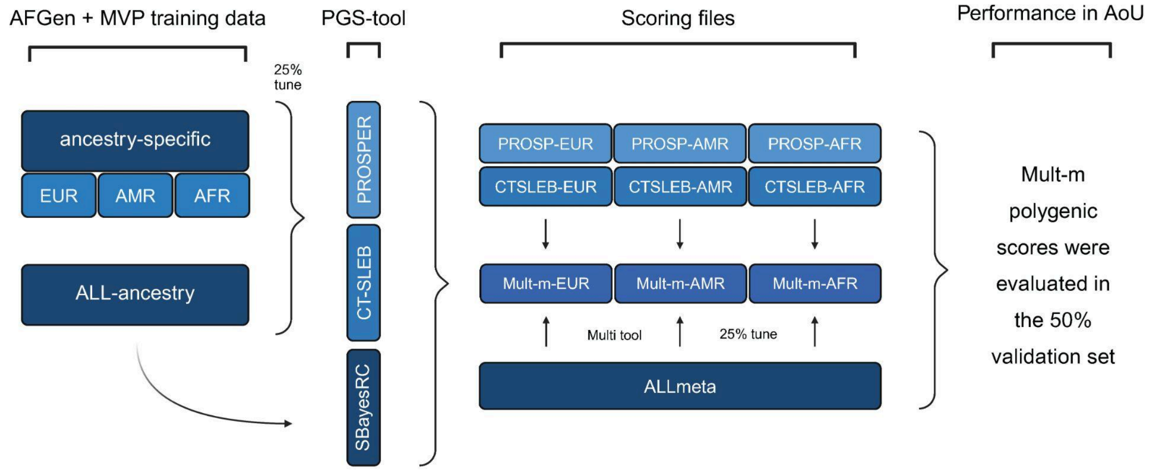

Supplementary Figure 4 | Mult-m polygenic scores development, an overview. This flowchart outlines the development of the Mult-m polygenic scores from left to right. Ancestry-specific (EUR, AMR, AFR) and all-ancestry GWAS summary statistics from the AFGen and MVP cohorts were combined in the first 25% tuning set of All of Us (AoU) using the PGS tools PROSPER or CT-SLEB to generate scoring files. Additionally, only the all-ancestry GWAS summary statistics were processed with SBayesRC to generate the ALLmeta scoring file. In the second 25% tuning set of AoU, we used the adapted multi tool to combine each ancestry-specific PROSPER or CT-SLEB scoring file with the SBayesRC (ALLmeta) scoring file, yielding multi method (Mult-m) scores. The resulting Mult-m scores were evaluated in the remaining 50% validation dataset of AoU. Created in BioRender. Lahrouchi, N. (2026) https://BioRender.com/4tz6zwj.

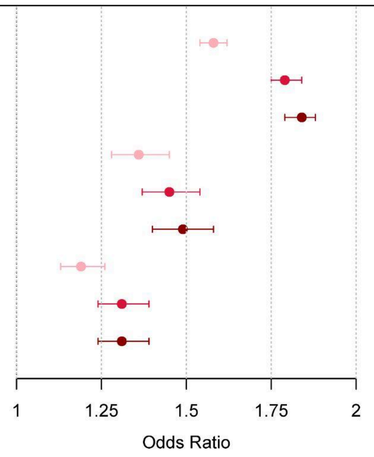

Cohort	PGS	OR [95% CI]	*P*-value	AUROC	AUPRC	Ngk-R^2	Liab-R^2
EUR	Roselli et al.	1.58 [1.54, 1.62]	1.13E-295	0.612	0.136	0.039	0.058
	ALLmeta	1.79 [1.75, 1.84]	<1.00E-300	0.639	0.152	0.062	0.095
	Mult-m-EUR	1.84 [1.79, 1.88]	<1.00E-300	0.645	0.158	0.069	0.108
AMR	Roselli et al.	1.36 [1.28, 1.45]	4.35E-23	0.580	0.053	0.014	0.027
	ALLmeta	1.45 [1.37, 1.54]	7.73E-34	0.599	0.056	0.021	0.039
	Mult-m-AMR	1.49 [1.40, 1.58]	1.53E-36	0.602	0.058	0.024	0.046
AFR	Roselli et al.	1.19 [1.13, 1.26]	1.19E-09	0.544	0.055	0.004	0.007
	ALLmeta	1.31 [1.24, 1.39]	5.54E-22	0.567	0.061	0.010	0.017
	Mult-m-AFR	1.31 [1.24, 1.39]	2.58E-21	0.569	0.061	0.010	0.016

Supplementary Figure 5 | Comparison of the Roselli et al., ALLmeta, and Mult-m polygenic scores. The left panel displays a forest plot of the OR/SD increase in PGS for AF, with 95% CIs on the x-axis. The y-axis lists ancestry-specific cohorts from the All of Us 50% validation dataset: EUR (n=88,875 of which 7,834 cases), AMR (n=28,704 of which 1,032 cases), and AFR (n=31,796 of which 1,504 cases), each paired with its PGSs. The right panel summarizes key performance metrics. All estimates are based on logistic regression models adjusted for the first 20 PCs, age, and sex, except for AUROC and AUPRC values which are based on univariate models (to show prediction from PGS alone). *P*-values were calculated using two-sided Wald tests. Source Data are provided (Supplementary Data 3).

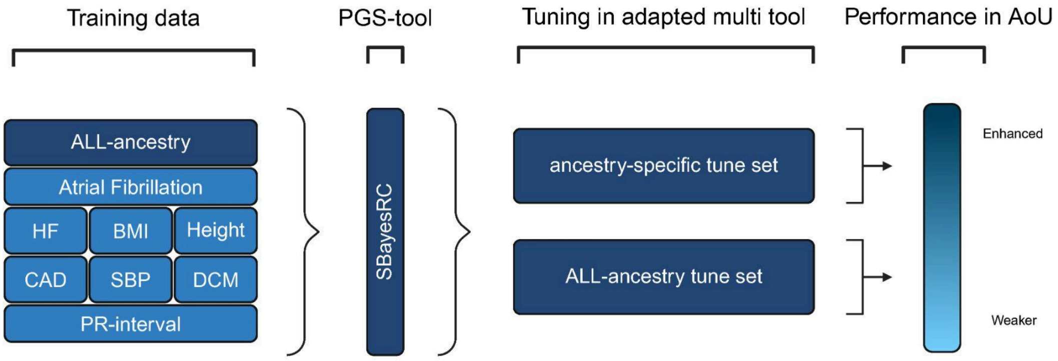

Supplementary Figure 6 | Ancestry-specific tuning sets enhance Mult-t score performance over all-ancestry tuning. This flowchart outlines the development of the Mult-t polygenic scores from left to right. All-ancestry GWAS summary statistics for AF and seven correlated traits were first processed using SBayesRC to generate trait-specific scoring files. We then used the adapted multi tool to combine these scoring files, weighted by predictive accuracy in either an ancestry-specific or all-ancestry 30% tuning dataset of All of Us, resulting in the ancestry-tuned and all-tuned Mult-t scores, respectively. In the final step, scores were evaluated in the 70% validation dataset of All of Us (Supplementary Data 1). Created in BioRender. Lahrouchi, N. (2026) https://BioRender.com/4tz6zwj.

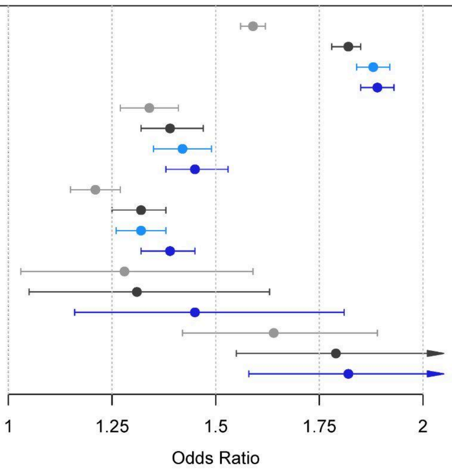

Cohort	PGS	OR [95% CI]	*P*-value	AUROC	AUPRC	Ngk-R^2	Liab-R^2
EUR	Roselli et al.	1.59 [1.56, 1.62]	<1.00E-300	0.613	0.137	0.040	0.059
	ALLmeta	1.82 [1.78, 1.85]	<1.00E-300	0.642	0.155	0.065	0.098
	Mult-t-ALL	1.88 [1.84, 1.92]	<1.00E-300	0.644	0.156	0.069	0.107
	Mult-t-EUR	1.89 [1.85, 1.93]	<1.00E-300	0.646	0.157	0.071	0.110
AMR	Roselli et al.	1.34 [1.27, 1.41]	6.98E-28	0.578	0.050	0.012	0.024
	ALLmeta	1.39 [1.32, 1.47]	2.93E-37	0.591	0.053	0.017	0.032
	Mult-t-ALL	1.42 [1.35, 1.49]	9.59E-41	0.594	0.054	0.019	0.037
	Mult-t-AMR	1.45 [1.38, 1.53]	7.11E-45	0.595	0.054	0.021	0.043
AFR	Roselli et al.	1.21 [1.15, 1.27]	1.43E-14	0.548	0.056	0.004	0.008
	ALLmeta	1.32 [1.25, 1.38]	3.76E-30	0.566	0.061	0.010	0.018
	Mult-t-ALL	1.32 [1.26, 1.38]	1.16E-32	0.566	0.061	0.011	0.020
	Mult-t-AFR	1.39 [1.32, 1.45]	6.42E-43	0.573	0.064	0.014	0.028
SAS	Roselli et al.	1.28 [1.03, 1.59]	2.42E-02	0.583	0.030	0.008	0.015
	ALLmeta	1.31 [1.05, 1.63]	1.73E-02	0.579	0.030	0.009	0.020
	Mult-t-SAS	1.45 [1.16, 1.81]	1.29E-03	0.600	0.033	0.016	0.037
EAS	Roselli et al.	1.64 [1.42, 1.89]	1.9E-11	0.624	0.052	0.034	0.061
	ALLmeta	1.79 [1.55, 2.07]	2.67E-15	0.657	0.055	0.047	0.075
	Mult-t-EAS	1.82 [1.58, 2.11]	4.55E-16	0.659	0.061	0.050	0.079

Supplementary Figure 7 | Comparison of the Roselli et al., ALLmeta, all-tuned, and ancestry-tuned Mult-t polygenic scores. The left panel displays a forest plot of the OR/SD increase in PGS for AF, with 95% CIs on the x-axis. The y-axis lists ancestry-specific cohorts from the All of Us validation dataset (Supplementary Data 1): EUR (n=124,367 of which 11,087 cases), AMR (n=40,219 of which 1,449 cases), AFR (n=44,692 of which 2,112 cases), SAS (n=3,806 of which 90 cases), and EAS (n=6,832 of which 198 cases), each paired with its PGSs. EUR, AMR, and AFR are based on the 70% validation subset, whereas the full 100% validation set was used for SAS and EAS due to limited sample size. The right panel summarizes key performance metrics. All estimates are based on logistic regression models adjusted for the first 20 PCs, age, and sex, except for AUROC and AUPRC values which are based on univariate models (to show prediction from PGS alone). *P*-values were calculated using two-sided Wald tests. Source Data are provided (Supplementary Data 4).

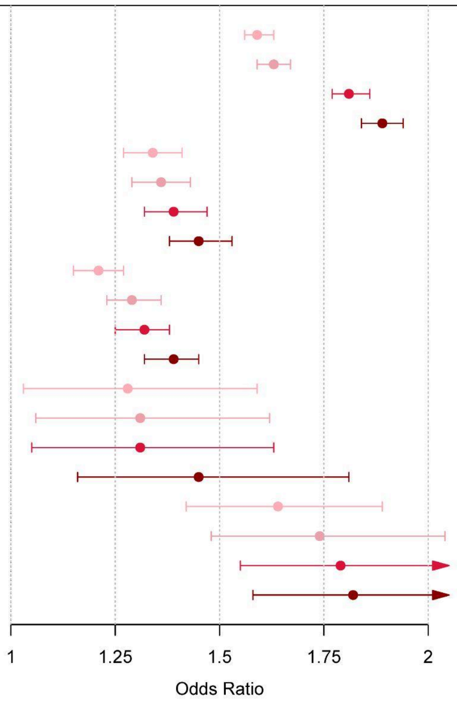

Cohort	PGS	OR [95% CI]	*P*-value	AUROC	AUPRC	Ngk-R^2	Liab-R^2
EUR	Roselli et al.	1.59 [1.56, 1.63]	<1.00E-300	0.615	0.135	0.040	0.060
	PRSmix+	1.63 [1.59, 1.67]	<1.00E-300	0.618	0.138	0.045	0.070
	ALLmeta	1.81 [1.77, 1.86]	<1.00E-300	0.643	0.153	0.064	0.098
	Mult-t-EUR	1.89 [1.84, 1.94]	<1.00E-300	0.648	0.155	0.071	0.109
AMR	Roselli et al.	1.34 [1.27, 1.41]	6.98E-28	0.578	0.050	0.012	0.024
	PRSmix+	1.36 [1.29, 1.43]	4.06E-30	0.583	0.049	0.014	0.026
	ALLmeta	1.39 [1.32, 1.47]	2.93E-37	0.591	0.053	0.017	0.032
	Mult-t-AMR	1.45 [1.38, 1.53]	7.11E-45	0.595	0.054	0.021	0.043
AFR	Roselli et al.	1.21 [1.15, 1.27]	1.43E-14	0.548	0.056	0.004	0.008
	PRSmix+	1.29 [1.23, 1.36]	3.17E-23	0.557	0.059	0.007	0.014
	ALLmeta	1.32 [1.25, 1.38]	3.76E-30	0.566	0.061	0.010	0.018
	Mult-t-AFR	1.39 [1.32, 1.45]	6.42E-43	0.573	0.064	0.014	0.028
SAS	Roselli et al.	1.28 [1.03, 1.59]	2.42E-02	0.583	0.030	0.008	0.015
	PRSmix+	1.31 [1.06, 1.62]	1.41E-02	0.587	0.032	0.009	0.020
	ALLmeta	1.31 [1.05, 1.63]	1.73E-02	0.579	0.030	0.009	0.020
	Mult-t-SAS	1.45 [1.16, 1.81]	1.29E-03	0.600	0.033	0.016	0.037
EAS	Roselli et al.	1.64 [1.42, 1.89]	1.90E-11	0.624	0.052	0.034	0.061
	PRSmix	1.74 [1.48, 2.04]	1.86E-11	0.617	0.048	0.034	0.061
	ALLmeta	1.79 [1.55, 2.07]	2.67E-15	0.657	0.055	0.047	0.075
	Mult-t-EAS	1.82 [1.58, 2.11]	4.55E-16	0.659	0.061	0.050	0.079

Supplementary Figure 8 | Comparison of the Roselli et al., PRSmix+, ALLmeta, and Mult-t polygenic scores. The left panel displays a forest plot of the OR/SD increase in PGS for AF, with 95% CIs on the x-axis. The y-axis lists ancestry-specific cohorts from the All of Us validation dataset: EUR (n=93,383 of which 8,189 cases), AMR (n=40,219 of which 1,449 cases), AFR (n=44,692 of which 2,112 cases), SAS (n=3,806 of which 90 cases), and EAS (n=6,832 of which 198 cases), each paired with its PGSs. For EUR, we excluded European individuals from the All of Us v6 Curated Data Repository (CDR) which were used to train the PRSmix+ PGS (Supplementary Data 9). AMR and AFR are based on the 70% validation dataset of the All of Us v8 CDR, whereas the full 100% validation set (v8) was used for SAS and EAS due to limited sample size (Supplementary Data 1). The right panel summarizes key performance metrics. All estimates are based on logistic regression models adjusted for the first 20 PCs, age, and sex, except for AUROC and AUPRC values which are based on univariate models (to show prediction from PGS alone). *P*-values were calculated using two-sided Wald tests. Source Data are provided (Supplementary Data 9).

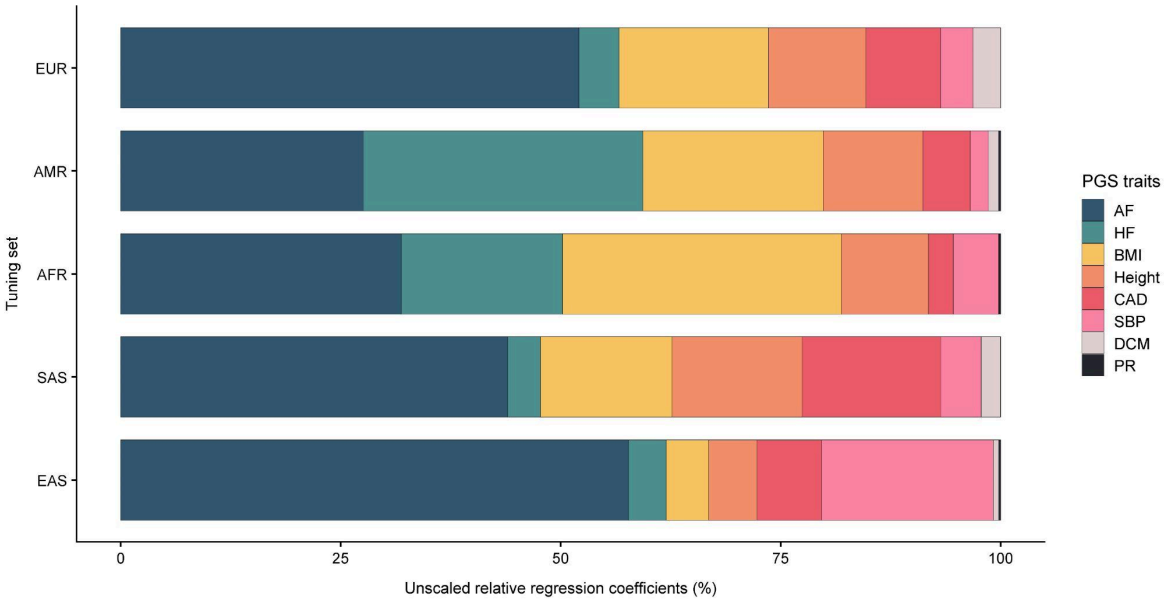

Supplementary Figure 9 | Unscaled relative regression coefficients of the Mult-t PGSs across ancestry-specific tuning sets. The plot illustrates the relative contribution of each of the eight traits (legend) to the final Mult-t scores across ancestry-specific tuning sets (y-axis): EUR (n=53,513 of which 4,775 cases), AMR (n=17,263 of which 613 cases), AFR (n=18,853 of which 877 cases), SAS (n=96,461 of which 6,463 cases), and EAS (n=19,459 of which 1,756 cases). EUR, AMR, and AFR are based on the 30% tuning subset in All of Us, whereas the SAS tuning set combines these subsets with 100% of EAS samples (Supplementary Data 1). EAS is based on its 30% tuning subset in BioBank Japan (Supplementary Data 5). The x-axis represents the unscaled relative regression coefficients expressed as percentages and normalized to 100% to reflect the trait composition of the Mult-t PGSs. Coefficients were derived from unadjusted logistic regression models based on each trait's predictive accuracy for AF in the corresponding tuning set. Source Data are provided (Supplementary Data 11).

Cohort	PGS	OR [95% CI]	*P*-value	AUROC	AUPRC	Ngk-R^2	Liab-R^2
EUR	AF	1.82 [1.78, 1.85]	<1.00E-300	0.642	0.155	0.065	0.098
	HF	1.38 [1.35, 1.41]	9.15E-204	0.558	0.107	0.019	0.036
	BMI	1.19 [1.16, 1.21]	1.11E-59	0.525	0.096	0.005	0.011
	Height	1.19 [1.16, 1.21]	1.51E-46	0.544	0.103	0.004	0.006
	CAD	1.13 [1.11, 1.16]	7.99E-32	0.521	0.096	0.003	0.006
	SBP	1.10 [1.07, 1.12]	1.31E-18	0.511	0.093	0.002	0.004
	DCM	1.04 [1.02, 1.06]	2.26E-04	0.508	0.092	0.000	0.001
	PR	1.02 [1.00, 1.04]	1.37E-01	0.504	0.091	0.000	0.000

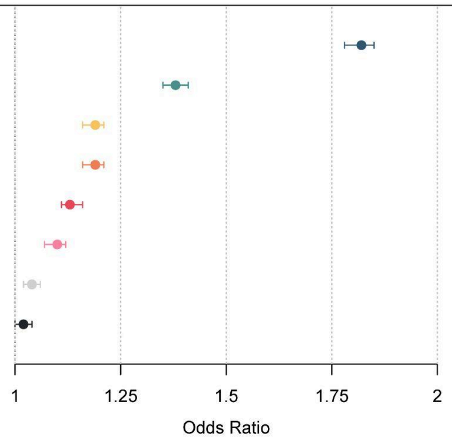

Supplementary Figure 10 | The contributing traits evaluated separately in the European validation set in AoU. The left panel displays a forest plot of the OR/SD increase in PGS for AF, with 95% CIs on the x-axis. The y-axis lists the European validation cohort alongside the PGSs for each of the eight traits. The right panel summarizes key performance metrics from the validation set. All estimates are based on logistic regression models adjusted for the first 20 PCs, age, and sex, applied to the 70% European validation dataset of All of Us (n=124,367 of which 11,087 cases). AUROC and AUPRC values are based on univariate models (to show prediction from PGS alone). *P*-values were calculated using two-sided Wald tests. Source Data are provided (Supplementary Data 12).

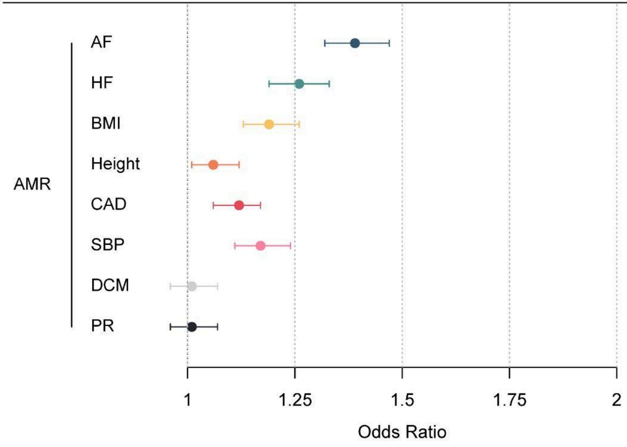

Cohort	PGS	OR [95% CI]	*P*-value	AUROC	AUPRC	Ngk-R^2	Liab-R^2
AMR	AF	1.39 [1.32, 1.47]	2.93E-37	0.591	0.053	0.017	0.032
	HF	1.26 [1.19, 1.33]	2.04E-17	0.554	0.044	0.008	0.018
	BMI	1.19 [1.13, 1.26]	6.44E-11	0.541	0.041	0.004	0.012
	Height	1.06 [1.01, 1.12]	2.04E-02	0.519	0.039	0.001	0.001
	CAD	1.12 [1.06, 1.17]	8.34E-06	0.530	0.040	0.002	0.005
	SBP	1.17 [1.11, 1.24]	3.59E-09	0.539	0.042	0.004	0.008
	DCM	1.01 [0.96, 1.07]	6.73E-01	0.500	0.037	0.000	0.000
	PR	1.01 [0.96, 1.07]	6.19E-01	0.501	0.036	0.000	0.000

Supplementary Figure 11 | The contributing traits evaluated separately in the Admixed American validation set in AoU. The left panel displays a forest plot of the OR/SD increase in PGS for AF, with 95% CIs on the x-axis. The y-axis lists the Admixed American validation cohort alongside the PGSs for each of the eight traits. The right panel summarizes key performance metrics from the validation set. All estimates are based on logistic regression models adjusted for the first 20 PCs, age, and sex, applied to the 70% Admixed American validation dataset of All of Us (n=40,219 of which 1,449 cases). AUROC and AUPRC values are based on univariate models (to show prediction from PGS alone). *P*-values were calculated using two-sided Wald tests. Source Data are provided (Supplementary Data 12).

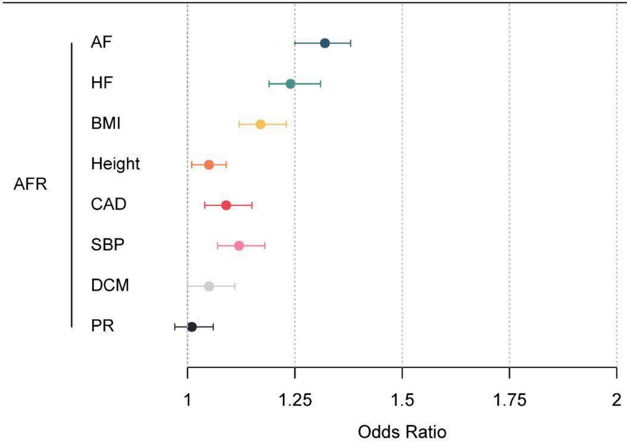

Cohort	PGS	OR [95% CI]	*P*-value	AUROC	AUPRC	Ngk-R^2	Liab-R^2
AFR	AF	1.32 [1.25, 1.38]	3.76E-30	0.566	0.061	0.010	0.018
	HF	1.24 [1.19, 1.31]	7.00E-20	0.547	0.056	0.006	0.015
	BMI	1.17 [1.12, 1.23]	2.34E-11	0.532	0.054	0.003	0.008
	Height	1.05 [1.01, 1.09]	1.36E-02	0.516	0.050	0.000	0.001
	CAD	1.09 [1.04, 1.15]	4.20E-04	0.517	0.050	0.001	0.002
	SBP	1.12 [1.07, 1.18]	1.24E-06	0.525	0.051	0.002	0.004
	DCM	1.05 [1.00, 1.11]	4.36E-02	0.515	0.049	0.000	0.001
	PR	1.01 [0.97, 1.06]	5.64E-01	0.504	0.049	0.000	0.000

Supplementary Figure 12 | The contributing traits evaluated separately in the African validation set in AoU. The left panel displays a forest plot of the OR/SD increase in PGS for AF, with 95% CIs on the x-axis. The y-axis lists the African validation cohort alongside the PGSs for each of the eight traits. The right panel summarizes key performance metrics from the validation set. All estimates are based on logistic regression models adjusted for the first 20 PCs, age, and sex, applied to the 70% African validation dataset of All of Us (n=44,692 of which 2,112 cases). AUROC and AUPRC values are based on univariate models (to show prediction from PGS alone). *P*-values were calculated using two-sided Wald tests. Source Data are provided (Supplementary Data 12).

Cohort	PGS	OR [95% CI]	*P*-value	AUROC	AUPRC	Ngk-R^2	Liab-R^2
SAS	AF	1.31 [1.05, 1.63]	1.73E-02	0.579	0.030	0.009	0.020
	HF	1.25 [0.99, 1.57]	5.96E-02	0.538	0.029	0.006	0.022
	BMI	1.26 [1.02, 1.57]	3.41E-02	0.558	0.028	0.007	0.015
	Height	1.09 [0.87, 1.36]	4.80E-01	0.528	0.027	0.001	0.000
	CAD	1.39 [1.12, 1.73]	2.96E-03	0.574	0.041	0.014	0.036
	SBP	1.19 [0.95, 1.50]	1.21E-01	0.549	0.030	0.004	0.011
	DCM	1.00 [0.81, 1.25]	9.84E-01	0.501	0.025	0.000	-0.001
	PR	1.02 [0.82, 1.26]	8.87E-01	0.501	0.027	0.000	-0.001

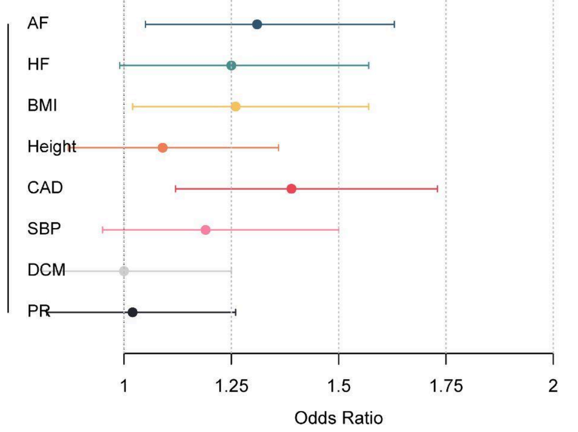

Supplementary Figure 13 | The contributing traits evaluated separately in the South-Asian validation set in AoU. The left panel displays a forest plot of the OR/SD increase in PGS for AF, with 95% CIs on the x-axis. The y-axis lists the African validation cohort alongside the PGSs for each of the eight traits. The right panel summarizes key performance metrics from the validation set. All estimates are based on logistic regression models adjusted for the first 20 PCs, age, and sex, applied to the 100% South-Asian validation dataset of All of Us (n=3,806 of which 90 cases). AUROC and AUPRC values are based on univariate models (to show prediction from PGS alone). *P*-values were calculated using two-sided Wald tests. Source Data are provided (Supplementary Data 12).

Cohort	PGS	OR [95% CI]	*P*-value	AUROC	AUPRC	Ngk-R^2	Liab-R^2
EAS	AF	1.79 [1.55, 2.07]	2.67E-15	0.657	0.055	0.047	0.075
	HF	1.69 [1.42, 2.03]	8.11E-09	0.615	0.044	0.025	0.046
	BMI	1.39 [1.20, 1.60]	9.29E-06	0.560	0.043	0.014	0.032
	Height	1.24 [1.08, 1.42]	2.72E-03	0.538	0.035	0.007	0.014
	CAD	1.06 [0.92, 1.23]	4.01E-01	0.521	0.031	0.001	0.000
	SBP	1.17 [0.99, 1.38]	5.96E-02	0.539	0.034	0.003	0.006
	DCM	1.08 [0.93, 1.27]	3.16E-01	0.526	0.032	0.001	0.001
	PR	1.04 [0.88, 1.23]	6.30E-01	0.499	0.029	0.000	0.001

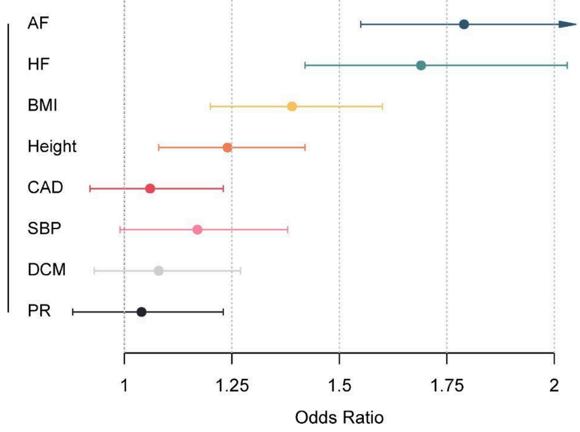

Supplementary Figure 14 | The contributing traits evaluated separately in the East-Asian validation set in AoU. The left panel displays a forest plot of the OR/SD increase in PGS for AF, with 95% CIs on the x-axis. The y-axis lists the African validation cohort alongside the PGSs for each of the eight traits. The right panel summarizes key performance metrics from the validation set. All estimates are based on logistic regression models adjusted for the first 20 PCs, age, and sex, applied to the 100% East-Asian validation dataset of All of Us (n=6,832 of which 198 cases). AUROC and AUPRC values are based on univariate models (to show prediction from PGS alone). *P*-values were calculated using two-sided Wald tests. Source Data are provided (Supplementary Data 12).

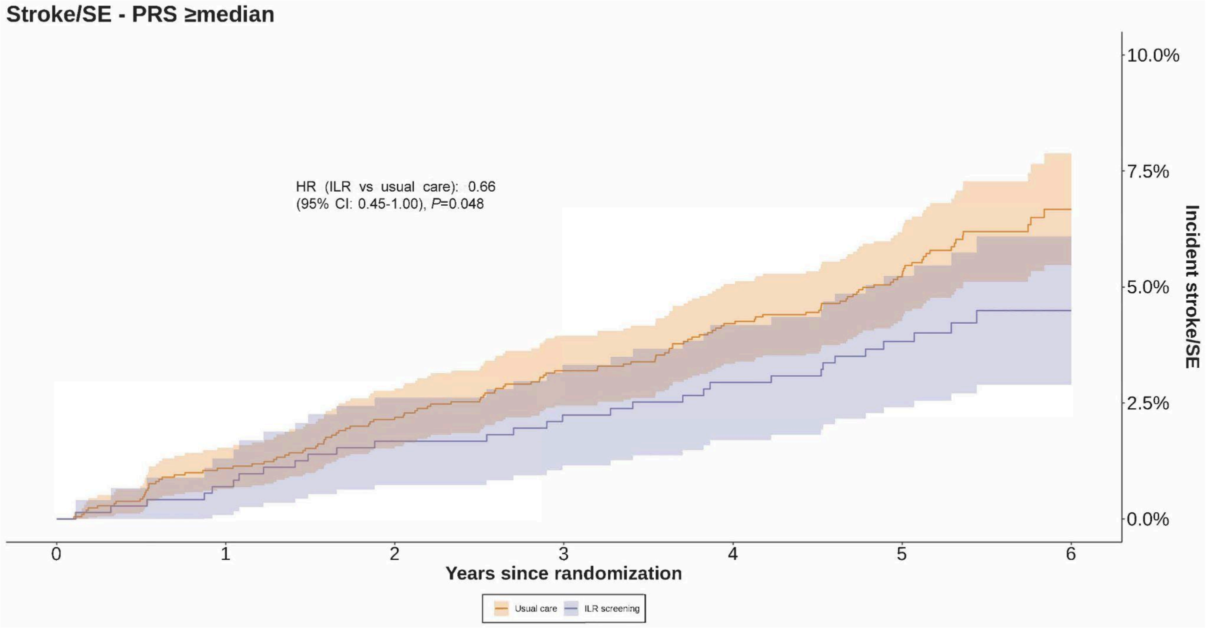

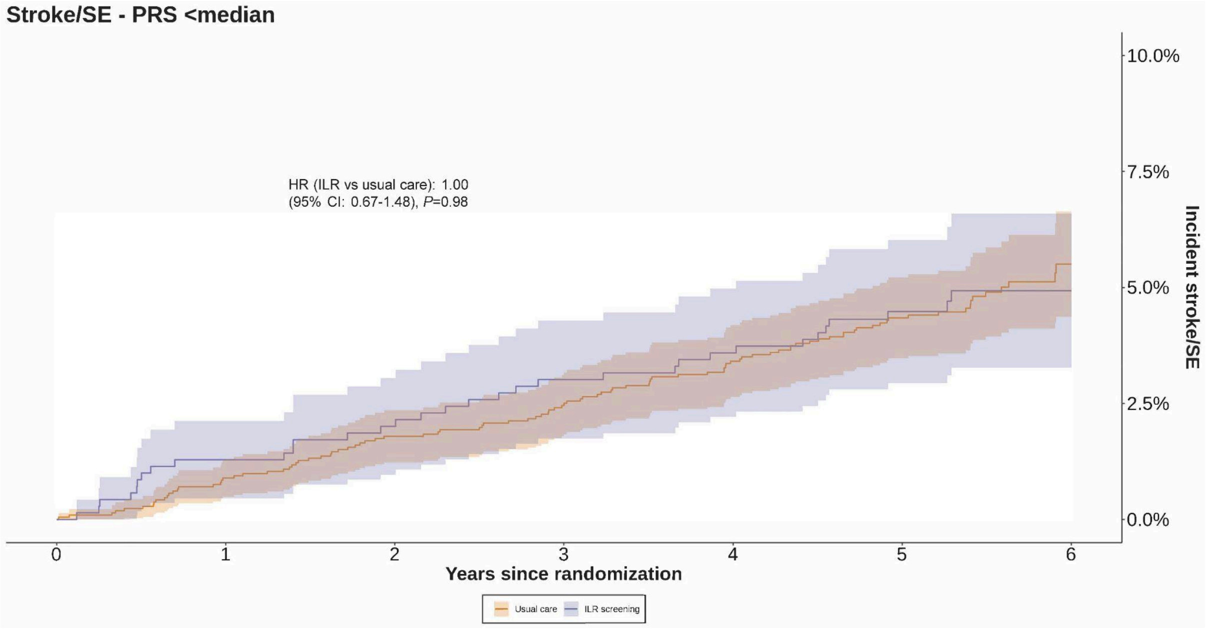

Supplementary Figure 15 | Incident risk of stroke or systemic embolism after randomization to either implantable loop recorder (ILR) screening or usual care, in the LOOP study, stratified by Mult-t-EUR PGS. PRS>=median (n=2,828 samples) indicates the group of participants with Mult-t-EUR score larger than or equal to the median, while PRS<median (n=2,828 samples) indicates the group of participants with Mult-t-EUR lower than the median. In both panels, x-axis shows years since randomization and y-axis shows incident rates of stroke or systemic embolism (SE). Purple line (with 95% confidence interval) indicates participants randomized to ILR screening, while the orange line (with 95% confidence interval) indicates the participants randomized to usual care. *P*-values were calculated using two-sided Wald tests. Individual-level data underlying the survival curves cannot be made publicly available.

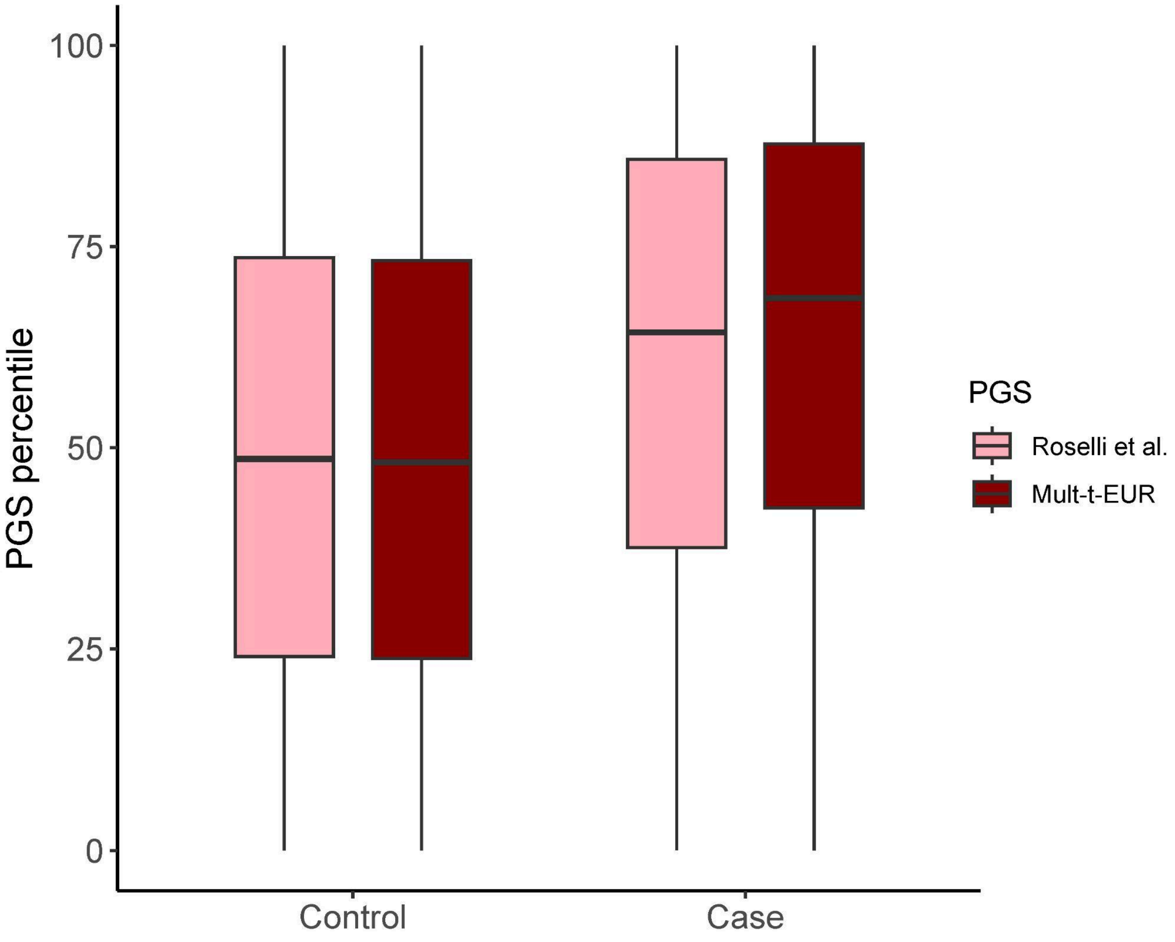

Supplementary Figure 16 | Distributions of cases and controls per PGS percentile in EUR ancestry of All of Us. Box plots display the distribution of controls (left) and AF cases (right) across PGS percentiles (y-axis). The Roselli et al. PGS is shown in pink, and the Mult-t-EUR PGS in dark red. Each box plot shows the median (center line), first and third quartiles (box edges), and whiskers extending up to 1.5 times the interquartile range beyond the box. Both scores were evaluated in the 70% European ancestry validation dataset of All of Us (n=124,367 of which 11,087 cases). Source Data are provided (Supplementary Data 28).

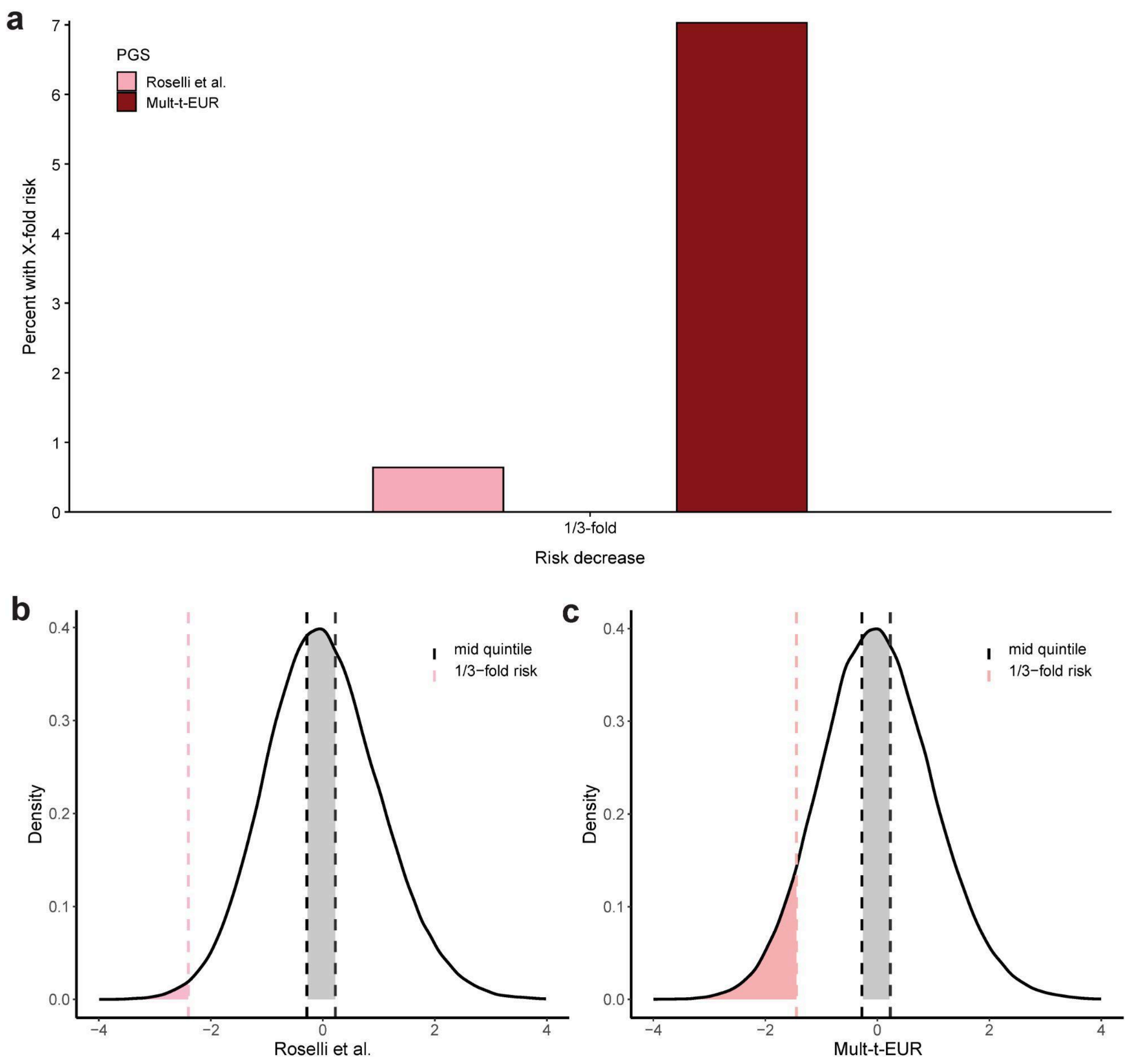

Supplementary Figure 17 | Proportion of EUR individuals from All of Us with 1/3-fold decreased AF risk compared to the middle quintile. All panels show the percentage of European ancestry participants in the 70% validation dataset of All of Us (n=124,367 of which 11,087 cases) with a 1/3-fold lower risk of AF relative to the middle PGS quintile. **a,** Bar plots display the proportion with a 1/3-fold lower risk for the Roselli et al. PGS (pink) and the Mult-t-EUR PGS (dark red). **b,c,** Density plots of the Roselli et al. (b) and Mult-t-EUR (c) PGSs. The x-axis displays polygenic score values, and the y-axis indicates density, representing the relative frequency of individuals with a specific polygenic score value. The middle quintile is shaded in grey, while the region corresponding to 1/3-fold risk is highlighted in a shade of red. Odds ratios were estimated using logistic regression adjusted for age, sex, and the first 20 ancestry PCs. Source Data are provided (Supplementary Data 18).

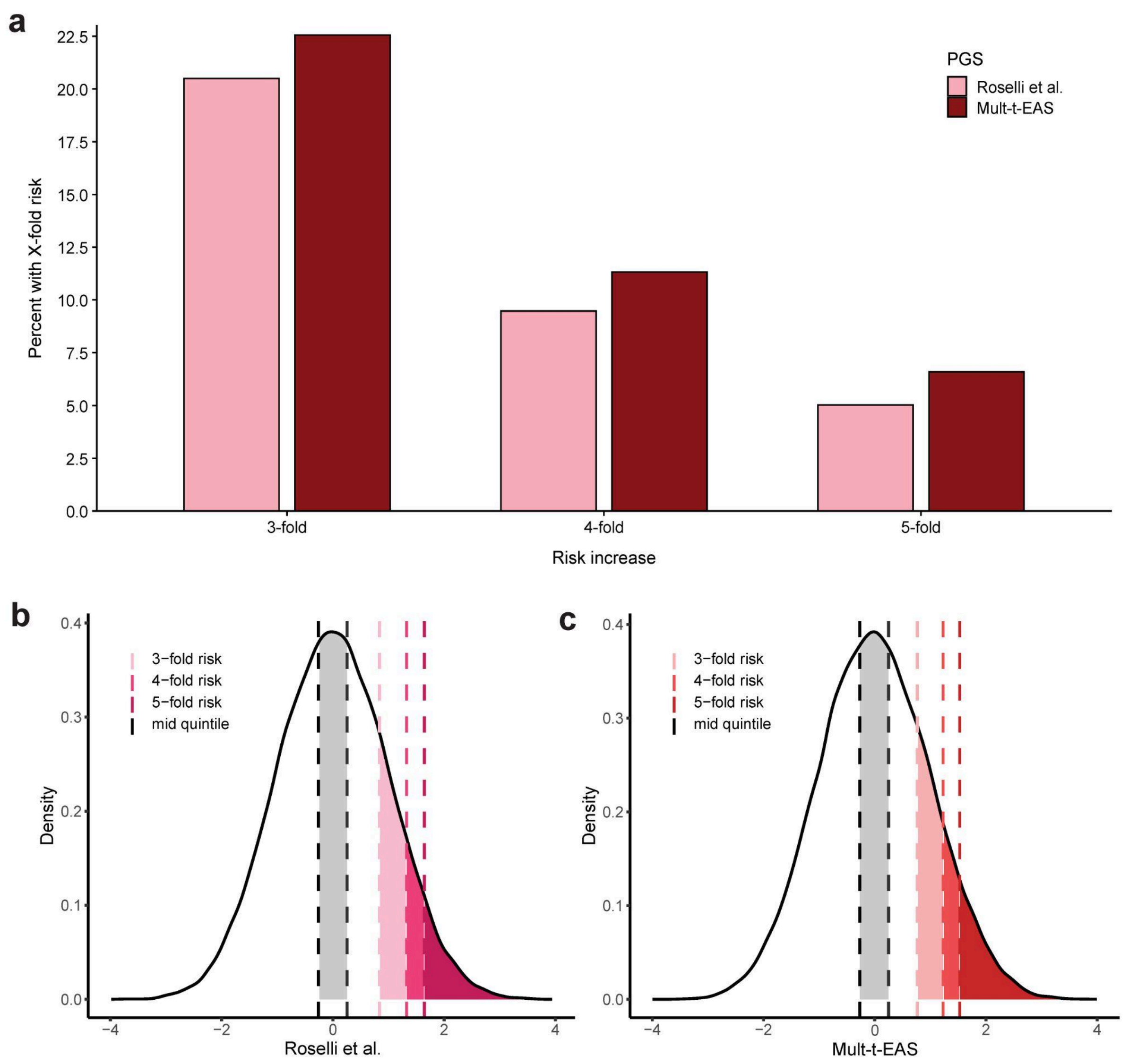

Supplementary Figure 18 | Proportion of individuals with 3-, 4-, or 5-fold increased AF risk compared to the middle quintile within BBJ. All panels show the percentage of East Asian ancestry participants in the 70% validation dataset of BioBank Japan 2nd cohort (n=45,404 of which 3,944 cases) with a 3-, 4-, or 5-fold higher risk of AF relative to the middle PGS quintile. **a,** Bar plots display the proportions in each risk group for the Roselli et al. PGS (pink) and the Mult-t-EAS PGS (dark red). **b,c,** Density plots of the Roselli et al. (b) and Mult-t-EAS (c) PGSs. The x-axis displays polygenic score values, and the y-axis indicates density, representing the relative frequency of individuals with a specific polygenic score value. The middle quintile is shaded in grey, while regions corresponding to 3-, 4-, and 5-fold risk are highlighted in progressively darker tones. Odds ratios were estimated using logistic regression adjusted for age, sex, and the first 20 ancestry PCs. Source Data are provided (Supplementary Data 29).

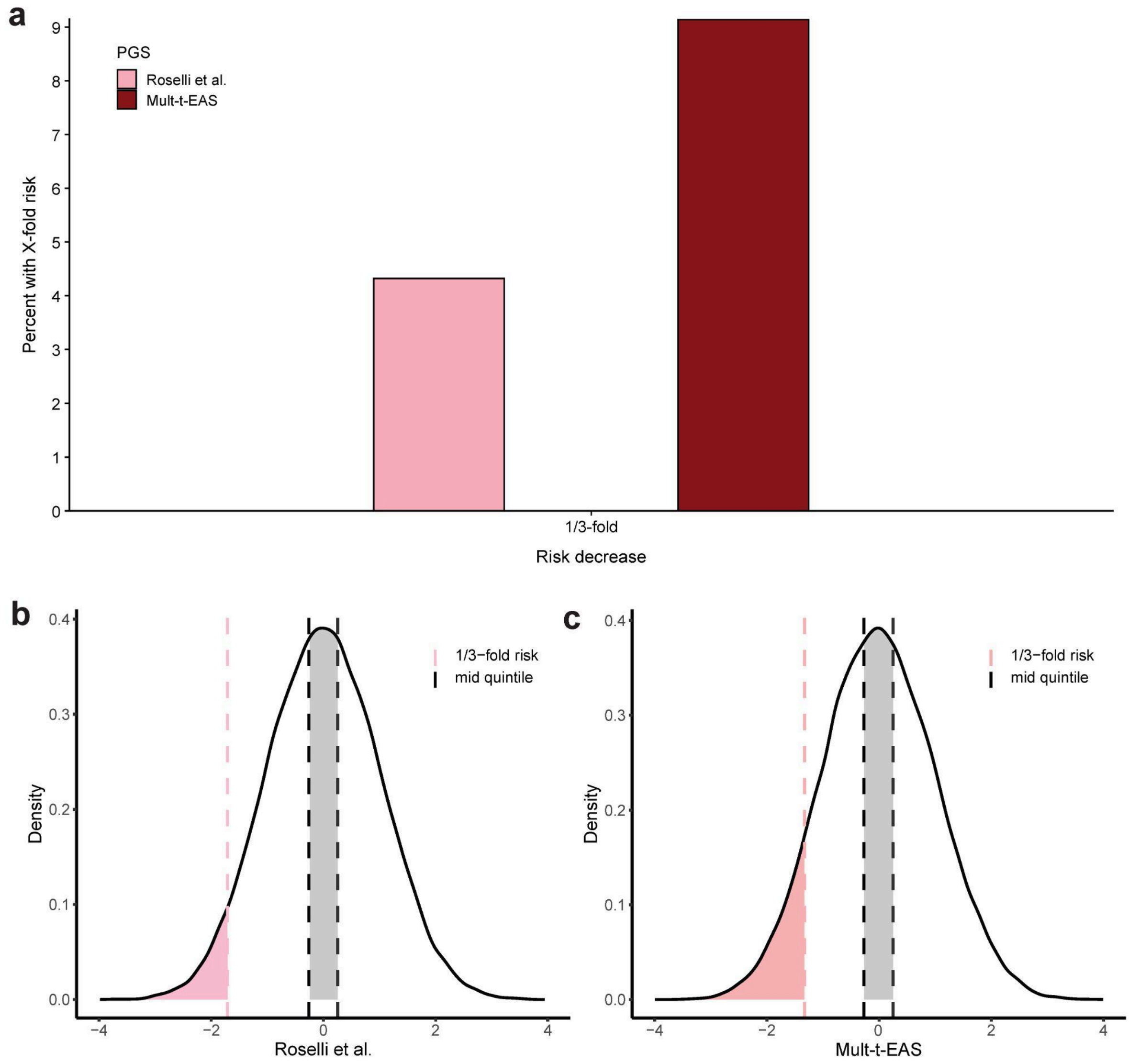

Supplementary Figure 19 | Proportion of individuals with 1/3-fold decreased AF risk compared to the middle quintile within BBJ. All panels show the percentage of East Asian ancestry participants in the 70% validation dataset of BioBank Japan 2nd cohort (n=45,404 of which 3,944 cases) with a 1/3-fold lower risk of AF relative to the middle PGS quintile. **a,** Bar plots display the proportion with a 1/3-fold lower risk for the Roselli et al. PGS (pink) and the Mult-t-EAS PGS (dark red). **b,c,** Density plots of the Roselli et al. (b) and Mult-t-EAS (c) PGSs. The x-axis displays polygenic score values, and the y-axis indicates density, representing the relative frequency of individuals with a specific polygenic score value. The middle quintile is shaded in grey, while the region corresponding to 1/3-fold risk is highlighted in a shade of red. Odds ratios were estimated using logistic regression adjusted for age, sex, and the first 20 ancestry PCs. Source Data are provided (Supplementary Data 29).

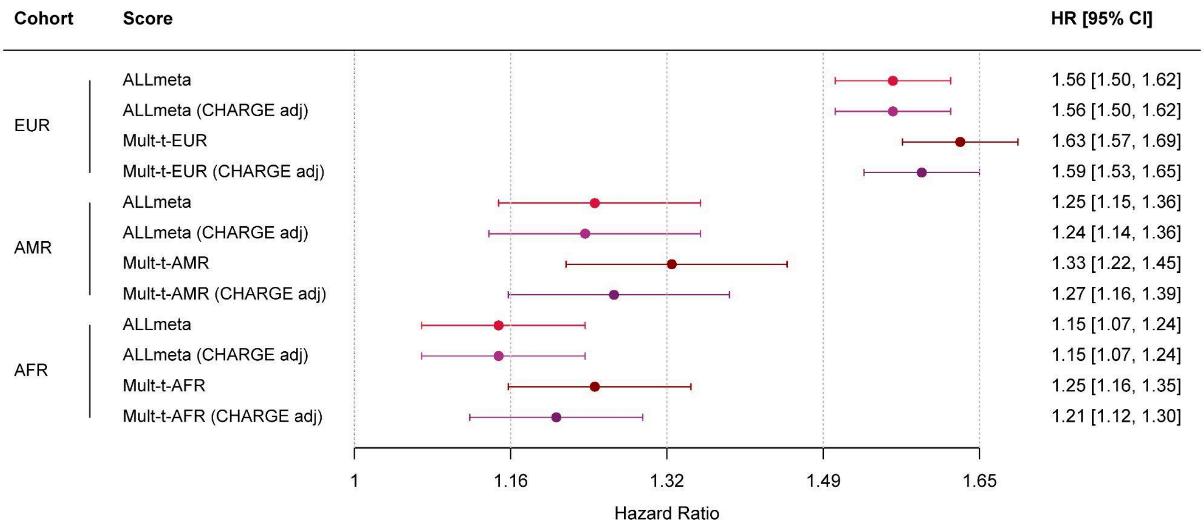

Supplementary Figure 20 | Hazard ratios for incident AF prediction using PGS-only and CHARGE-AF–adjusted PGS models. The left panel displays a forest plot of hazard ratio with 95% CIs on the x-axis. The y-axis lists ancestry-specific cohorts from All of Us (Supplementary Data 19): EUR (n=103,601 of which 2,749 incident cases), AMR (n=33,610 of which 449 incident cases), and AFR (n=39,697 of which 758 incident cases), each paired with its scores. The right panel displays the corresponding hazard ratio values numerically. The CHARGE-AF score is a clinical risk score for AF derived from multiple phenotypes (Supplementary Data 27). Estimates are based on cox regression models using PGS alone (adjusted for the first 20 PCs, age and sex) or models including the ALLmeta or multi-trait PGSs (each residualized for the first 20 PCs) with CHARGE-AF included as a covariate, with the full model adjusted for the first 10 PCs. Source Data are provided (Supplementary Data 19).

.

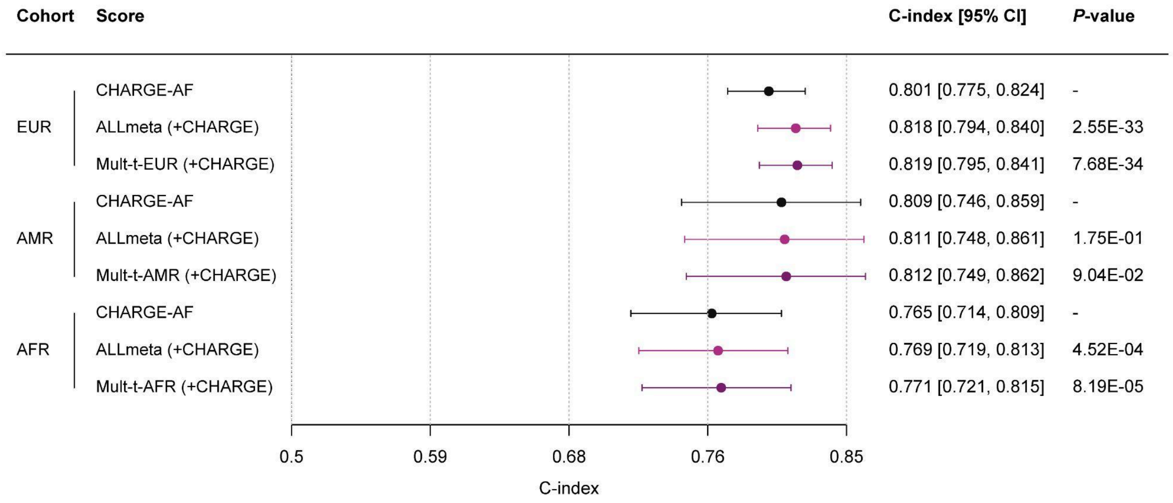

Supplementary Figure 21 | Harrell's C-indices for incident AF prediction using CHARGE-AF and PGS + CHARGE-AF models. The left panel displays a forest plot of Harrell's C-index with 95% CIs on the x-axis. The y-axis lists ancestry-specific cohorts from All of Us (Supplementary Data 19): EUR (n=103,601 of which 2,749 incident cases), AMR (n=33,610 of which 449 incident cases), and AFR (n=39,697 of which 758 incident cases), each paired with its scores. The right panel reports the C-index values numerically and the *P*-values for comparisons with the CHARGE-AF model using a one-sided Noether test. The CHARGE-AF score is a clinical risk score for atrial fibrillation derived from multiple phenotypes Supplementary Data 27). Estimates are based on cox regression models using CHARGE-AF alone (adjusted for the first 10 PCs) or models including the ALLmeta or multi-trait PGSs (each residualized for the first 20 PCs) with CHARGE-AF included as a covariate, with the full model adjusted for the first 10 PCs. Source Data are provided (Supplementary Data 20). *P*-values for model comparisons are available (Supplementary Data 23).

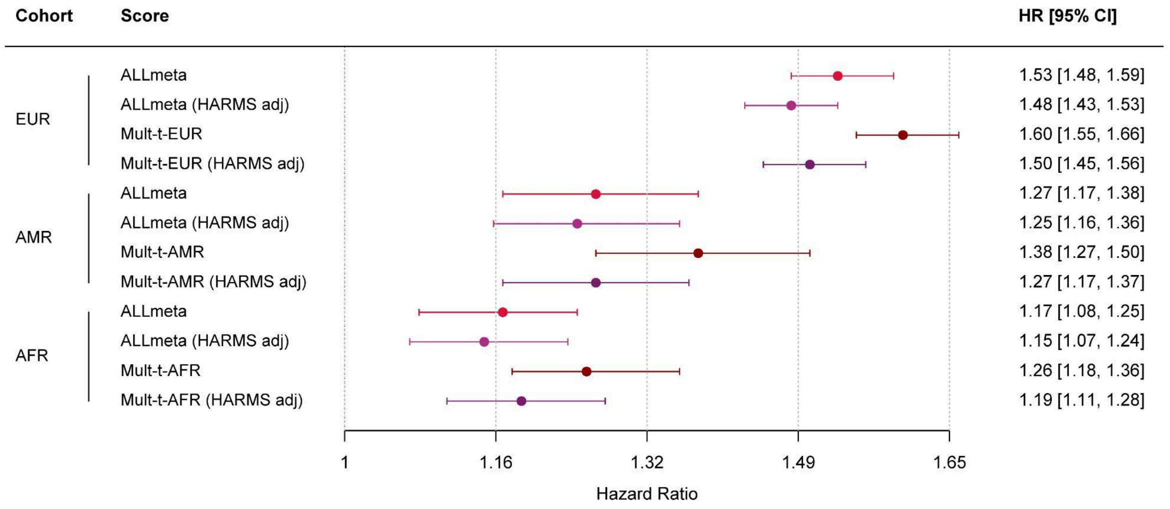

Supplementary Figure 22 | Hazard ratios for incident AF prediction using PGS-only and HARMS2-AF–adjusted PGS models. The left panel displays a forest plot of hazard ratio with 95% CIs on the x-axis. The y-axis lists ancestry-specific cohorts from All of Us (Supplementary Data 21): EUR (n=113,525 of which 3,006 incident cases), AMR (n=37,585 of which 509 incident cases), and AFR (n=40,984 of which 794 incident cases), each paired with its scores. The right panel displays the corresponding hazard ratio values numerically. The HARMS2-AF score is a clinical risk score for AF derived from multiple phenotypes (Supplementary Data 27). Estimates are based on cox regression models using PGS alone (adjusted for the first 20 PCs, age and sex) or models including the ALLmeta or multi-trait PGSs (each residualized for the first 20 PCs) with HARMS2-AF included as a covariate, with the full model adjusted for the first 10 PCs. Source Data are provided (Supplementary Data 21).

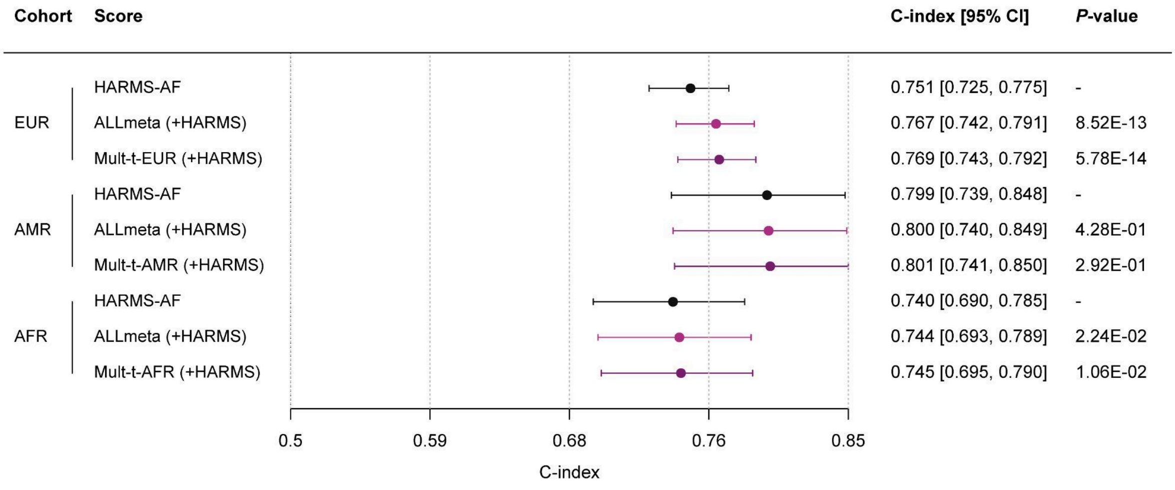

Supplementary Figure 23 | Harrell's C-indices for incident AF prediction using HARMS2-AF and PGS + HARMS2-AF models. The left panel displays a forest plot of Harrell's C-index with 95% CIs on the x-axis. The y-axis lists ancestry-specific cohorts from All of Us (Supplementary Data 21): EUR (n=113,525 of which 3,006 incident cases), AMR (n=37,585 of which 509 incident cases), and AFR (n=40,984 of which 794 incident cases), each paired with its scores. The right panel reports the C-index values numerically and the *P*-values for comparisons with the HARMS2-AF model using a one-sided Noether test. The HARMS2-AF score is a clinical risk score for atrial fibrillation derived from multiple phenotypes (Supplementary Data 27). Estimates are based on cox regression models using HARMS2-AF alone (adjusted for the first 10 PCs) or models including the ALLmeta or multi-trait PGSs (each residualized for the first 20 PCs) with HARMS2-AF included as a covariate, with the full model adjusted for the first 10 PCs. Source Data are provided (Supplementary Data 22). *P*-values for model comparisons are available (Supplementary Data 23).

Cohort	PGS	OR [95% CI]	*P*-value	AUROC	AUPRC	Ngk-R^2	Liab-R^2
EUR	Roselli et al.	1.60 [1.57, 1.64]	<1.00E-300	0.617	0.128	0.040	0.060
	ALLmeta	1.82 [1.77, 1.86]	<1.00E-300	0.644	0.144	0.064	0.097
	Mult-t-EUR	1.90 [1.85, 1.94]	<1.00E-300	0.649	0.147	0.071	0.110
AMR	Roselli et al.	1.35 [1.28, 1.43]	1.22E-27	0.583	0.049	0.014	0.026
	ALLmeta	1.40 [1.33, 1.48]	3.52E-35	0.594	0.052	0.018	0.033
	Mult-t-AMR	1.46 [1.39, 1.55]	4.89E-42	0.598	0.053	0.021	0.044
AFR	Roselli et al.	1.21 [1.15, 1.28]	1.32E-13	0.549	0.054	0.005	0.009
	ALLmeta	1.31 [1.24, 1.38]	8.38E-26	0.564	0.058	0.009	0.018
	Mult-t-AFR	1.39 [1.32, 1.46]	4.71E-38	0.572	0.061	0.014	0.028
SAS	Roselli et al.	1.26 [1.01, 1.58]	3.99E-02	0.578	0.031	0.007	0.012
	ALLmeta	1.26 [1.00, 1.59]	4.84E-02	0.567	0.031	0.007	0.015
	Mult-t-SAS	1.38 [1.09, 1.74]	6.6E-03	0.587	0.034	0.013	0.029
EAS	Roselli et al.	1.66 [1.42, 1.94]	3.67E-10	0.627	0.046	0.035	0.064
	ALLmeta	1.80 [1.54, 2.11]	2.32E-13	0.661	0.052	0.047	0.077
	Mult-t-EAS	1.82 [1.56, 2.14]	8.28E-14	0.662	0.057	0.049	0.080

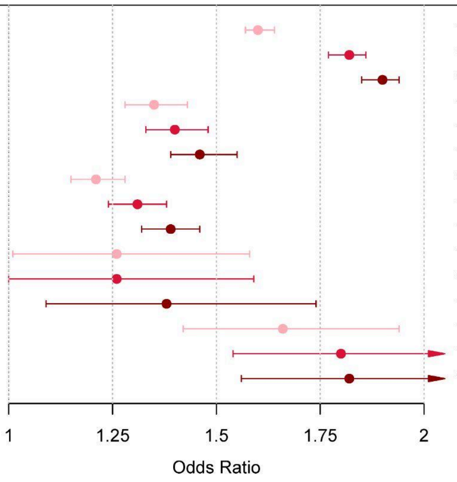

Supplementary Figure 24 | Sensitivity analysis excluding individuals from the state Massachusetts and with Veteran Affairs health coverage. The left panel displays a forest plot of the OR/SD increase in PGS for AF, with 95% CIs on the x-axis. The y-axis lists ancestry-specific cohorts from the All of Us validation dataset (Supplementary Data 24): EUR (n=104,730 of which 8,487 cases), AMR (n=37,134 of which 1,301 cases), AFR (n=40,930 of which 1,854 cases), SAS (n=3,325 of which 83 cases), and EAS (n=6,079 of which 163 cases), each paired with its PGSs. EUR, AMR, and AFR are based on the 70% validation subset, whereas the full 100% validation set was used for SAS and EAS due to limited sample size, excluding individuals from the state Massachusetts and with Veteran Affairs health coverage. The right panel summarizes key performance metrics. All estimates are based on logistic regression models adjusted for the first 20 PCs, age, and sex. AUROC and AUPRC values are based on univariate models (to show prediction from PGS alone). *P*-values were calculated using two-sided Wald tests. Source Data are provided (Supplementary Data 24).

Subset	Cases / Controls (%)	OR [95% CI]	*P*-value	AUROC	AUPRC	Ngk-R^2	Liab-R^2
None	11087 / 113280 (9.8%)	1.88 [1.84, 1.92]	<1.00E-300	0.646	0.157	0.070	0.108
Males	6516 / 43012 (15.1%)	1.93 [1.87, 1.98]	<1.00E-300	0.651	0.228	0.084	0.115
Females	4571 / 70268 (6.5%)	1.82 [1.76, 1.88]	<1.00E-300	0.646	0.111	0.058	0.092
Ages 45-54	365 / 15078 (2.4%)	1.66 [1.50, 1.83]	7.16E-23	0.632	0.049	0.031	0.069
Ages 55-64	1231 / 21320 (5.8%)	1.76 [1.67, 1.87]	7.06E-84	0.651	0.112	0.050	0.088
Ages 65-75	3722 / 30357 (12.3%)	1.95 [1.89, 2.03]	1.73E-297	0.676	0.221	0.085	0.119

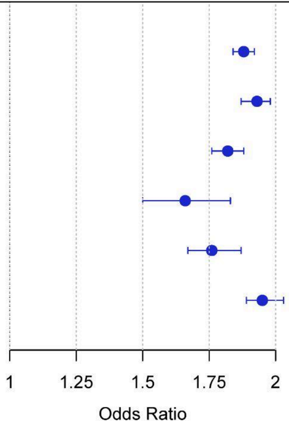

Supplementary Figure 25 | Subset analysis of the Mult-t-EUR score across sex and three different age ranges. The left panel displays a forest plot of the OR/SD increase in PGS for AF, with 95% CIs on the x-axis. The y-axis lists the analyzed subsets within the Mult-t-EUR PGS. The right panel summarizes case/control counts and key performance metrics for each validation subgroup. All estimates are based on logistic regression models adjusted for the first 20 PCs, age, and sex, applied to subgroups of the 70% European validation dataset of All of Us. AUROC and AUPRC values are based on univariate models (to show prediction from PGS alone). *P*-values were calculated using two-sided Wald tests. Source Data are provided (Supplementary Data 25).

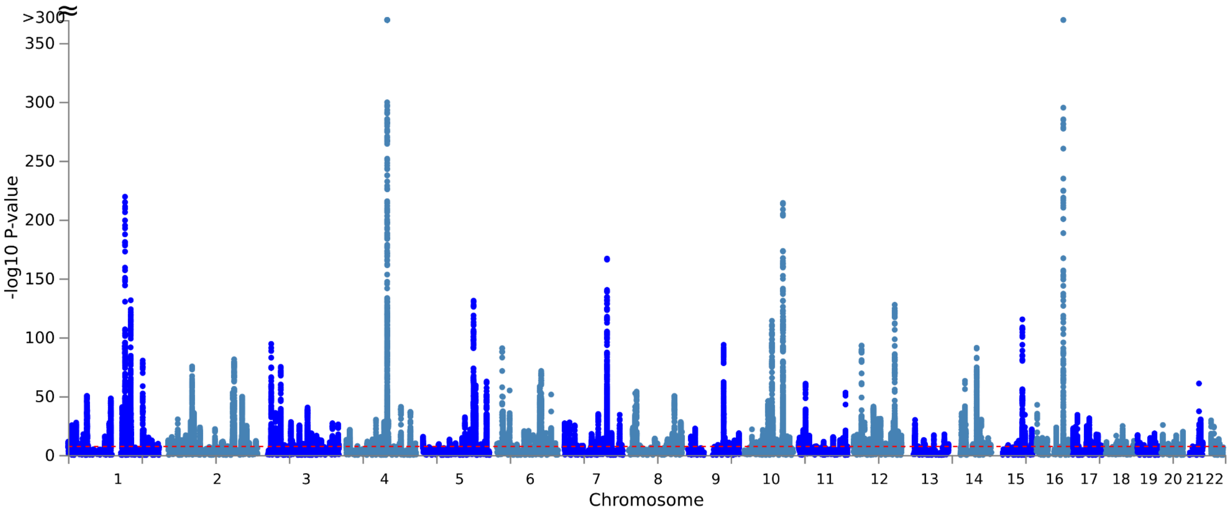

Supplementary Figure 26 | Manhattan plot for our AF GWAS meta-analysis discovering 486 loci. The figure shows a Manhattan plot with each dot representing a single variant association test, while the y-axis represents the -log10 of the association *P*-value for the given variant and the x-axis represents the genomic position of the variant (n=2,002,606 of which 269,746 cases). The dotted line shows the genome-wide significance (*P*<5x10-8) threshold. *P*-values were calculated using two-sided Wald tests. Variants in this plot are filtered to MAF>1%. A description of the GWAS loci and prioritized genes is provided (Supplementary Data 30).

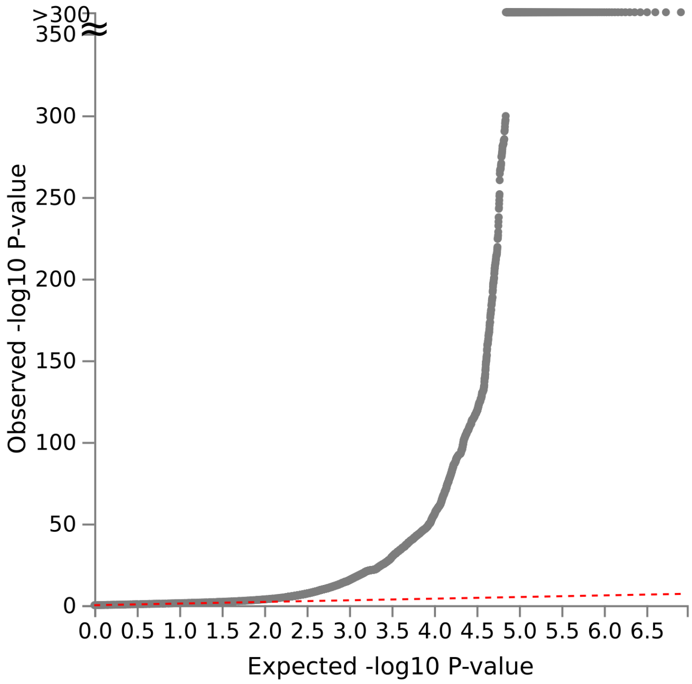

Supplementary Figure 27 | Quantile-quantile plot for our AF GWAS meta-analysis. The figure shows a quantile-quantile plot with each dot representing a single variant association test, while the y-axis represents the observed -log10 of the association *P*-value for the given variant and the x-axis expected -log10 of the association *P*-value under the null hypothesis of no association (n=2,002,606 of which 269,746 cases). *P*-values were calculated using two-sided Wald tests. The dotted red line shows x=y line.

Supplementary References

1. Roselli, C. *et al.* Meta-analysis of genome-wide associations and polygenic risk prediction for atrial fibrillation in more than 180,000 cases. *Nature Genetics* **57**, 539–547 (2025).
2. Khera, A. V. *et al.* Genome-wide polygenic scores for common diseases identify individuals with risk equivalent to monogenic mutations. *Nature Genetics* **50**, 1219–1224 (2018).
3. Gunn, S. *et al.* Comparison of methods for building polygenic scores for diverse populations. *Human Genetics and Genomics Advances* **6**, 100355 (2024).
4. Ruan, Y. *et al.* Improving polygenic prediction in ancestrally diverse populations. *Nature Genetics* **54**, 573–580 (2022).
5. Jermy, B. *et al.* A unified framework for estimating country-specific cumulative incidence for 18 diseases stratified by polygenic risk. *Nature Communications* **15**, 1–14 (2024).
6. Miyazawa, K. *et al.* Cross-ancestry genome-wide analysis of atrial fibrillation unveils disease biology and enables cardioembolic risk prediction. *Nature Genetics* **55**, 187–197 (2023).
7. Zhang, J. *et al.* An ensemble penalized regression method for multi-ancestry polygenic risk prediction. *Nature Communications* **15**, 1–14 (2024).
8. Ding, Y. *et al.* Polygenic scoring accuracy varies across the genetic ancestry continuum. *Nature* **618**, 774–781 (2023).
9. Troubat, L., Fettahoglu, D., Henches, L., Aschard, H. & Julienne, H. Multi-trait GWAS for diverse ancestries: mapping the knowledge gap. *BMC Genomics* **25**, 1–13 (2024).
10. Kavousi, M. & Ellinor, P. T. Polygenic risk scores for prediction of atrial fibrillation. *Netherlands Heart Journal* **31**, 1 (2022).
11. Kavousi, M. Differences in Epidemiology and Risk Factors for Atrial Fibrillation Between Women and Men. *Frontiers in Cardiovascular Medicine* **7**, 3 (2020).
12. Nagai, A. *et al.* Overview of the BioBank Japan Project: Study design and profile. *J*

Epidemiol **27**, S2–S8 (2017).

13. Åsvold, B. O. *et al.* Cohort Profile Update: The HUNT Study, Norway. *International Journal of Epidemiology* **52**, e80 (2022).
14. Brumpton, B. M. *et al.* The HUNT study: A population-based cohort for genetic research. *Cell Genomics* **2**, 100193 (2022).
15. Ferreira, M. A. *et al.* Shared genetic origin of asthma, hay fever and eczema elucidates allergic disease biology. *Nature genetics* **49**, 1752 (2017).
16. Svendsen, J. H. *et al.* Implantable loop recorder detection of atrial fibrillation to prevent stroke (The LOOP Study): a randomised controlled trial. *Lancet (London, England)* **398**, (2021).
17. Vad, O. B. *et al.* Atrial Fibrillation Screening According to Genetic Risk: A Secondary Analysis of the Randomized LOOP Study. *Journal of the American College of Cardiology* (2025) doi:10.1016/j.jacc.2025.09.024.
18. GitHub - poeyahay/01. AFGen+MVP.sh. *GitHub* https://github.com/poeyahay/AF_MultiTrait_PGS/blob/main/01.%20METAL/01.%20AFGen%2BMVP.sh (2025).
19. GitHub - poeyahay/02. SBP_trait.sh. *GitHub* https://github.com/poeyahay/AF_MultiTrait_PGS/blob/main/01.%20METAL/02.%20SBP_trait.sh (2025).
20. Howell, C. R. *et al.* Maximum Lifetime Body Mass Index and Mortality in Mexican American Adults: the National Health and Nutrition Examination Survey III (1988–1994) and NHANES 1999–2010. *Preventing Chronic Disease* **14**, E67 (2017).
21. Márquez-Luna, C. *et al.* Incorporating functional priors improves polygenic prediction accuracy in UK Biobank and 23andMe data sets. *Nature Communications* **12**, 1–11 (2021).
22. GitHub - zhilizheng/SBayesRC. *GitHub* https://github.com/zhilizheng/SBayesRC (2024).
23. GitHub - poeyahay/SBRC_Run.sh. *GitHub* https://github.com/poeyahay/AF_MultiTrait_PGS/blob/main/02.%20SBayesRC/SBRC_R

un.sh (2025).

24. GitHub - poeyahay/01. Reference_Allele_Flip.sh. *GitHub* https://github.com/poeyahay/AF_MultiTrait_PGS/blob/main/03.%20Scoring%20with%20PLINK2/01.%20Reference_Allele_Flip.sh (2026)
25. GitHub - poeyahay/SBRCmulti.R. *GitHub* https://github.com/poeyahay/AF_MultiTrait_PGS/blob/main/05.%20Multi%20tools/01.%20SBRCmulti.R (2025).
26. GitHub - poeyahay/Multi_Tool.R. *GitHub* https://github.com/poeyahay/AF_MultiTrait_PGS/blob/main/05.%20Multi%20tools/02.%20Multi_Tool.R (2025).
27. Lee, S. H., Goddard, M. E., Wray, N. R. & Visscher, P. M. A better coefficient of determination for genetic profile analysis. *Genetic epidemiology* **36**, (2012).
28. Schipper, M. *et al.* Prioritizing effector genes at trait-associated loci using multimodal evidence. *Nat Genet* **57**, 323–333 (2025).
29. Yuan, S. *et al.* Cross-population GWAS and proteomics improve risk prediction and reveal mechanisms in atrial fibrillation. *Nat Commun* **16**, 6426 (2025).
