# Supplementary material for: Multi-trait polygenic risk scores improve genomic prediction of atrial fibrillation across diverse ancestries
Source: Nat Commun. 2026 May 5;17:6059. doi: 10.1038/s41467-026-72708-x (PMC13350093; doi:10.1038/s41467-026-72708-x)
Supplement: Supplementary file 2 — Description of Additional Supplementary Files [file 41467_2026_72708_MOESM2_ESM.pdf]

Description of Additional Supplementary Files

Supplementary Data 1. AF, atrial fibrillation; AF_incident, incident atrial fibrillation (post baseline measurements); AFlut, atrial flutter (without AF); Age, age in years (at analysis); AFR, African; AMR, Admixed American; BMI, body mass index; Case, has AF; CMP, cardiomyopathy; Control, does not have AF; DBP, diastolic blood pressure; EAS, East Asian; EUR, European; HF, heart failure; HPT, hypertension; IHD, ischemic heart disease; SAS, South Asian; SAS-excl., SAS-excluded, 30% EUR/AFR/AMR and 100% EAS used; SBP, systolic blood pressure; Tune, calculated in 30% tuning set; Val, calculated in 70% validation / testing set.

Supplementary Data 2. AFR, African; ALL, all-ancestry; AMR, Admixed American; AUROC, Area Under the Receiver Operator Curve; AUPRC, Area Under the Precision Recall Curve; Beta, effect size; CI.H.OR/AUROC, Confidence Interval Upper Bound; CI.L.OR/AUROC, Confidence Interval Lower Bound; EUR, European; .full, full model adjusted for age and sex, using PGS residualized for the first 20 principal components (PCs) of ancestry; liab_R2_delta_o_resid, Liability delta R^2 ($\Delta R^2 = R^2\_full - R^2\_base$) divided by the residual liability R^2 ($1 - R^2\_base$); -meta, derived from the AFGen+MVP meta-analysis; Mult-a, multi-ancestry; ngk_R2_delta_o_resid, Nagelkerke's delta R^2 divided by the residual Nagelkerke's R^2; OR, Odds Ratio; P, p-values calculated using two-sided Wald tests; PGS, Polygenic Score; .prs, univariate (PGS-only) model using PGS residualized for the first 20 PCs; SE, standard error.

Supplementary Data 3. AFR, African; ALL, all-ancestry; AMR, Admixed American; AUROC, Area Under the Receiver Operator Curve; AUPRC, Area Under the Precision Recall Curve; Beta, effect size; CI.H.OR/AUROC, Confidence Interval Upper Bound; CI.L.OR/AUROC, Confidence Interval Lower Bound; EUR, European; .full, full model adjusted for age and sex, using PGS residualized for the first 20 principal components (PCs) of ancestry; liab_R2_delta_o_resid, Liability delta R^2 ($\Delta R^2 = R^2\_full - R^2\_base$) divided by the residual liability R^2 ($1 - R^2\_base$); -meta, derived from the AFGen+MVP meta-analysis; Mult-m, multi-method; ngk_R2_delta_o_resid, Nagelkerke's delta R^2 divided by the residual Nagelkerke's R^2; OR, Odds Ratio; P, p-values calculated using two-sided Wald tests; PGS, Polygenic Score; .prs, univariate (PGS-only) model using PGS residualized for the first 20 PCs; SE, standard error.

Supplementary Data 4. AFR, African; ALL, all-ancestry; AMR, Admixed American; AUROC, Area Under the Receiver Operator Curve; AUPRC, Area Under the Precision Recall Curve; Beta, effect size; CI.H.OR/AUROC, Confidence Interval Upper Bound; CI.L.OR/AUROC, Confidence Interval Lower Bound; EAS, East Asian; EUR, European; .full, full model adjusted for age and sex, using PGS residualized for the first 20 principal components (PCs) of ancestry; liab_R2_delta_o_resid, Liability delta R^2 ($\Delta R^2 = R^2\_full - R^2\_base$) divided by the residual liability R^2 ($1 - R^2\_base$); -meta, derived from the AFGen+MVP meta-analysis; Mult-t, multi-trait; ngk_R2_delta_o_resid, Nagelkerke's delta R^2 divided by the residual Nagelkerke's R^2; OR, Odds Ratio; P, p-values calculated using two-sided Wald tests; PGS, Polygenic Score; .prs, univariate (PGS-only) model using PGS residualized for the first 20 PCs; SAS, South Asian; SE, standard error.

Supplementary Data 5. AF, atrial fibrillation; Age, age in years (at analysis); BBJ, Biobank Japan 2nd cohort; BMI, body mass index; Case, has AF; CMP, cardiomyopathy; Control, does not have AF; DBP, diastolic blood pressure; HF, heart failure; HPT, hypertension; IHD, ischemic heart disease; SBP, systolic blood pressure; Tune, calculated in 30% tuning set; Val, calculated in 70% validation / testing set.

Supplementary Data 6. ALL (total) sample size numbers sometimes do not represent the sum of the ancestry-specific numbers, due to additional groups/samples having contributed to the ALL meta-analysis. For instance, for Roselli, Finnish and Icelandic samples were not included in EUR, but did contribute to ALL.

Abbreviations: AFR, African; ALL, all-ancestry; AMR, Admixed American; AoU, All of Us; ASN, EAS, East Asian; EUR, European; GCST-, GWAS catalog accession ID; METAL, meta-analysis tool; MVP, Million Veteran Program; Mult-t, multi-trait; Nature Gen / Comm / Med, Nature Genetics / Communications / Medicine; PGS, Polygenic Score; phs-, dbGAP accession ID; SAS, South Asian; UKB, UK-Biobank.

Supplementary Data 7. AFR, African; ALL, all-ancestry; AMR, Admixed American; AUROC, Area Under the Receiver Operator Curve; AUPRC, Area Under the Precision Recall Curve; Beta, effect size; CI.H.OR/AUROC, Confidence Interval Upper Bound; CI.L.OR/AUROC, Confidence Interval Lower Bound; DeLong, reports P-values for comparisons in AUROC with the Roselli et al. PGS using the one-sided DeLong test; EAS, East Asian; EUR, European; .full, full model adjusted for age and sex, using PGS residualized for the first 20 principal components (PCs) of ancestry; liab_R2_delta_o_resid, Liability delta R^2 ($\Delta R^2 = R^2\_full - R^2\_base$) divided by the residual liability R^2 ($1 - R^2\_base$); -meta, derived from the AFGen+MVP meta-analysis; Mult-t, multi-trait; ngk_R2_delta_o_resid, Nagelkerke's delta R^2 divided by the residual Nagelkerke's R^2; OR, Odds Ratio; P, p-values calculated using two-sided Wald tests; PGS, Polygenic Score; .prs, univariate (PGS-only) model using PGS residualized for the first 20 PCs; SAS, South Asian; SE, standard error.

Supplementary Data 8. AFR, African; ALL, all-ancestry; AMR, Admixed American; AUROC, Area Under the Receiver Operator Curve; AUPRC, Area Under the Precision Recall Curve; Beta, effect size; CI.H.OR/AUROC, Confidence Interval Upper Bound; CI.L.OR/AUROC, Confidence Interval Lower Bound; EAS, East Asian; EUR, European; .full, full model with age and sex as covariates; liab_R2_delta_o_resid, Liability delta R^2 ($\Delta R^2 = R^2\_full - R^2\_base$) divided by the residual liability R^2 ($1 - R^2\_base$); -meta, derived from the AFGen+MVP meta-analysis; Mult-t, multi-trait; ngk_R2_delta_o_resid, Nagelkerke's delta R^2 divided by the residual Nagelkerke's R^2; OR, Odds Ratio; P, p-value; PGS, Polygenic Score; .prs, univariate (PGS-only) model; SAS, South Asian; SE, standard error.

Supplementary Data 9. AFR, African; ALL, all-ancestry; AMR, Admixed American; AUROC, Area Under the Receiver Operator Curve; AUPRC, Area Under the Precision Recall Curve; Beta, effect size; CI.H.OR/AUROC, Confidence Interval Upper Bound; CI.L.OR/AUROC, Confidence Interval Lower Bound; EAS, East Asian; EUR, European; .full, full model adjusted for age and sex, using PGS residualized for the first 20 principal components (PCs) of ancestry; liab_R2_delta_o_resid, Liability delta R^2 ($\Delta R^2 = R^2\_full - R^2\_base$) divided by the residual liability R^2 ($1 - R^2\_base$); -meta, derived from the AFGen+MVP meta-analysis; Mult-t, multi-trait; ngk_R2_delta_o_resid, Nagelkerke's delta R^2 divided by the residual Nagelkerke's R^2; OR, Odds Ratio; P, p-values calculated using two-sided Wald tests; PGS, Polygenic Score; .prs, univariate (PGS-only) model using PGS residualized for the first 20 PCs; SAS, South Asian; SE, standard error.

Supplementary Data 10. AF, atrial fibrillation; BMI, body mass index; CAD, coronary artery disease; DCM, dilated cardiomyopathy; HF, heart failure; Mixing Proportion, coefficients normalized to sum to 100%; Mixing Weight, scaled regression coefficients; PGS, Polygenic Score; PR, PR-interval; SBP, systolic blood pressure; SD in Tune, standard deviation of the PGS in the tuning set.

Supplementary Data 11. AF, atrial fibrillation; BMI, body mass index; CAD, coronary artery disease; DCM, dilated cardiomyopathy; HF, heart failure; Ngk-R^2 in Tune, Nagelkerke's R^2 of the PRS in the tuning set; PGS, Polygenic Score; PR, PR-interval; Proportion, coefficients normalized to sum to 100%; Regcoef, unscaled regression coefficients; SBP, systolic blood pressure.

Supplementary Data 12. AF, atrial fibrillation; AFR, African; ALL, all-ancestry; AMR, Admixed American; AUROC, Area Under the Receiver Operator Curve; AUPRC, Area Under the Precision Recall Curve; Beta, effect size; BMI, body mass index; CAD, coronary artery disease; CI.H.OR/AUROC, Confidence Interval Upper Bound; CI.L.OR/AUROC, Confidence Interval Lower Bound; DCM, dilated cardiomyopathy; EAS, East Asian;

EUR, European; .full, full model with age and sex as covariates; HF, heart failure; liab_R2_delta_o_resid, Liability delta R^2 (ΔR^2 = R^2_full − R^2_base) divided by the residual liability R^2 (1 − R^2_base); ngk_R2_delta_o_resid, Nagelkerke's delta R^2 divided by the residual Nagelkerke's R^2; OR, Odds Ratio; P, p-value; PGS, Polygenic Score; PR, PR-interval; .prs, univariate (PGS-only) model; SAS, South Asian; SBP, systolic blood pressure; SE, standard error.

Supplementary Data 13. ALL, all-ancestry; AUROC, Area Under the Receiver Operator Curve; AUPRC, Area Under the Precision Recall Curve; BBJ, Biobank Japan 2nd cohort; Beta, effect size; CI.H.OR/AUROC, Confidence Interval Upper Bound; CI.L.OR/AUROC, Confidence Interval Lower Bound; EAS, East Asian; .full, full model adjusted for age and sex, using PGS residualized for the first 20 principal components (PCs) of ancestry; liab_R2_delta_o_resid, Liability delta R^2 (ΔR^2 = R^2_full − R^2_base) divided by the residual liability R^2 (1 − R^2_base); -meta, derived from the AFGen+MVP meta-analysis; Mult-t, multi-trait; ngk_R2_delta_o_resid, Nagelkerke's delta R^2 divided by the residual Nagelkerke's R^2; OR, Odds Ratio; P, p-values calculated using two-sided Wald tests; PGS, Polygenic Score; .prs, univariate (PGS-only) model using PGS residualized for the first 20 PCs; SE, standard error.

Supplementary Data 14. AUROC, Area Under the Receiver Operator Curve; AUPRC, Area Under the Precision Recall Curve; Beta, effect size; CI.H.OR/AUROC, Confidence Interval Upper Bound; CI.L.OR/AUROC, Confidence Interval Lower Bound; EUR, European; .full, full model adjusted for age and sex, using PGS residualized for the first 20 principal components (PCs) of ancestry; liab_R2_delta_o_resid, Liability delta R^2 (ΔR^2 = R^2_full − R^2_base) divided by the residual liability R^2 (1 − R^2_base); -meta, derived from the AFGen+MVP meta-analysis; Mult-t, multi-trait; ngk_R2_delta_o_resid, Nagelkerke's delta R^2 divided by the residual Nagelkerke's R^2; OR, Odds Ratio; P, p-values calculated using two-sided Wald tests; PGS, Polygenic Score; .prs, univariate (PGS-only) model using PGS residualized for the first 20 PCs; SE, standard error.

Supplementary Data 15. AUROC, Area Under the Receiver Operator Curve; AUPRC, Area Under the Precision Recall Curve; Beta, effect size; CI.H.OR/AUROC, Confidence Interval Upper Bound; CI.L.OR/AUROC, Confidence Interval Lower Bound; EUR, European; .full, full model adjusted for age and sex, using PGS residualized for the first 6 principal components (PCs) of ancestry; liab_R2_delta_o_resid, Liability delta R^2 (ΔR^2 = R^2_full − R^2_base) divided by the residual liability R^2 (1 − R^2_base); Mult-t, multi-trait; ngk_R2_delta_o_resid, Nagelkerke's delta R^2 divided by the residual Nagelkerke's R^2; OR, Odds Ratio; P, p-values calculated using two-sided Wald tests; PGS, Polygenic Score; .prs, univariate (PGS-only) model using PGS residualized for the first 6 PCs; SE, standard error.

Supplementary Data 16. AFR, African; ASN, Asian (East Asian + South Asian); AUROC, Area Under the Receiver Operator Curve; AUPRC, Area Under the Precision Recall Curve; Beta, effect size; CI.H.OR/AUROC, Confidence Interval Upper Bound; CI.L.OR/AUROC, Confidence Interval Lower Bound; .full, full model adjusted for age and sex, using PGS residualized for the first 20 principal components (PCs) of ancestry; liab_R2_delta_o_resid, Liability delta R^2 (ΔR^2 = R^2_full − R^2_base) divided by the residual liability R^2 (1 − R^2_base); Mult-t, multi-trait; ngk_R2_delta_o_resid, Nagelkerke's delta R^2 divided by the residual Nagelkerke's R^2; OR, Odds Ratio; P, p-values calculated using two-sided Wald tests; PGS, Polygenic Score; .prs, univariate (PGS-only) model using PGS residualized for the first 20 PCs; SE, standard error.

Supplementary Data 17. No abbreviations were used; therefore, no description is provided.

Supplementary Data 18. EUR, European; Mult-t, multi-trait; PGS, Polygenic score; 3×, 4×, 5×, and ⅓× denote fold-changes in AF risk relative to the middle PGS quintile (%).

Supplementary Data 19. AFR, African; ALL, all-ancestry; AMR, Admixed American; CHARGE, clinical risk score (CHARGE-AF) for atrial fibrillation; CHARGE adj, adjusted for CHARGE-AF; CI.H.HR, Confidence Interval

Upper Bound; CI.L.HR, Confidence Interval Lower Bound; EUR, European; HR, Hazard Ratio; -meta, derived from the AFGen+MVP meta-analysis; Mult-t, multi-trait; P, p-value; Score, models using PGS alone (adjusted for the first 20 principal components (PCs), age and sex) or models including the PGS (each residualized for the first 20 PCs) with CHARGE-AF included as a covariate, with the full model adjusted for the first 10 PCs; SE, standard error.

Supplementary Data 20. AFR, African; ALL, all-ancestry; AMR, Admixed American; C_index, Harrel's C-index; CHARGE-AF, clinical risk score for atrial fibrillation; CHARGE, CHARGE-AF; CI.H.C, Confidence Interval Upper Bound; CI.L.C, Confidence Interval Lower Bound; EUR, European; -meta, derived from the AFGen+MVP meta-analysis; Mult-t, multi-trait; P-value, comparison with CHARGE-AF model using the one-sided Noether test.

Supplementary Data 21. AFR, African; ALL, all-ancestry; AMR, Admixed American; CI.H.HR, Confidence Interval Upper Bound; CI.L.HR, Confidence Interval Lower Bound; EUR, European; HARMS, clinical risk score (HARMS-AF) for atrial fibrillation; HARMS adj, adjusted for HARMS-AF; HR, Hazard Ratio; -meta, derived from the AFGen+MVP meta-analysis; Mult-t, multi-trait; P, p-value; Score, models using PGS alone (adjusted for the first 20 principal components (PCs), age and sex) or models including the PGS (each residualized for the first 20 PCs) with HARMS-AF included as a covariate, with the full model adjusted for the first 10 PCs; SE, standard error.

Supplementary Data 22. AFR, African; ALL, all-ancestry; AMR, Admixed American; C_index, Harrel's C-index; CI.H.C, Confidence Interval Upper Bound; CI.L.C, Confidence Interval Lower Bound; EUR, European; HARMS-AF, clinical risk score for atrial fibrillation; HARMS, HARMS-AF; -meta, derived from the AFGen+MVP meta-analysis; Mult-t, multi-trait; P-value, comparison with HARMS-AF model using the one-sided Noether test.

Supplementary Data 23. AFR, African; ALL, all-ancestry; AMR, Admixed American; CHARGE, clinical risk score (CHARGE-AF) for atrial fibrillation; EUR, European; HARMS, clinical risk score (HARMS-AF) for atrial fibrillation; -meta, derived from the AFGen+MVP meta-analysis; Model_1, first model included in one-sided Noether's test for significant differences; Model_2, second model included in one-sided Noether's test for significant differences; Mult-t, multi-trait.

Supplementary Data 24. AFR, African; ALL, all-ancestry; AMR, Admixed American; AUROC, Area Under the Receiver Operator Curve; AUPRC, Area Under the Precision Recall Curve; Beta, effect size; CI.H.OR/AUROC, Confidence Interval Upper Bound; CI.L.OR/AUROC, Confidence Interval Lower Bound; EAS, East Asian; EUR, European; .full, full model adjusted for age and sex, using PGS residualized for the first 20 principal components (PCs) of ancestry; liab_R2_delta_o_resid, Liability delta R^2 ($\Delta R^2 = R^2\_full - R^2\_base$) divided by the residual liability R^2 ($1 - R^2\_base$); -meta, derived from the AFGen+MVP meta-analysis; Mult-t, multi-trait; ngk_R2_delta_o_resid, Nagelkerke's delta R^2 divided by the residual Nagelkerke's R^2; OR, Odds Ratio; P, p-values calculated using two-sided Wald tests; PGS, Polygenic Score; .prs, univariate (PGS-only) model using PGS residualized for the first 20 PCs; SAS, South Asian; SE, standard error.

Supplementary Data 25. AUROC, Area Under the Receiver Operator Curve; AUPRC, Area Under the Precision Recall Curve; Beta, effect size; CI.H.OR/AUROC, Confidence Interval Upper Bound; CI.L.OR/AUROC, Confidence Interval Lower Bound; EUR, European; .full, full model adjusted for age and sex, using PGS residualized for the first 20 principal components (PCs) of ancestry; liab_R2_delta_o_resid, Liability delta R^2 divided by the residual liability R^2; Mult-t, multi-trait; ngk_R2_delta_o_resid, Nagelkerke's delta R^2 ($\Delta R^2 = R^2\_full - R^2\_base$) divided by the residual Nagelkerke's R^2 ($1 - R^2\_base$); OR, Odds Ratio; P, p-values

calculated using two-sided Wald tests; PGS, Polygenic Score; .prs, univariate (PGS-only) model using PGS residualized for the first 20 PCs; SE, standard error.

Supplementary Data 26. AFib, atrial fibrillation; AFlut, atrial flutter; OMOP.Id, OMOP concept ID; Pheno, phenotype.

Supplementary Data 27. AoU, All of Us

Supplementary Data 28. EUR, European; N, sample size.

Supplementary Data 29. BBJ, BioBank Japan; EAS, East Asian; Mult-t, multi-trait; PGS, Polygenic score; 3×, 4×, 5×, and ⅓× denote fold-changes in AF risk relative to the middle PGS quintile (%).

Supplementary Data 30. A fixed-effects inverse-variance-weighted meta-analysis of regression beta coefficients was performed; all reported p-values were calculated using two-sided Wald tests.
