# Supplementary material for: Multi-trait polygenic risk scores improve genomic prediction of atrial fibrillation across diverse ancestries
Source: Nat Commun. 2026 May 5;17:6059. doi: 10.1038/s41467-026-72708-x (PMC13350093; doi:10.1038/s41467-026-72708-x)
Supplement: Supplementary file 4 — Reporting Summary [file 41467_2026_72708_MOESM4_ESM.pdf]

nature portfolio

Corresponding author(s): Dr. S.J. Jurgens

Last updated by author(s): Oct 6, 2025

Reporting Summary

Nature Portfolio wishes to improve the reproducibility of the work that we publish. This form provides structure for consistency and transparency in reporting. For further information on Nature Portfolio policies, see our Editorial Policies and the Editorial Policy Checklist.

Statistics

For all statistical analyses, confirm that the following items are present in the figure legend, table legend, main text, or Methods section.

n/a	Confirmed	
☐	☒	The exact sample size (*n*) for each experimental group/condition, given as a discrete number and unit of measurement
☐	☒	A statement on whether measurements were taken from distinct samples or whether the same sample was measured repeatedly
☐	☒	The statistical test(s) used AND whether they are one- or two-sided<br>*Only common tests should be described solely by name; describe more complex techniques in the Methods section.*
☐	☒	A description of all covariates tested
☐	☒	A description of any assumptions or corrections, such as tests of normality and adjustment for multiple comparisons
☐	☒	A full description of the statistical parameters including central tendency (e.g. means) or other basic estimates (e.g. regression coefficient) AND variation (e.g. standard deviation) or associated estimates of uncertainty (e.g. confidence intervals)
☐	☒	For null hypothesis testing, the test statistic (e.g. *F*, *t*, *r*) with confidence intervals, effect sizes, degrees of freedom and *P* value noted<br>*Give P values as exact values whenever suitable.*
☐	☒	For Bayesian analysis, information on the choice of priors and Markov chain Monte Carlo settings
☒	☐	For hierarchical and complex designs, identification of the appropriate level for tests and full reporting of outcomes
☒	☐	Estimates of effect sizes (e.g. Cohen's *d*, Pearson's *r*), indicating how they were calculated

Our web collection on statistics for biologists contains articles on many of the points above.

Software and code

Policy information about availability of computer code

Data collection	No custom code was used for data collection since we used GWAS summary statistics and previously ascertained biobank data. All data pre-processing steps were described in the Supplementary Materials document.
Data analysis	Quality control was performed with various software tools as described in the Supplementary Materials, and mostly using PLINK2 (https://www.cog-genomics.org/plink/2.0/; various versions from May 2025 release onwards). A complete, reproducible workflow for generating the multi-trait polygenic scores, including all publicly available scripts, is provided at http://doi.org/10.5281/zenodo.19297684. This repository contains scripts for GWAS summary statistics meta-analyses of AF and SBP using METAL (release 2011-03-25; https://csg.sph.umich.edu/abecasis/metal/download/), polygenic score generation from all-ancestry AF summary statistics using SBayesRC (v0.2.6; https://github.com/zhilizheng/SBayesRC), allele alignment between polygenic score files and All of Us genotype data, scoring of PGS files per chromosome using PLINK2 (https://www.cog-genomics.org/plink/2.0/; various versions from February 2025 release onwards), merging of per-chromosome outputs, splitting the All of Us dataset into tuning and validation subsets, integrating multiple polygenic scores using the SBayesRC-multi tool and our adapted multi tool, both run in R v4.5.0, constructing the final combined polygenic score, and finally performing downstream analyses and extracting performance metrics. Performance metrics were extracted from the logistic regression models primarily using base R. AUROC was calculated with the pROC package, AUPRC with the PRROC package, and Nagelkerke's R^2 with the fmsb package. Liability R^2 was derived using a custom function based on formulas described in Lee et al. (2012). Analyses, data pre- and post- processing steps, and figure generation performed in R were run using versions 4.4.1–4.5.0.

For manuscripts utilizing custom algorithms or software that are central to the research but not yet described in published literature, software must be made available to editors and reviewers. We strongly encourage code deposition in a community repository (e.g. GitHub). See the Nature Portfolio guidelines for submitting code & software for further information.

Data

Policy information about availability of data

All manuscripts must include a data availability statement. This statement should provide the following information, where applicable:

- Accession codes, unique identifiers, or web links for publicly available datasets
- A description of any restrictions on data availability
- For clinical datasets or third party data, please ensure that the statement adheres to our policy

Data generated and processed in this study are provided as Source Data in the form of Supplementary Data. Detailed information on the GWAS training datasets, including article references and links to the data sources, is provided in Supplementary Data 6. The unrestricted MVP summary statistics used in this study are available in the dbGaP database without additional permissions under accession code phs002453.v1.p1 [https://www.ncbi.nlm.nih.gov/projects/gap/cgi-bin/study.cgi?study_id=phs002453.v1.p1]. Accordingly, the pan-ancestry and ancestry-specific GWAS summary statistics generated in this study from the AFGen and MVP meta-analysis have been deposited in the Cardiovascular Disease Knowledge Portal database [https://cvd.hugeamp.org/downloads.html]. These data can be used to reproduce the results shown in Supplementary Figures 26 and 27. The BBJ GWAS genotype data used in this study are available in the NBDC Human Database under accession code hum0014 [https://humandbs.dbcls.jp/en/hum0014]. The Roselli et al. all-ancestry PGS used in this study is available in the Cardiovascular Disease Knowledge Portal database [https://cvd.hugeamp.org/downloads.html#polygenic] and the PRSmix+ PGS for atrial fibrillation used in this study is available in the Polygenic Score Catalog database under accession code PGS004706 [https://www.pgscatalog.org/score/PGS004706]. The PGS generated in this study (five ancestry-tuned Mult-t PGSs, ALLmeta PGS, and the non-EUR PGSs excluding traits trained on UK Biobank data) have been deposited in the Cardiovascular Disease Knowledge Portal database [https://cvd.hugeamp.org/downloads.html] and in the Polygenic Score Catalog under accession codes PGS012531-12538 [https://www.pgscatalog.org/].

The raw phenotypic and genetic data from the All of Us Research Program and BioBank Japan are available under restricted access for protection of individual-level data due to data privacy laws. Access to both resources can be obtained by bona fide researchers with institutional data use agreements. For All of Us, access can be obtained through the Researcher Workbench, a cloud-based computing platform [https://www.researchallofus.org/register/]. Response to access requests is typically within a few days, and continued access requires completion of an annual training course and assessment. A publicly available data browser is also provided by the program [https://databrowser.researchallofus.org/]. For BioBank Japan, access can be obtained upon request [https://biobankjp.org/english/index.html]. Both biobanks restrict use to approved research purposes, prohibit re-identification and data sharing, and require secure data handling.

Research involving human participants, their data, or biological material

Policy information about studies with human participants or human data. See also policy information about sex, gender (identity/presentation), and sexual orientation and race, ethnicity and racism.

Reporting on sex and gender	We used the term sex rather than gender in this study and were careful not to conflate the two. In our study design, models were adjusted for sex, but the results were not specific to one sex. In the Discussion, we refer to the Supplementary Note, where we outline the future potential of sex-stratified polygenic scores. We did not perform such analyses in this study, as they would complicate the practical application of polygenic scores (requiring separate scores for each sex). Sex was genetically inferred (XX or XY) within the All of Us (AoU), BioBank Japan (BBJ) and external cohorts. We do not have consent to share individual-level data, including disaggregated sex data.
Reporting on race, ethnicity, or other socially relevant groupings	The ancestry samples used for the GWAS summary statistics were obtained either from the GWAS Catalog or from articles identified through Nature Advanced Search. Ancestry was genetically inferred using principal component analysis (PCA) and categorized as European, African, Admixed American, South Asian or East Asian groups. Participants in the All of Us, BioBank Japan and external cohorts were similarly labeled according to their genetically inferred ancestry (via PCA). We used these labels as they reflect broad genetic clusters based on shared allele frequencies, rather than cultural or geographical boundaries. To control for confounding, we adjusted our models for principal components of ancestry, as well as for sex and age.
Population characteristics	Population and clinical characteristics stratified by ancestry, case–control status, and tuning and validation sets are presented in Supplementary Data 1 for the All of Us dataset and in Supplementary Data 5 for the BioBank Japan dataset.
Recruitment	For AoU, samples were enrolled in a longitudinal cohort study (with aim of including 1 million racially, ancestrally and demographically diverse participants) from across the United States. Data is prospectively collected, combining phenotypic data from various sources including patient-derived information and electronic health record linkage. One of the goals set by AoU was to recruit individuals that have been and continue to be underrepresented in biomedical research because of limited access to health care.<br><br>For BBJ 2nd cohort, This is a prospective hospital-based national biobank project that collects DNA and serum samples and clinical information from 12 cooperative medical institutes throughout Japan. BBJ collected approximately 80,000 new patients with 38 target diseases collected between 2013 and 2018 to expand research outcomes from the first cohort. Atrial fibrillation or atrial flutter were determined by the physician's diagnosis or electrocardiogram records.<br><br>For the UKB, prospective participants were invited to visit an assessment centre, at which they completed an automated questionnaire and were interviewed about lifestyle, medical history and nutritional habits; basic variables such weight, height, blood pressure etc. were measured; and blood and urine samples were taken. These samples were preserved so that it was possible to later extract DNA and measure other biologically important substances. During the whole duration of the study it was intended that all disease events, drug prescriptions and deaths of the participants are recorded in a database, taking advantage of the centralized UK National Health Service.<br><br>For details on the HUNT and LOOP cohorts, please see in Supplementary Note 2 'The HUNT Cohort' and 'The LOOP Cohort'.
Ethics oversight	This study was conducted in accordance with all relevant regulations governing the use of human participants and was

performed in compliance with the Declaration of Helsinki. Informed consent was obtained from all participants across all cohorts used in this study. Access to the All of Us resource was granted by the All of Us Institutional Review Board, with analyses conducted under a data use agreement between Amsterdam UMC and the All of Us program. The BBJ project was approved by the ethics committees of the Institute of Medical Sciences at the University of Tokyo and the RIKEN Center for Integrative Medical Sciences. Use of UK Biobank resources was approved by the UK Biobank Research Ethics Committee, with UKB data accessed under approved application 176602. Use of the HUNT cohort was approved by the Regional Committee for Medical Research Ethics (2019/29771), the HUNT, the Norwegian Data Inspectorate, and by the National Directorate of Health. The study is in conformity with Norwegian laws and the Helsinki Declaration. The LOOP trial was approved by the local Ethics Committee and Data Protection Agency. Participant compensation varied by cohort and was determined by local ethics protocols, ranging from no financial compensation to reimbursement of expenses or in-kind participation benefits.

Note that full information on the approval of the study protocol must also be provided in the manuscript.

Field-specific reporting

Please select the one below that is the best fit for your research. If you are not sure, read the appropriate sections before making your selection.

☒ Life sciences ☐ Behavioural & social sciences ☐ Ecological, evolutionary & environmental sciences

For a reference copy of the document with all sections, see nature.com/documents/nr-reporting-summary-flat.pdf

Life sciences study design

All studies must disclose on these points even when the disclosure is negative.

Sample size

Sample sizes were based on the number of samples for which phenotypic and genetic (srWGS) data were available for the GWASs used to generate the summary statistics, as well as those available in All of Us, BioBank Japan, and the external cohorts. No power calculations were performed to pre-determine the required sample size. The training and validation datasets used in this study are among the largest and most ancestrally diverse available and are therefore considered sufficient.

Data exclusions

For the All of Us WGS dataset we used samples that passed quality controls, with high-quality genotypes, with multi-allelic variants already split into bi-allelic variants and variants with allele frequency (AF) = 0 removed, as described in the AoU Genomic Quality Report. We further filtered variants based on the following criteria: (1) monomorphic variants and (2) call rate < 90%. We excluded individuals flagged or identified as having known issues by AoU. We retained only samples with dragen_sex_ploidy equal to "XX" or "XY", excluded samples with genotype missingness > 5%, and removed potential duplicates. Duplicate resolution prioritized retaining individuals with linked electronic health record (EHR) data, followed by higher call rate (with greater weight given to EHR availability). Potential duplicates were identified using KING v2.3.2, with one sample from each pair flagged for removal if the heterozygous concordance exceeded 0.8.

For the BioBank Japan and external cohorts, we also used samples that passed quality controls, see in Supplementary Note 2 'The Biobank Japan cohort', 'The HUNT Cohort', 'The LOOP Cohort', and 'The UK Biobank Cohort' for a detailed explanation on this.

We used the BioBank Japan 2nd cohort and the HUNT4 cohort for validation of the polygenic scores, as other BioBank Japan and HUNT cohorts were partially included in the training data. For replication analyses in the UK Biobank, we excluded the traits height, body mass index, and dilated cardiomyopathy, because their training data included non-European UK Biobank participants. Finally, we validated the PRSmix+ score in the All of Us version 8 dataset, excluding all participants from version 6, as PRSmix+ was partially trained on those data.

Replication

To assess the robustness of our multi-trait polygenic scores (PGSs) across diverse populations, we included four datasets in addition to All of Us. First, we incorporated data from BioBank Japan to enable analyses in East Asian populations, where we observed strong predictive performance of the novel Mult-t-EAS score. We further evaluated replication in two external European cohorts: the Norwegian HUNT cohort and the Danish clinical LOOP cohort, the latter consisting of older individuals without atrial fibrillation but at elevated cardiovascular risk. Both cohorts demonstrated good performance of the Mult-t-EUR PGS. Finally, we assessed replication of the Mult-t-AFR and the newly developed Mult-t-SAS PGSs in the UK Biobank, observing comparable performance for Mult-t-AFR and moderately improved performance for Mult-t-SAS in this external dataset. Results are shown in Figure 5.

Randomization

Samples were not experimentally randomized, since the exposure in our analysis is genetic variation.

Blinding

In this study, we developed polygenic scores from GWAS summary statistics using advanced computational methods. As such, while blinding was not formally applied, the approach should not have been affected by the absence of a formal blinding procedure.

Reporting for specific materials, systems and methods

We require information from authors about some types of materials, experimental systems and methods used in many studies. Here, indicate whether each material, system or method listed is relevant to your study. If you are not sure if a list item applies to your research, read the appropriate section before selecting a response.

Materials & experimental systems

n/a	Involved in the study
☒	☐ Antibodies
☒	☐ Eukaryotic cell lines
☒	☐ Palaeontology and archaeology
☒	☐ Animals and other organisms
☒	☐ Clinical data
☒	☐ Dual use research of concern
☒	☐ Plants

Methods

n/a	Involved in the study
☒	☐ ChIP-seq
☒	☐ Flow cytometry
☒	☐ MRI-based neuroimaging

Plants

Seed stocks	n/a
Novel plant genotypes	n/a
Authentication	n/a
