# Supplementary material for: Multi-trait polygenic risk scores improve genomic prediction of atrial fibrillation across diverse ancestries
Source: Nat Commun. 2026 May 5;17:6059. doi: 10.1038/s41467-026-72708-x (PMC13350093; doi:10.1038/s41467-026-72708-x)
Supplement: Supplementary file 5 — Transparent Peer Review file [file 41467_2026_72708_MOESM5_ESM.pdf]

nature portfolio

Peer Review File

Multi-trait polygenic risk scores improve genomic prediction of atrial fibrillation across diverse ancestries

Corresponding Author: Dr Sean Jurgens

This file contains all reviewer reports in order by version, followed by all author rebuttals in order by version.

Version 0:

Reviewer comments:

Reviewer #1

(Remarks to the Author)
This study by Drs. Haydarlou, Bezzina, Jurgens and their colleagues takes a major step forward in PGS development for AF by utilizing a relatively new idea, which is incorporating the PGSs of related traits, to derive a PGS for AF that translates better across different genetic ancestries. It is well-written, and the data presentation is excellent, especially the demonstration of how different AF risk factors confer more or less risk in different populations. We have only a few comments:

1. For clinical implementation, don't you anticipate a common scenario will be that the actual measurements for height, BMI, and disease status for the traits that are included in the Mult-t score will be known? Therefore, how will the different PGS models perform compared to a clinical/genetic model that uses the actual data that is available for the traits? For implementation this probably depends a lot on the age of the individual you are considering because there is age-related penetrance of CAD, HF, DCM, and maybe BMI/obesity. Could you perform analyses to address this and present the results?

2. For selection of the seven additional traits for the multi-trait PGS development the authors state: "The traits were selected based on well described AF risk factors, high genetic correlation in Cardiovascular Disease Knowledge Portal, and availability of large-scale GWAS data." Could the authors be more explicit what correlation coefficient cut off from the Cardiovascular Disease Knowledge Portal was used? Also, what was considered large scale GWAS (number of participants in the GWAS, any SNP with GWS hit)? And how many traits from the clinically selected or selected from CVD portal were excluded, if any, due to lack of large scale GWAS data?

3. Regarding selection of cases, the authors included diagnostic codes for both AF and also AFL. One can wonder that although quite correlated, some patients might have AFL without AF (for example, a young patient with pulmonary HTN and RA dilatation). Could the authors provide breakdown of how many patients had only AFL as diagnosis. If a significant proportion (>5-10%), would the authors consider another sensitivity analysis with cases defined as AF codes only?

4. On page 22 under "data availability statement": Once made available, the accession ID and PGS catalog ID should be updated.

(Remarks on code availability)

Reviewer #2

(Remarks to the Author)
I co-reviewed this manuscript with one of the reviewers who provided the listed reports. This is part of the Nature Communications initiative to facilitate training in peer review and to provide appropriate recognition for Early Career Researchers who co-review manuscripts.

(Remarks on code availability)
The authors provide adequate code for reproducibility. There is a README file stat states what each script does and computing environment required. Furthermore the scripts names are also-self explanatory. The code also has comments, in

their majority, that explain what each snippet of code is doing.

METAL is a widely used meta-analysis tool from the center for statistical genetics. The code for multi are R functions that I could run locally without errors (albeit did not have example data frame).

My comments about code:

- Would be helpful for the authors to provide example or dummy data frames of each input file so can the readers know the format required for each step (mentioned sometime in the supplement but would be best if consolidated and shown here too). Alternatively can specify in the README what each input file should look like (column names and order if relevant and rows)

Reviewer #3

(Remarks to the Author)
The authors constructed atrial fibrillation (AF) polygenic risk scores (PRSs) applicable to various ethnic groups or to all populations using the SBayesRC framework. They incorporated the latest GWAS summary statistics for AF (e.g., AFGen and MVP) as well as for traits related to AF such as heart failure, BMI, and height. As a result, a multi-trait polygenic score (PGS)—which combines PGSs for AF and its related traits—demonstrated superior performance compared with previously reported PGSs. The performance was evaluated across multiple ethnic groups using samples from the All of Us cohort. Finally, the ability to identify individuals with high odds ratios for disease onset in the multi-trait EUR model was compared with Roselli et al.'s PGS, showing improved stratification of high-risk groups.
The methodology and results are well-structured and generally commendable. However, several aspects of the manuscript require clarification or improvement:

1. Please provide clinical background data (e.g., age, sex, comorbidities) from the All of Us cohort to clarify the population to which this PGS was applied.

2. The traits incorporated (HF, BMI, height, CAD, SBP, DCM, PR interval) are reasonable, but is their selection based on prior literature or objective criteria? Furthermore, could inclusion of additional traits further improve the performance of the multi-trait PGS?

3. In Fig. 3, the contribution of AF-related traits differs across ethnic groups. What exactly is being measured in the PGS context, and what epidemiological or molecular explanations might underlie these inter-ethnic differences?

4. When tuning the multi-trait PGS for each ethnic group, how would performance stability change if an N-fold cross-validation framework, similar to those used in machine learning, were employed?

5. Given the limited number of non-EUR participants for tuning and validation, is there any analytical verification demonstrating the robustness of the scores in these populations?

6. Beyond sample size, the relatively lower performance among African American and Admixed American populations might also reflect admixture differences compared with EUR and ASN. Could the authors provide additional discussion or analysis regarding this issue?

7. The manuscript notes that combining clinical risk scores such as HARMS2-AF or CHARGE-AF enhances stratification ability. Please clarify the combination method—was it a simple additive model, or were the components standardized and reweighted?

8. Although this study focuses on AF PRS, aside from Fig. 3 there is little justification for emphasizing AF specifically. Could this framework also enhance performance for other traits?

9. Related to the above point, aside from the improved stratification of high-risk individuals (Fig. 6), were there any findings that could be directly useful for AF clinical management?

10. To demonstrate the generalizability of the findings, it would be valuable to validate the results in an additional external cohort, at least within the EUR population.

(Remarks on code availability)
It contains concise scripts for running SBayesRC and METAL. It would be helpful if you could summarize the best pipeline for creating PGS (Polygenic Scores) as described in the actual paper.

Reviewer #4

(Remarks to the Author)
Haydarlou et al aimed to improve accuracy of AF risk prediction across ancestry groups. They applied the SBayesRC model, which incorporates functional annotation data, to the results from a new AF GWAS meta-analysis using data from the AFGen consortium (180 k cases) and the Million Veterans Program (88 k cases) and showed some improvement in AF

prediction compared to previous polygenic risk scores (PRS)(e.g. Roselli et al). The model incorporates functional annotation data, and was tuned by ancestry, using the AL of Us cohort. To further improve the polygenic risk score, the authors created a multi-ancestry polygenetic risk score and a multi-trait score. The multi-ancestry scores did not improve risk prediction notably, but the multi-trait did - particularly in the Asian populatoin. Risk prediction remains most accurate in the European population. The methods used are sound, well-described and seems reprodicible, and the manuscript is very well written, with nice figures. Adding other traits than AF in the prediction model is a novel and very interesting approach, which does improve risk prediction to some extent. However, when looking at the AUC, the improvements in prediction accuracy are only very modest, except perhaps for the simplified multi-t model applied to the Asian ancestry group. As seen in figure 2, the mult-t model increases the AUC by ~0.02-0.04 compared to the previous PRS from Roselli across ancestries.

Moreover, an AUC of 0.6 is not impressive when it comes to risk prediction in general, so it would also be interesting to see how the model performs adding clinical and other known risk factors.

The results from the novel GWAS meta-analysis of data from the AFGen cohort and the MVP cohort are not described at all the text or supplement and there are no tables or figures. The results would be very interesting to the reader - particularly any novel loci for AF - and should be added.

Many other efforts have been made to develop PRS for AF. The authors state in the introduction that PRSs face several major barriers that limit their clinical utility, for instance the lack of clinical use-cases and lack of reproducibility to diverse ancestry groups. The authors should be commended for aiming to build a PRS that performs better across ancestries, but it would be of importance to further assess how the mult-t model would perform in a clinical-use case, and I think that the manuscript would improve if that was added.

I would suggest to use the abbreviation PRS in stead of PGS to avoid confusion, since the latter is more widespread.

(Remarks on code availability)
The code for performing meta-analysis of GWAS, the code for creating the SBayesRC polygenic risk score, and the code for creating the multi-polygenic risk score is available at the github repository, together with a readme file explaining the use. All scripts look sound and have explanatory caption, which should make the results possible to reproduce.

Version 1:

Reviewer comments:

Reviewer #1

(Remarks to the Author)
All of my comments have been addressed thoroughly, and I think all of Reviewer 3’s comment have been addressed, too.

(Remarks on code availability)

Reviewer #2

(Remarks to the Author)
I co-reviewed this manuscript with one of the reviewers who provided the listed reports. This is part of the Nature Communications initiative to facilitate training in peer review and to provide appropriate recognition for Early Career Researchers who co-review manuscripts.

(Remarks on code availability)

Reviewer #4

(Remarks to the Author)
The authors have addressed my comments in a very good way, and the manuscript has improved substantively after the major revisions performed.
I would still have liked to see a more tangible clinical-use case beyond the replication of previous results from Vad et al in the Loop study. But I also agree that it may be beyond the scope of the current manuscript.

I only have a few minor comments:

1) In my previous comment 4.2, I suggested that the authors should evaluate the PGS performance compared with clinical risk models, which the authors have done. In the manuscript change on page 16, the authors state that "In terms of C-index, we found that the addition of ALLmeta or Mult-t scores to CHARGE-AF improved prediction of incident AF (Figure S21; Table S20). For instance, the C-index among EUR participants increased from 0.80 (95%CI [0.78; 0.82]) to 0.82 ([0.80; 0.84])

when adding Mult-t-EUR to the model, a significant difference by Noether's test (one-sided P=7.7x10-34; Supplementary Table S23). For AFR and AMR, significant (one-sided P=8.2x10-5) and suggestive (one-sided P=0.09) improvements were observed upon adding Mult-t-AFR and Mult-t-AMR, respectively." I would omit "suggestive" significance here, with a p-value of 0.09, which I would say is not significant. The differences in across ancestry groups in table S23 are interesting. The authors discuss the significance of improving genetic prediction of AF in underrepresented populations in the discussion section, but do not reflect on the fact that the new mult-t PGS adds relatively little to AF risk prediction beyond the clinical risk scores in the AMR and AFR populations, compared with EUR, as shown in figure S21 and Table S23. Can the authors speculate on the causes of the differences? If one takes into account the cost of genotyping, it may look like using a clinical risk score may be preferable or just as good as a PGS in certain population, especially in a cost-benefit view.

2) Would it be possible for the authors to incorporate p-values for comparison between models in their figures showing C-statistics? It would be helpful to see it directly in the figure instead of digging though supplemental material. E.g. in figure 2 – adding an asterisk denoting a significant improvement in predictive accuracy for the mult-t-EUR vs the Roselli PGS, etc.

(Remarks on code availability)

Open Access This Peer Review File is licensed under a Creative Commons Attribution 4.0 International License, which permits use, sharing, adaptation, distribution and reproduction in any medium or format, as long as you give appropriate credit to the original author(s) and the source, provide a link to the Creative Commons license, and indicate if changes were made.
In cases where reviewers are anonymous, credit should be given to 'Anonymous Referee' and the source.
The images or other third party material in this Peer Review File are included in the article's Creative Commons license, unless indicated otherwise in a credit line to the material. If material is not included in the article's Creative Commons license and your intended use is not permitted by statutory regulation or exceeds the permitted use, you will need to obtain permission directly from the copyright holder.
To view a copy of this license, visit https://creativecommons.org/licenses/by/4.0/

General remarks:

Dear Editors and Reviewers,

Thank you for the detailed evaluation of our manuscript. The revised manuscript contains a substantial amount of additional work, including the addition of several new cohorts. As such, we would like to first provide a clear overview of all major changes/additions, before we jump into the point-by-point responses. An overview of the new study design and cohorts is presented in the new **Figure 1**. Specifically, in terms of additional cohorts, we have:

- added data from BioBank Japan (~6k AF cases) to facilitate both PGS development and testing in an additional non-European cohort;
- added data from the HUNT4 cohort (341 cases) from Norway, to facilitate external validation of our novel Mult-t-EUR PGS;
- added data from the LOOP clinical trial (N=969 cases) from Denmark, to facilitate additional external validation and assess potential clinical utility of PGS for pre-screening.

In terms of analyses and presentation, we have:

- added exploratory analyses developing 'multi-method' PGS, which showed minimal added value at substantial additional computation cost;
- generated and validated multi-trait PGSes for East-Asian (EAS) and South-Asian (SAS) ancestries, separately;
- showcased the strong performance of Mult-t-EAS among EAS ancestry (comparable to EUR), adding visualizations/analyses to mirror Mult-t-EUR;
- added additional visualization and discussion regarding relative prediction improvements, showing that our multi-trait approach works particularly well in underrepresented ancestries with poor baseline prediction (new **Figure 3**);
- added several validation analyses, showing a general pattern of improved prediction of our novel PGSs, across datasets and ancestries (new **Figure 5**);
- added analyses pertaining to the joint predictive capacity of PGS alongside clinical risk models, highlighting their complementary nature;
- added analyses in the LOOP cohort, which suggest that individuals with high genetic risk benefit more from targeted AF screening to prevent strokes;
- added details and descriptions of our GWAS loci and prioritized genes.

Of course, based on these substantial additions/revisions, we have updated the Discussion accordingly.

Reviewer #1:

This study by Drs. Haydarlou, Bezzina, Jurgens and their colleagues takes a major step forward in PGS development for AF by utilizing a relatively new idea, which is incorporating the PGSs of related traits, to derive a PGS for AF that translates better across different genetic ancestries. It is well-written, and the data presentation is excellent, especially the demonstration of how different AF risk factors confer more or less risk in different populations. We have only a few comments:

Author response:
We would like to thank the Reviewer for the positive assessment of our work.

1.1) For clinical implementation, don't you anticipate a common scenario will be that the actual measurements for height, BMI, and disease status for the traits that are included in the Mult-t score will be known? Therefore, how will the different PGS models perform compared to a clinical/genetic model that uses the actual data that is available for the traits? For implementation this probably depends a lot on the age of the individual you are considering because there is age-related penetrance of CAD, HF, DCM, and maybe BMI/obesity. Could you perform analyses to address this and present the results?

Author response:
Thank you for this important point. As we see it, there are two important and complementary ways in which PGSes may be valuable. First, as was the focus of our work, PGSes may be used as isolated tools to estimate genetic risk. This is, for instance, relevant for the cardiovascular PGS tests emerging in the US[1]. The current goal of these PGS is to give the most accurate estimate of risk based solely on genetics; and to provide such estimates even at young age - when many correlated traits may indeed not yet be present in an individual. Other future scenarios can be envisioned where such PGS are relevant, too, such as population-based pre-screening (where clinical information may not be available/complete).

Second, as the Reviewer correctly mentions, PGSes are likely to be valuable in clinical settings alongside clinical risk indicators. We would agree that, in such a clinical setting, many of the actual measurements for AF-correlated traits (e.g., height, BMI, SBP, PR interval) as well as disease status (HF, CAD, DCM) will indeed be known. Nevertheless, PGS may capture inherited risk beyond what is measured noisily at a single time-point (e.g. for BMI or SBP) or may indeed capture risk that has not yet materialized into a penetrant phenotype (for diseases, or even for BMI/SBP). Therefore, as requested by the Reviewer, we set out to assess the joint predictive capacity of established clinical risk tools and our novel PGSes.

To address this, we performed additional analyses in *All of Us* focussed on incident AF events, where we (1) compared the hazard ratios (HRs) of our PGS before and after adjustment for the CHARGE-AF and HARMS2-AF clinical scores, and (2) compared the

C-indices of clinical-only models to prediction models that combined the clinical scores with PGS. These analyses were performed in three ancestry groups with sufficient events for incident analyses (EUR, AFR, AMR). Across ancestries, we found that the ALLmeta and Mult-t PGSes remained significantly associated with incident AF risk, even after adjusting for either clinical model. While we found that effect sizes were a bit more attenuated for the multi-trait PGSes, the overall HRs remained larger for the multi-trait score, even after adjustment. In terms of C-index, we observed that prediction significantly improved in EUR and AFR upon addition of ALLmeta (suggestive in AMR), with further significant improvements with the multi-trait PGSes (in EUR and AFR). Results for the CHARGE-AF risk model are presented below (**Figure R1-R2**); results for both risk models are also presented in new **Supplementary Figures 20-23** and **Supplementary Tables 19-23**. Overall, these results show that the multi-trait PGSes do not hurt prediction in the setting of established clinical risk models, and even suggest that they capture some additional risk beyond the ALLmeta PGS in this setting.

That being said, we find the suggestion of Reviewer #3 very interesting in this setting: Novel multi-trait PGS can be derived that are optimized for utility alongside either clinical risk model. While we are very interested in this approach, we worry that it might be out of scope for the current manuscript. Of course, if the Reviewer and Editors feel strongly, we can explore this further.

Manuscript change:
Page 16:
Finally, we assessed whether our novel PGSs improve prediction of AF on top of existing clinical risk prediction tools. Within the All of Us validation sets, focussing on incident AF, we found that both the ALLmeta and Mult-t scores remained strongly associated with AF when conditioned on the CHARGE-AF clinical model[30], across EUR, AMR and AFR ancestries (Figure S20 and S21; Table S19 and S20). We found that Hazard ratios for the Mult-t scores were more attenuated than for ALLmeta upon conditioning for CHARGE-AF; nevertheless, Mult-t scores still yielded higher Hazard ratios than ALLmeta across the three ancestry groups, even after conditioning (Figure S20; Table S19). Similar patterns were observed for the HARMS2-AF clinical risk score[31] (Figure S22 and S23; Table S21 and S22).
In terms of C-index, we found that the addition of ALLmeta or Mult-t scores to CHARGE-AF improved prediction of incident AF (Figure S21; Table S20). For instance, the C-index among EUR participants increased from 0.80 (95%CI [0.78; 0.82]) to 0.82 ([0.80; 0.84]) when adding Mult-t-EUR to the model, a significant difference by Noether's test (one-sided P=7.7x10-34; Supplementary Table S23). For AFR and AMR, significant (one-sided P=8.2x10-5) and suggestive (one-sided P=0.09) improvements were observed upon adding Mult-t-AFR and Mult-t-AMR, respectively. The Mult-t scores also improved over ALLmeta PGS in models alongside CHARGE-AF (one-sided P<0.05 in EUR and AFR). Similar patterns were observed for the HARMS2-AF clinical score (Supplementary Table S23). These findings indicate that our novel PGSs represent independent risk indicators that improve discrimination of AF alongside clinical risk models. Future work should now assess whether clinical model-informed reweighting of PGS components may improve prediction further.

Pages 25-26:
Finally, we assessed whether our novel PGS would improve AF prediction on top of existing clinical primary risk prediction models. To this end, we assessed EUR, AMR and AFR ancestry groups in the All of Us dataset, assessing the utility of our Mult-t scores (mult-t-EUR, mult-t-AMR, mult-t-AFR) alongside both the CHARGE-AF and HARMS2-AF clinical scores[30,31], using Cox time-to-event

regression models, testing for incident AF events (events after enrollment to All of Us). Models including the PGS as the sole predictor were adjusted for age, sex, and the first 20 principal components (PCs). Models including a clinical risk score alone, as well as models jointly including a clinical risk score and a PGS (residualized for the first 20 PCs), were adjusted for the first 10 PCs. The phenotypic components used to derive the CHARGE-AF and HARMS2-AF clinical risk scores are described in detail in Table S27. In particular, we assessed (i) the change in hazard ratios for PGS upon adjusting for either clinical risk model, and (ii) the change in model C-statistic upon addition of PGS to either clinical risk model.

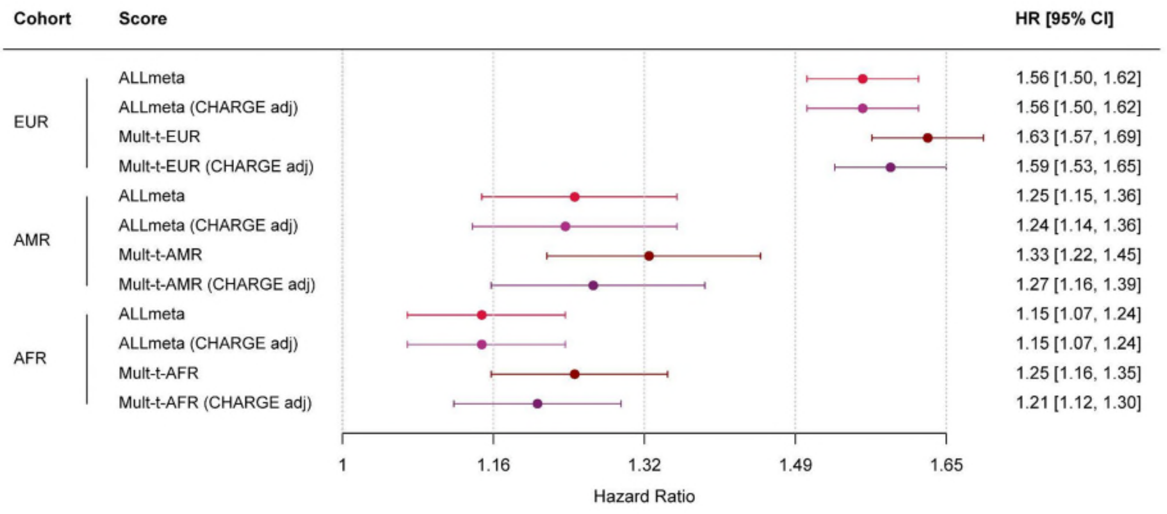

Figure R1 | Figure R1: Hazard ratios for incident AF prediction for ALLmeta and Mult-t PGS before and after adjusting for the CHARGE-AF clinical model. The left panel displays a forest plot of hazard ratio with 95% CIs on the x-axis. The y-axis lists the ancestry-specific validation cohorts, each paired with its respective scores. The right panel displays the corresponding hazard ratio values numerically. The CHARGE-AF score is a clinical risk score for AF derived from multiple phenotypes (Table S24). Estimates are based on cox regression models using PGS alone (adjusted for the first 20 PCs, age and sex) or models including the ALLmeta or multi-trait PGSs (each residualized for the first 20 PCs) with CHARGE-AF included as a covariate, with the full model adjusted for the first 10 PCs. Case/control counts for incident AF and model performance details in All of Us are provided (Table S19)

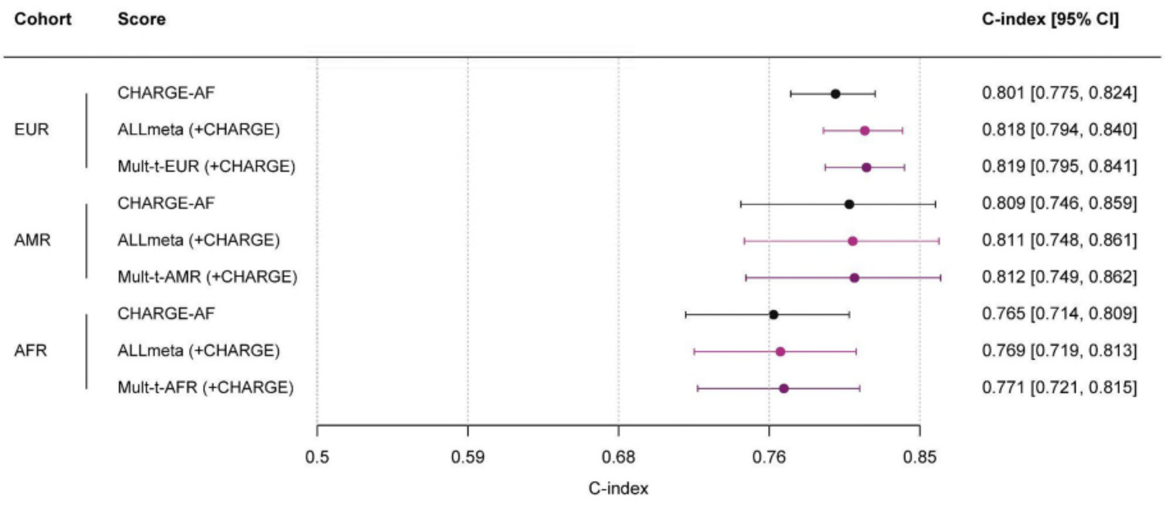

Figure R2 | Figure R1: C-indices for incident AF prediction for the CHARGE-AF clinical model alone, and combined with ALLmeta and Mult-t PGSes. The left panel displays a forest plot of Harrell's C-index with 95% CIs on the x-axis. The y-axis lists the ancestry-specific validation cohorts, each paired with its respective scores. The right panel displays the corresponding C-index values numerically. The CHARGE-AF score is a clinical risk score for atrial fibrillation derived from multiple phenotypes (Table S27). Estimates are based on cox regression models using CHARGE-AF alone (adjusted for the first 10 PCs) or models including the ALLmeta or multi-trait PGSs (each residualized for the first 20 PCs) with CHARGE-AF included as a covariate, with

the full model adjusted for the first 10 PCs. Case/control counts for incident AF and model performance details in All of Us are provided (Table S20).

1.2) For selection of the seven additional traits for the multi-trait PGS development the authors state: “The traits were selected based on well described AF risk factors, high genetic correlation in Cardiovascular Disease Knowledge Portal, and availability of large-scale GWAS data.” Could the authors be more explicit what correlation coefficient cut off from the Cardiovascular Disease Knowledge Portal was used? Also, what was considered large scale GWAS (number of participants in the GWAS, any SNP with GWS hit)? And how many traits from the clinically selected or selected from CVD portal were excluded, if any, due to lack of large scale GWAS data?

Author response:
This is an understandable comment made by the Reviewer. We did not apply a pre-defined pipeline and/or pre-defined criteria to identify the correlated traits. Instead, we started with traits that showed significant and strong genetic correlation with AF based on LD score regression in previous studies[2,3], for which we knew that well-powered GWAS (millions of participants) were available (including HF, BMI, height, CAD, blood pressure, PR interval).

We then used the Cardiovascular Disease Knowledge Portal to identify potential additional traits of relevance to AF (filtering to rg>0.3). Except for DCM, these were all manually discarded either due to:
- The absence of large well-powered GWAS data (e.g. sinus node dysfunction, distal conduction disease, proBNP, pulmonary hypertension, chronic pulmonary heart disease; for which GWAS included only a few thousand cases).
- Substantial overlap with the traits already included (e.g., HFpEF/HFrEF/congestive HF with heart failure/cardiomyopathy).

We have amended the language of the methods to make the approach more transparent, and to make more clear that the approach was not formally systematic.

Manuscript change:
Page 23-24:
The traits were manually selected based on having strong genetic correlation to AF[70,71], and there being large-scale GWAS data available for those traits (HF, BMI, height, CAD, SBP, PR-interval). We also scanned the Cardiovascular Disease Knowledge Portal[72] to identify additional traits with genetic correlation (rg>0.3) with AF; DCM was additionally included, while many other traits were pragmatically excluded (due to a high similarity to traits already included, or due to small size of the available GWAS). While we acknowledge that trait selection was not systematic, we posit that the final selection is a good representation of important AF-comorbidities and risk factors.

1.3) Regarding selection of cases, the authors included diagnostic codes for both AF and also AFL. One can wonder that although quite correlated, some patients might have AFL without AF (for example, a young patient with pulmonary HTN and RA dilatation). Could the authors provide a breakdown of how many patients had only AFL as diagnosis. If a

significant proportion (>5-10%), would the authors consider another sensitivity analysis with cases defined as AF codes only?

Author response:
Great point. If a substantial proportion of cases had only atrial flutter (AFL) diagnoses, performing a sensitivity analysis would indeed be appropriate to assess the robustness of our results. We examined this and found that, among the 21,942 total AFib/AFlut cases, only 631 (2.9%) had an AFL-only diagnosis. Because this proportion is small (<5-10%), we decided not to conduct a sensitivity analysis restricted to AF-only cases.

1.4) On page 22 under “data availability statement”: Once made available, the accession ID and PGS catalog ID should be updated.

Author response:
Thank you for the suggestion. We will update the Data Availability Statement with the accession ID and PGS Catalog ID as soon as they become available.

Reviewer #2:

2.1) Remarks on code availability: The authors provide adequate code for reproducibility. There is a README file that states what each script does and computing environment required. Furthermore the scripts names are also-self explanatory. The code also has comments, in their majority, that explain what each snippet of code is doing. METAL is a widely used meta-analysis tool from the center for statistical genetics. The code for multi are R functions that I could run locally without errors (albeit did not have example data frame).

Author response:
Thank you for the positive feedback regarding our code availability and documentation. We appreciate your careful review of the scripts and confirmation that the R functions and METAL workflow run as expected.

2.2) Comments about code: Would be helpful for the authors to provide example or dummy data frames of each input file so can the readers know the format required for each step (mentioned sometime in the supplement but would be best if consolidated and shown here too). Alternatively can specify in the README what each input file should look like (column names and order if relevant and rows).

Author response:
Thank you for the suggestion. The input file format requirements for both the SBayesRC-multi and adapted Multi tools are already described at the beginning of their respective scripts (SBRCmulti.R and Multi_tool.R). However, we agree that consolidating this information in the README would improve clarity for users. Therefore, we have expanded the README and added dummy data to the GitHub.

Reviewer #3:

The authors constructed atrial fibrillation (AF) polygenic risk scores (PRSs) applicable to various ethnic groups or to all populations using the SBayesRC framework. They incorporated the latest GWAS summary statistics for AF (e.g., AFGen and MVP) as well as for traits related to AF such as heart failure, BMI, and height. As a result, a multi-trait polygenic score (PGS)—which combines PGSs for AF and its related traits—demonstrated superior performance compared with previously reported PGSs. The performance was evaluated across multiple ethnic groups using samples from the All of Us cohort. Finally, the ability to identify individuals with high odds ratios for disease onset in the multi-trait EUR model was compared with Roselli et al.'s PGS, showing improved stratification of high-risk groups.
The methodology and results are well-structured and generally commendable. However, several aspects of the manuscript require clarification or improvement:

Author response:
We are grateful to the Reviewer for their detailed review of our work.

3.1) Please provide clinical background data (e.g., age, sex, comorbidities) from the All of Us cohort to clarify the population to which this PGS was applied.

Author response:
As suggested, we have added a baseline table providing clinical background data, including age, sex, and comorbidities, to clarify the characteristics of the *All of Us* cohort in which the PGS was developed and applied. This is available in new **Table S1**. We have also added such a table for the second major cohort, Biobank Japan (added during revision; **Table S5**).

3.2) The traits incorporated (HF, BMI, height, CAD, SBP, DCM, PR interval) are reasonable, but is their selection based on prior literature or objective criteria? Furthermore, could inclusion of additional traits further improve the performance of the multi-trait PGS?

Author response:
Thank you. The rationale for trait selection is described in our response to comment 1.2:
We did not apply a pre-defined pipeline and/or pre-defined criteria to identify the correlated traits. Instead, we started with traits that showed significant and strong genetic correlation with AF based on LD score regression in previous studies[2,32,3] (including HF, BMI, height, CAD, blood pressure, PR interval). We then scanned the Cardiovascular Disease Knowledge portal to identify additional potential traits, keeping only DCM (based on similarity of other traits to those already included, and based on expected low power due to relatively small sample sizes). As mentioned in comment 1.2, we have amended the language to more transparently describe that this process was not formally systematic.

As the Reviewer mentions, adding additional genetically correlated traits with sufficiently powered GWAS could further improve the multi-trait PGS. However, there is a point of diminishing returns: once major shared genetic components are captured, additional traits may contribute more estimation noise than useful signal, especially when mixing weights are obtained from standard logistic regression rather than a penalized approach. Evaluating this systematically—for example, by incrementally adding candidate traits with cross-validation to identify where performance plateaus—would be a valuable extension for future work.

Manuscript change:
Pages 23-24:
The traits were manually selected based on having strong genetic correlation to AF[70,71], and there being large-scale GWAS data available for those traits (HF, BMI, height, CAD, SBP, PR-interval). We also scanned the Cardiovascular Disease Knowledge Portal[72] to identify additional traits with genetic correlation (rg>0.3) with AF; DCM was additionally included, while many other traits were pragmatically excluded (due to a high similarity to traits already included, or due to small size of the available GWAS). While we acknowledge that trait selection was not systematic, we posit that the final selection is a good representation of important AF-comorbidities and risk factors.

3.3) In Fig. 3, the contribution of AF-related traits differs across ethnic groups. What exactly is being measured in the PGS context, and what epidemiological or molecular explanations might underlie these inter-ethnic differences?

Author response:
In **Figure 3**, the mixing weights measure how much predictive information each trait-specific PGS contributes to the final multi-trait score within a given ancestry. This reflects (i) the association of the PGS with AF independent of effects already captured by other PGSes in the model, and (ii) the variability of that trait-specific PGS within that ancestry. Larger weights therefore indicate stronger predictive value for AF, conditional on the other PGSes, in that ancestry's tuning set.

The inter-ancestral differences in the mixing weights likely arise from several factors.
<u>Epidemiological factors:</u> Differences in trait prevalence, and differences in gene–environment interactions, across populations may modify how strongly certain correlated traits contribute to AF risk. In this case, actual etiology, and therefore the contribution of different biological pathways, may differ between ancestries. Indeed, different mixing weight may then be representative of differences in AF etiology/pathways.
<u>Statistical factors:</u> Even if the actual biological pathways *are* shared across ancestries, differences in genetic architecture (e.g., different causal variants, different LD structures) may impact on variant effect modelling and PGS performance. *Noise:* Variant effect modelling therefore becomes increasingly noisy as the genetic distance between base GWAS and target population increases. Addition of more PGSes - which capture the shared biological pathways in different ways - may then help stabilize (reduce the noise) of variant effects. Which correlated traits help more in certain groups is then determined by ancestry-specific genetic correlations, and by stochastics. *Bias:* Mismatch between training data (GWAS) and target data also causes a downward bias in prediction accuracy.

A more simple statistical explanation may be that the GWAS data from certain correlated traits are more representative of the genetic ancestry of the target data; these traits will then also attain higher mixing weights. These statistical points are discussed in more detail in the Supplementary Note ("Technical aspects of our PGS methodology", point 4).

Supplementary Note change:

Pages 4-6:

Fourth, the relative benefit of the multi-trait approach differed across ancestries, with the largest gains observed in populations where baseline prediction was poor (e.g. AFR, AMR, and SAS). Comparatively, small gains were observed in EUR and EAS where baseline prediction was already relatively strong.

One possible explanation lies in differences in linkage disequilibrium (LD) patterns across populations. Single-trait PGSs trained predominantly on specific ancestry data (e.g. EUR) may transfer poorly to other ancestries due to LD mismatches[8]. In contrast, multi-trait models may be more robust across ancestries by leveraging pleiotropic variants that are less sensitive to population-specific LD structure, and by reducing noise in effect size estimation. Consequently, the AFR, AMR and SAS populations—where LD mismatch and effect size noise are more pronounced—derive greater benefit from the multi-trait framework, whereas EUR and EAS individuals, whose genetic structure is more closely-captured by existing large-scale GWAS data, experience more limited incremental gains. Supporting this interpretation, prior work on hematological traits has demonstrated high cross-ancestry consistency of multi-trait genetic effect sizes[9], providing a biological rationale for the enhanced transferability of multi-trait models.

Further insight into the ancestry-dependent performance of the Mult-t models was obtained by decomposing the ancestry-specific mixing weights across traits. We observed substantial heterogeneity in these weights between tuning populations, indicating that trait-specific PGSs capture ancestry-dependent signals beyond those contained in the AF-only score. Notably, the contribution of a given trait within the multivariate model did not necessarily correspond to its standalone predictive performance, suggesting that the multi-trait framework prioritizes complementary information rather than maximizing marginal effects. Some discrepancies may also reflect differences between tuning and validation datasets.

Inter-ancestral differences in the estimated mixing weights likely arise from a combination of epidemiological, statistical, and data-related factors. Epidemiologically, differences in trait prevalence and gene–environment interactions across populations may lead to ancestry-specific variation in AF etiology, such that distinct biological pathways contribute differentially to disease risk. In this context, ancestry-specific mixing weights may reflect genuine differences in underlying disease mechanisms. Statistically, even when biological pathways are shared, differences in genetic architecture—including causal variants and LD structure—affect effect size estimation and PGS performance (as mentioned above). As genetic distance between discovery GWAS and target populations increases, variant effect estimates become noisier; integrating multiple correlated PGSs may therefore stabilize prediction by capturing shared biology through partially independent genetic signals. Which traits contribute most strongly within each ancestry is then influenced by ancestry-specific genetic correlations as well as stochastic variation. Finally, bias introduced by mismatches between discovery and target populations can reduce predictive accuracy overall. In this setting, correlated traits whose GWAS data better represent the ancestry of the target population may naturally receive higher mixing weights.

3.4) When tuning the multi-trait PGS for each ethnic group, how would performance stability change if an N-fold cross-validation framework, similar to those used in machine learning, were employed?

Author response:

We thank the reviewer for this valuable suggestion. To assess performance stability in a cross-validation–like framework, we implemented a two-fold tuning approach to estimate mixing weights using the adapted Multi tool. Specifically, the original 70% testing set was split into two equal parts, and mixing weights were independently derived in each subset (‘Split 1’ and ‘Split 2’). These weights were then compared with those obtained from the original 30% tuning set. Across ancestral groups, the contribution of AF PGS to the multi-trait PGS was fairly stable across splits, with only modest variation in the weights of correlated traits (**Figure R3**). Such variability is expected, as these traits are themselves correlated (e.g. heart failure (HF), body mass index (BMI), and coronary artery disease (CAD)), allowing partial redistribution of weights. Overall, these results show that our approach yields relatively stable mixing weights, and suggest that a N-fold cross validation approach would not have much added value (with the current number of variables).

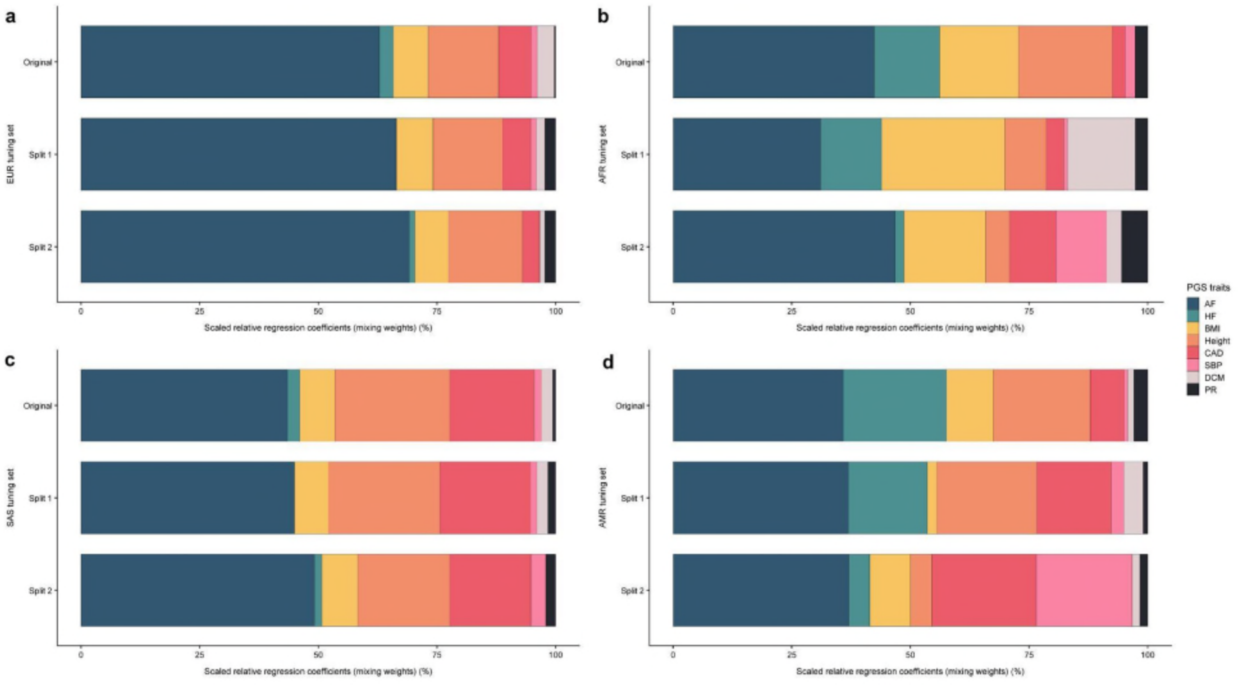

Figure R3 | Sensitivity analysis of mixing weights in a halved 70% testing set. Each panel shows the relative contribution of eight traits (legend) to the final multi-trait PGS. The x-axis represents mixing weights, defined as scaled regression coefficients expressed as percentages and normalized to sum to 100%, thereby reflecting the trait composition of the Mult-t scores. Weights were derived using the adapted Multi tool, which estimates trait-specific weights from unadjusted logistic regression models based on each trait’s predictive accuracy for AF. Weights were estimated in the original 30% tuning set (‘Original’; y-axis) and independently in the two halves of the 70% testing set (‘Split 1’ and ‘Split 2’; y-axis) to assess performance stability. Panels correspond to analyses in **(a)** European, **(b)** African, **(c)** South Asian, and **(d)** Admixed American individuals.

3.5) Given the limited number of non-EUR participants for tuning and validation, is there any analytical verification demonstrating the robustness of the scores in these populations?

Author response:

With respect to robustness of our multi-trait approach, we posit that the stability of our mixing weights (as shown in **Figure R3**) provides analytical reassurance.

With respect to the predictive capacity of our novel PGSes, we have substantially expanded our replication data. We now include 4 datasets other than *All of Us*. First, we added data from Biobank Japan to allow analyses of EAS; across validation sets in Biobank Japan and *All of Us*, we found very high prediction metrics for our novel Mult-t-EAS score, as shown in **Figure R4** (**Figure 2** in manuscript) and in **Figure R5** (new **Figure 5** in manuscript). We also added corrected replication data for Mult-t-AFR and our new Mult-t-SAS PGS in UK Biobank, showing similar (Mult-t-AFR) and somewhat better (Mult-t-SAS) prediction in this external dataset. Finally, we added replication data for two additional external EUR cohorts, which also demonstrated good performance of our Mult-t-EUR PGS. Together, these results indicate robustness of our multi-trait PGSs across diverse populations, although additional replication for Admixed-American ancestry is still needed in future work.

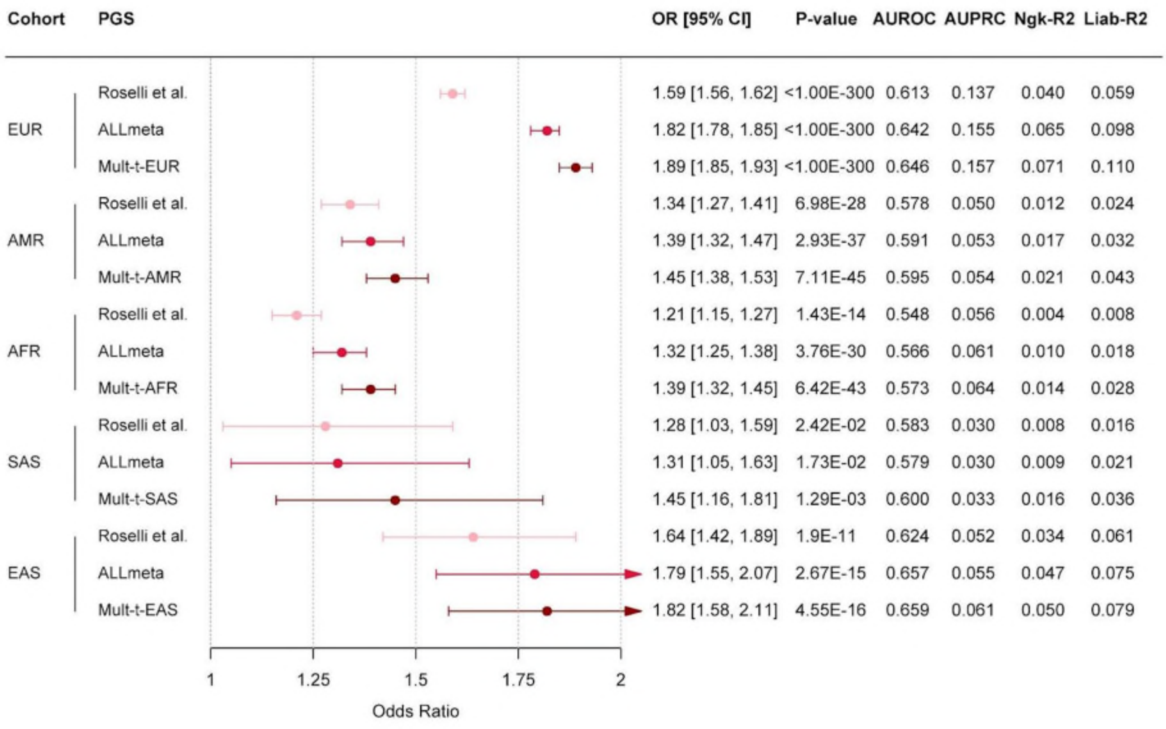

Figure R4 | Comparison of the Roselli et al., ALLmeta, and Mult-t PGSs for AF prediction in *All of Us*. The left panel displays a forest plot of the OR/SD increase in PGS for AF, with 95% CIs on the x-axis. The y-axis lists the ancestry-specific validation cohorts, each paired with its respective PGSs. The right panel summarizes key performance metrics from the validation set. All estimates are based on logistic regression models adjusted for the first 20 PCs, age, and sex, using case/control counts from the 70% validation dataset of *All of Us* (**Table S1**). Due to limited sample size, the full 100% validation set was used for SAS and EAS ancestry. Model performance details are provided (**Table S7**).

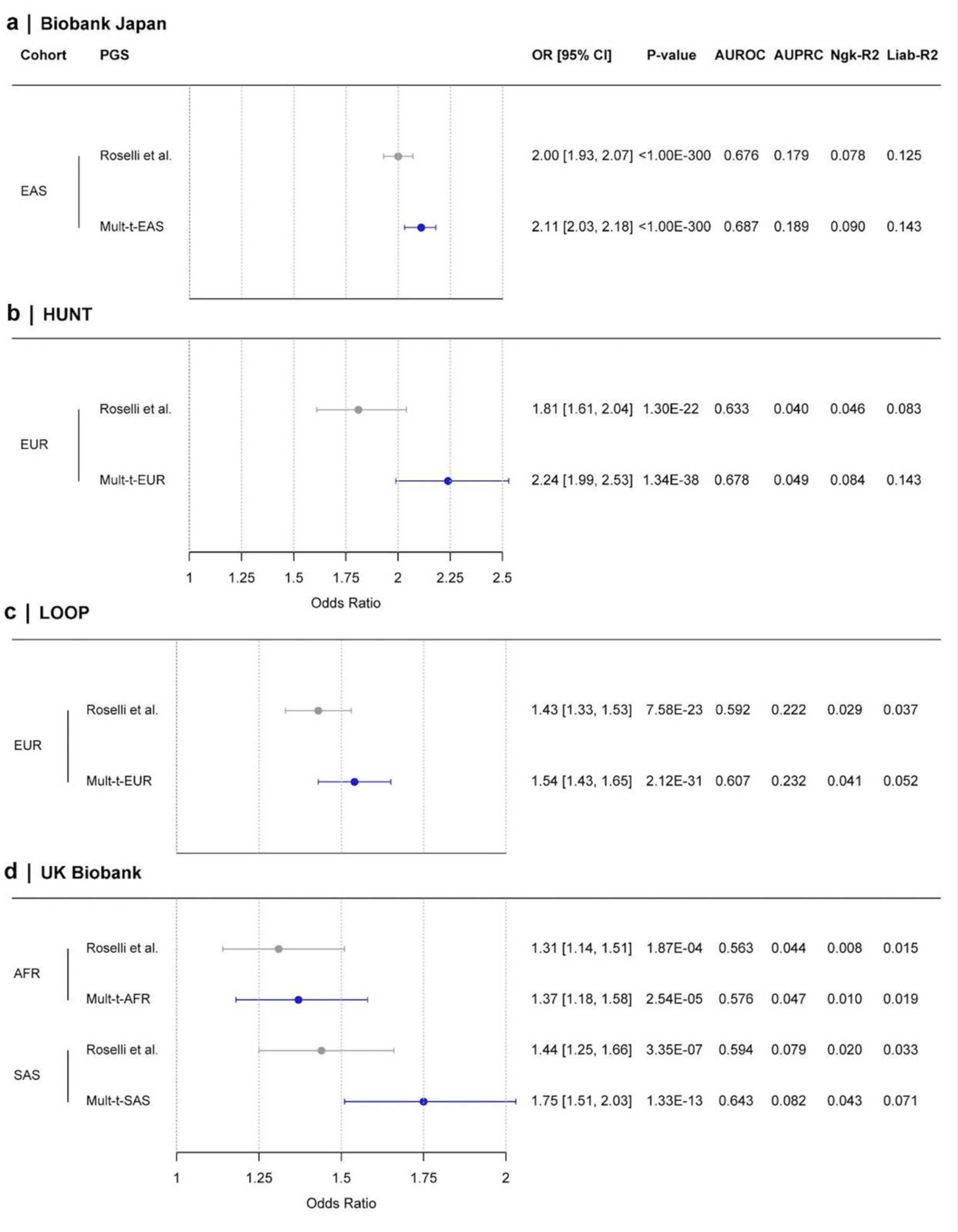

Figure R5 | Comparison of the Roselli et al. and Mult-t polygenic scores for AF prediction across four additional datasets. In each panel, the left part displays a forest plot of the OR/SD increased risk of AF, with 95% CIs, on the x-axis. The y-axis displays the respective ancestry groups tested, as well as the specific PGSs assessed. The right part summarizes key performance metrics. All estimates are based on logistic regression models adjusted for relevant covariates, including ancestry PCs, age and sex. Panel **a** shows results from EAS ancestry samples within Biobank Japan; panels **b** and **c** show results for EUR ancestry samples from HUNT and LOOP, respectively; panel **d** shows results from AFR and SAS ancestry from UK Biobank. In panel **d**, PGSs are based on simplified forms to prevent overfitting (**Methods**).

Manuscript change:
Pages 11-13:
In the validation set of BioBank Japan (Figure 5a), we observed a strong performance of the Mult-t-EAS score for prediction of AF (OR/SD 2.11; AUROC 0.687), which improved over the Roselli et al. score (ΔOR/SD = 0.10; ΔAUROC = 0.011; ΔAUPRC = 0.010). Mult-t-EAS only minimally improved over ALLmeta, likely because this PGS already provided strong performance in this dataset (Figure S13).

We also validated our Mult-t-EUR score in external cohorts (Figure 1). In the Norwegian HUNT4 cohort, a population-based cohort from Norway[27], we observed a stepwise improvement in predictive performance from the Roselli score to the Mult-t-EUR score, which demonstrated substantial performance for AF prediction in this cohort (OR/SD = 2.24; AUROC = 0.678) (Figure 5b; Table S14).

We also assessed the Mult-t-EUR score in the LOOP study, a Danish clinical trial assessing implantableloop recorder (ILR) screening in older participants without AF but at elevated cardiovascular risk[28]. In this high-risk population, the Mult-t-EUR score strongly outperformed the Roselli score (OR/SD = 1.54; AUROC = 0.607; Figure 5c; Table S15). Furthermore, recapitulating recent findings[29], we found that ILR screening was associated with lower rates of stroke or systemic embolism in participants with higher Mult-t-EUR PGS (HR = 0.66, P=0.048), with no benefit in participants with lower Mult-t-EUR PGS (HR = 1.00, P=0.98) (Figure S15).

We then aimed to evaluate the performance of Mult-t-AFR and Mult-t-SAS in the UK Biobank. Given that non-European UK Biobank participants contributed to some of the PGSs incorporated into our Mult-t scores, we assessed simplified scores that excluded these specific PGSs (Methods). Among individuals of AFR and SAS ancestry in UK Biobank, the simplified Mult-t scores outperformed the Roselli et al. score in AFR and SAS ancestry individuals, respectively. The most pronounced improvement was observed in SAS ancestry (ΔOR/SD = 0.31; ΔAUROC = 0.049; ΔAUPRC = 0.003). In AFR, performance was also nominally improved (ΔOR/SD = 0.06; ΔAUROC = 0.013; ΔAUPRC = 0.003) compared with the Roselli et al. PGS (Figure 5d; Table S16).

3.6) Beyond sample size, the relatively lower performance among African American and Admixed American populations might also reflect admixture differences compared with EUR and ASN. Could the authors provide additional discussion or analysis regarding this issue?

Author response:
We thank the reviewer for this comment. We acknowledge that differences in admixture, including varying ancestry proportions and distinct LD patterns, could have contributed to the lower performance of the Mult-t PGS in African American and Admixed American populations. We have added a sentence to the Discussion highlighting this as an additional potential explanation for the observed differences in predictive performance.

Manuscript change:
Page 19:
Nevertheless, prediction in AFR lags behind SAS and AMR despite larger GWAS case numbers, a finding potentially due to the greater genetic diversity and shorter haplotype blocks seen in Africans[57]. Differences in European admixture may also affect AFR and AMR ancestries specifically[53,54]. In any case, similarly reduced performance in AFR and AMR groups has been reported in two CAD PGS studies[19,42].

3.7) The manuscript notes that combining clinical risk scores such as HARMS2-AF or CHARGE-AF enhances stratification ability. Please clarify the combination method—was it a simple additive model, or were the components standardized and reweighted?

Author response:
We believe the reviewer refers to the following statement in the previous version of the Discussion:
"In recent work, an AF PGS was shown to enhance risk prediction when added to clinical models such as HARMS2-AF (AUROC: 0.828 to 0.839) and CHARGE-AF (0.808 to 0.828) in population-based cohorts like UK Biobank[4], and in a clinical cohort of cardiovascular disease patients, where combining an AF PGS with CHARGE-AF and NT-proBNP improved the C-index from 0.67 to 0.70[5]."

Regarding the combination method in these studies:
- Reference 4 performed model reweighting. In that study, the PGS was first converted into categorical variables (low, intermediate, high). These categories were then incorporated alongside the clinical CHARGE-AF or HARMS2-AF predictors in an elastic-net regression framework (5-fold cross-validation in a derivation set), effectively re-estimating the weights of both the clinical and genetic predictors.
- Reference 5 appears to have used a simple additive approach: the clinical risk score (CHARGE-AF) and the PGS were included together in regression models. No standardization or reweighting of the individual components was reported.

We agree with the Reviewer that the addition of PGS to clinical models is of importance to the field. Therefore, we have now added substantial analyses assessing the joint predictive capacity of clinical risk models and our novel PGSes. As also mentioned in comment 1.1: In a simple combination approach, we found that PGSes significantly improved prediction over the CHARGE-AF and HARMS2-AF scores in most approaches/ancestries. Our Mult-t PGSes also provided improvements over the ALLmeta PGS in this setting, in EUR and AFR. Results for the CHARGE-AF risk model are presented in **Figure R1-R2**; results for both risk models are also presented in new **Supplementary Figures 20-23** and **Supplementary Tables 19-23**.

Finally, we find the suggestion of the Reviewer to reweight score components in this setting quite intriguing. However, we worry that this is perhaps out of scope for an already complex story. However, if the Reviewer (and Editors) feel strongly we can give this a try, of course.

Manuscript change:
Page 16:
Finally, we assessed whether our novel PGSs improve prediction of AF on top of existing clinical risk prediction tools. Within the All of Us validation sets, focussing on incident AF, we found that both the ALLmeta and Mult-t scores remained strongly associated with AF when conditioned on the CHARGE-AF clinical model[30], across EUR, AMR and AFR ancestries (Figure S20 and S21; Table S19 and S20). We found that Hazard ratios for the Mult-t scores were more attenuated than for ALLmeta upon conditioning for CHARGE-AF; nevertheless, Mult-t scores still yielded higher Hazard ratios than

ALLmeta across the three ancestry groups, even after conditioning (Figure S20; Table S19). Similar patterns were observed for the HARMS2-AF clinical risk score[31] (Figure S22 and S23; Table S21 and S22).
In terms of C-index, we found that the addition of ALLmeta or Mult-t scores to CHARGE-AF improved prediction of incident AF (Figure S21; Table S20). For instance, the C-index among EUR participants increased from 0.80 (95%CI [0.78; 0.82]) to 0.82 ([0.80; 0.84]) when adding Mult-t-EUR to the model, a significant difference by Noether's test (one-sided P=7.7x10-34; Supplementary Table S23). For AFR and AMR, significant (one-sided P=8.2x10-5) and suggestive (one-sided P=0.09) improvements were observed upon adding Mult-t-AFR and Mult-t-AMR, respectively. The Mult-t scores also improved over ALLmeta PGS in models alongside CHARGE-AF (one-sided P<0.05 in EUR and AFR). Similar patterns were observed for the HARMS2-AF clinical score (Supplementary Table S23). These findings indicate that our novel PGSs represent independent risk indicators that improve discrimination of AF alongside clinical risk models. Future work should now assess whether clinical model-informed reweighting of PGS components may improve prediction further.

Pages 25-26:
Finally, we assessed whether our novel PGS would improve AF prediction on top of existing clinical primary risk prediction models. To this end, we assessed EUR, AMR and AFR ancestry groups in the All of Us dataset, assessing the utility of our Mult-t scores (mult-t-EUR, mult-t-AMR, mult-t-AFR) alongside both the CHARGE-AF and HARMS2-AF clinical scores[30,31], using Cox time-to-event regression models, testing for incident AF events (events after enrollment to All of Us). Models including the PGS as the sole predictor were adjusted for age, sex, and the first 20 principal components (PCs). Models including a clinical risk score alone, as well as models jointly including a clinical risk score and a PGS (residualized for the first 20 PCs), were adjusted for the first 10 PCs. The phenotypic components used to derive the CHARGE-AF and HARMS2-AF clinical risk scores are described in detail in Table S27. In particular, we assessed (i) the change in hazard ratios for PGS upon adjusting for either clinical risk model, and (ii) the change in model C-statistic upon addition of PGS to either clinical risk model.

3.8) Although this study focuses on AF PRS, aside from Fig. 3 there is little justification for emphasizing AF specifically. Could this framework also enhance performance for other traits?

Author response:
Yes, the framework we present can be applied to other genetically influenced traits, particularly those with well-powered GWAS and correlated phenotypes. Indeed, we have since successfully applied the pipeline to other traits/settings within our extended group. As an example, we below show results for prediction of Brugada syndrome from a small Ajmaline cohort (**Figure R6**), where the same multi-PGS pipeline significantly improves prediction.

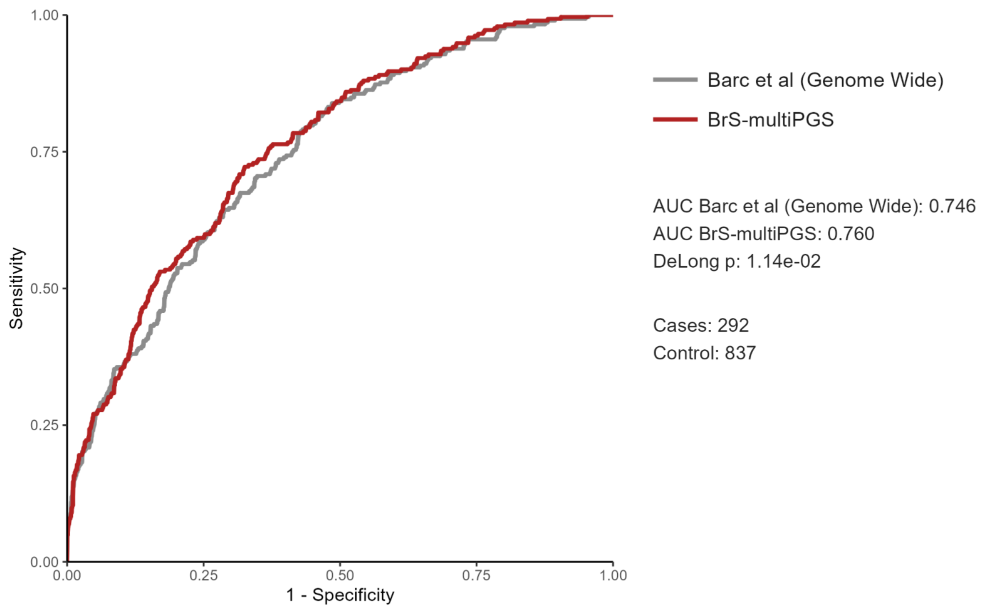

Figure R6 | Prediction of Brugada syndrome in an ajmaline testing cohort using multi-trait PGS. The left panel is a plot of an AUROC, showing 1-specificity on the x-axis and sensitivity on the y-axis. The gray line represents prediction using a single-trait PGS built from a published Brugada syndrome GWAS using SBayesRC, while the red line indicates prediction using a multi-trait PGS trained in a separate tuning set.

3.9) Related to the above point, aside from the improved stratification of high-risk individuals (Fig. 6), were there any findings that could be directly useful for AF clinical management?

Author response:
The main goal of our study was to improve global PGS performance and equitability, and not to identify concrete clinical use-cases for PGS. That being said, we agree with the Reviewer that this is an important point to consider, and therefore we have added two important analyses to our manuscript.

First, as mentioned above, we have added investigations into the joint predictive performance of PGS and clinical risk factors (**Figure R1-R2**). These results indicate that combining PGS with clinical risk models may improve primary prediction of AF, especially in Europeans, attaining C-statistics of around 0.82 (versus 0.80 in clinical-only models). Second, we have added data from the LOOP study, a clinical trial assessing implantable loop recorder screening in older participants without AF but at elevated cardiovascular risk. We found that ILR screening was associated with lower rates of stroke or systemic embolism in participants with higher Mult-t-EUR PGS (HR = 0.66, P=0.048), with no benefit in participants with lower Mult-t-EUR PGS (HR = 1.00, P=0.98). Data are presented in **Figure R7** (new **Figure S15**). Together, these results suggest that PGS can be used for pre-screening to identify individuals where targeted AF detection has benefit.

Manuscript change:
Page 11:

We also assessed the Mult-t-EUR score in the LOOP study, a Danish clinical trial assessing implantableloop recorder (ILR) screening in older participants without AF but at elevated cardiovascular risk[28]. In this high-risk population, the Mult-t-EUR score strongly outperformed the Roselli score (OR/SD = 1.54; AUROC = 0.607; Figure 5c; Table S15). Furthermore, recapitulating recent findings[29], we found that ILR screening was associated with lower rates of stroke or systemic embolism in participants with higher Mult-t-EUR PGS (HR = 0.66, P=0.048), with no benefit in participants with lower Mult-t-EUR PGS (HR = 1.00, P=0.99) (Figure S15).

Page 20:

For AF, a care pathway can be envisioned where individuals with a high PGS are prioritized for aggressive risk factor management and/or earlier routine AF screening[66], potentially to prevent strokes[29]. Still, further research in real-world settings is needed to assess the clinical utility, implementation, and ethical considerations of AF PGSs across diverse populations. At the same time, the field will need to firmly establish those clinical use-cases where PGS implementation stands to change standard care[1].

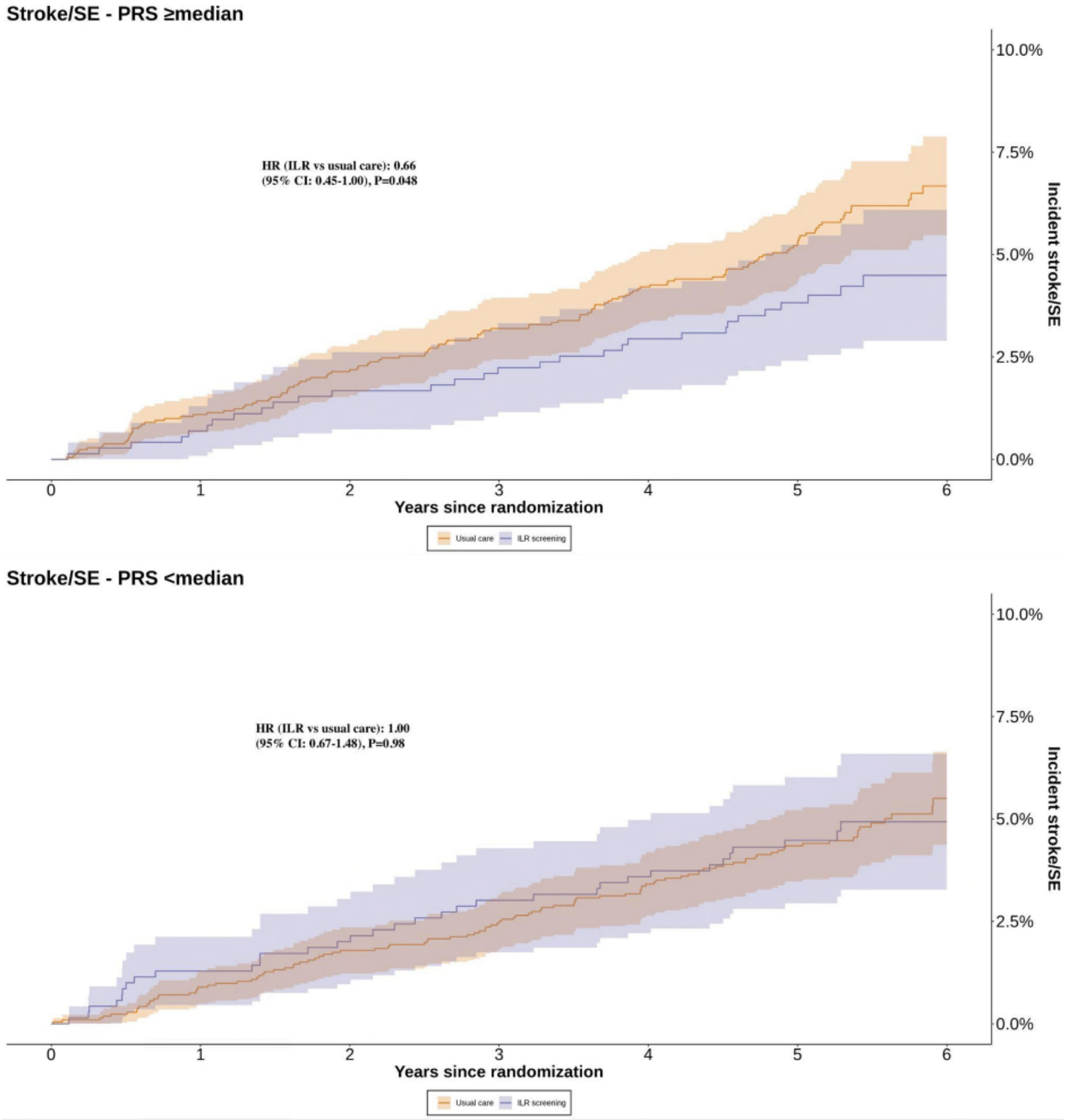

Figure R7 | Incident risk of stroke or systemic embolism after randomization to either implantable loop recorder (ILR) screening or usual care, in the LOOP study, stratified by Mult-t-EUR PGS. PRS>=median indicates the group of participants with Mult-t-EUR score larger than or equal to the median, while PRS<median indicates the group of participants with Mult-t-EUR lower than the median. In both panels, x-axis shows years since randomization and y-axis shows incident rates of stroke or systemic embolism (SE). Purple line (with 95% confidence interval) indicates participants randomized to ILR screening, while the orange line (with 95% confidence interval) indicates the participants randomized to usual care.

3.10) To demonstrate the generalizability of the findings, it would be valuable to validate the results in an additional external cohort, at least within the EUR population.

Author response:

We agree with the Reviewer. As mentioned above, we have added several additional cohorts, including two EUR cohorts (**Figure R5**).

3.11) Remarks on code availability: It contains concise scripts for running SBayesRC and METAL. It would be helpful if you could summarize the best pipeline for creating PGS (Polygenic Scores) as described in the actual paper.

Author response:
We agree that a concise overview of the PGS construction pipeline improves clarity and reproducibility. We have therefore added a complete walkthrough of the multi-trait PGS workflow to our GitHub repository, summarizing all major steps used in this study: https://github.com/poeyahay/AF_MultiTrait_PGS

Reviewer #4:

Haydarlou et al aimed to improve accuracy of AF risk prediction across ancestry groups. They applied the SBayesRC model, which incorporates functional annotation data, to the results from a new AF GWAS meta-analysis using data from the AFGen consortium (180 k cases) and the Million Veterans Program (88 k cases) and showed some improvement in AF prediction compared to previous polygenic risk scores (PRS)(e.g. Roselli et al). The model incorporates functional annotation data, and was tuned by ancestry, using the AL of Us cohort. To further improve the polygenic risk score, the authors created a multi-ancestry polygenetic risk score and a multi-trait score. The multi-ancestry scores did not improve risk prediction notably, but the multi-trait did - particularly in the Asian populatoin. Risk prediction remains most accurate in the European population. The methods used are sound, well-described and seems reprodicible, and the manuscript is very well written, with nice figures.

Author response:
We thank the Reviewer for their insightful review of our work, and for their helpful comments that have certainly helped improve the manuscript.

4.1) Adding other traits than AF in the prediction model is a novel and very interesting approach, which does improve risk prediction to some extent. However, when looking at the AUC, the improvements in prediction accuracy are only very modest, except perhaps for the simplified multi-t model applied to the Asian ancestry group. As seen in figure 2, the mult-t model increases the AUC by ~0.02-0.04 compared to the previous PRS from Roselli across ancestries.

Author response:
The Reviewer correctly mentions that the improvements in AUC values are modest. However, even incremental gains in AUC values can be meaningful—as evidenced by substantially improved case stratification at the extremes of our Mult-t-EUR score (**Figure 7**). Indeed, we recapitulate similar results in East-Asian ancestry using our new Mult-t-EAS PGS: despite small improvements in AUC, prediction in the tails is improved noticeably, as shown in **Figures R8-9** (new **Figures S18-19**).

Similarly, while AUC values tend to improve slowly over baselines, other metrics for prediction were indeed strongly improved by our approach, especially in non-Europeans. To this end, we have added new **Figure 3** (below as **Figure R10**) to better showcase the prediction improvements in the various ancestry groups.

Manuscript change:
Page 9:
Nevertheless, we found that our multi-trait approach provided relatively larger improvements in ancestries with worse baseline prediction (Figure 3; Table S8). For instance, when comparing our Mult-t scores to the ALLmeta score, relative improvements were strongest for SAS (beta improvement = 36.7%; AUC improvement = 26.6%; liability-scale R^2 improvement = 71.4%), AFR (19.7%; 10.6%;

55.6%) and AMR (12.3%; 4.4%; 34.4%), with only minor improvements in EUR (6.4%; 2.8%; 12.2%) and EAS (2.9%; 1.3%; 5.3%).

Page 14:

Given the strong prediction of our Mult-t-EAS score in BioBank Japan, we repeated similar analyses for Mult-t-EAS in the BioBank Japan validation set. Here, stratification at the extremes was even more pronounced, finding 22.6%, 11.3%, and 6.6% of individuals with at least 3-fold, 4-fold, and 5-fold elevated risk compared to the median (which improved over Roselli; Figure S18); we identified 9.14% at <⅓-fold risk (substantially improving over Roselli; Figure S19). These results indicate enhanced risk stratification for Mult-t-EUR and Mult-t-EAS at both ends of the distribution, as the scores more effectively shift high-risk individuals toward the upper tail and low-risk individuals toward the lower tail, while reducing their presence in the middle quintile.

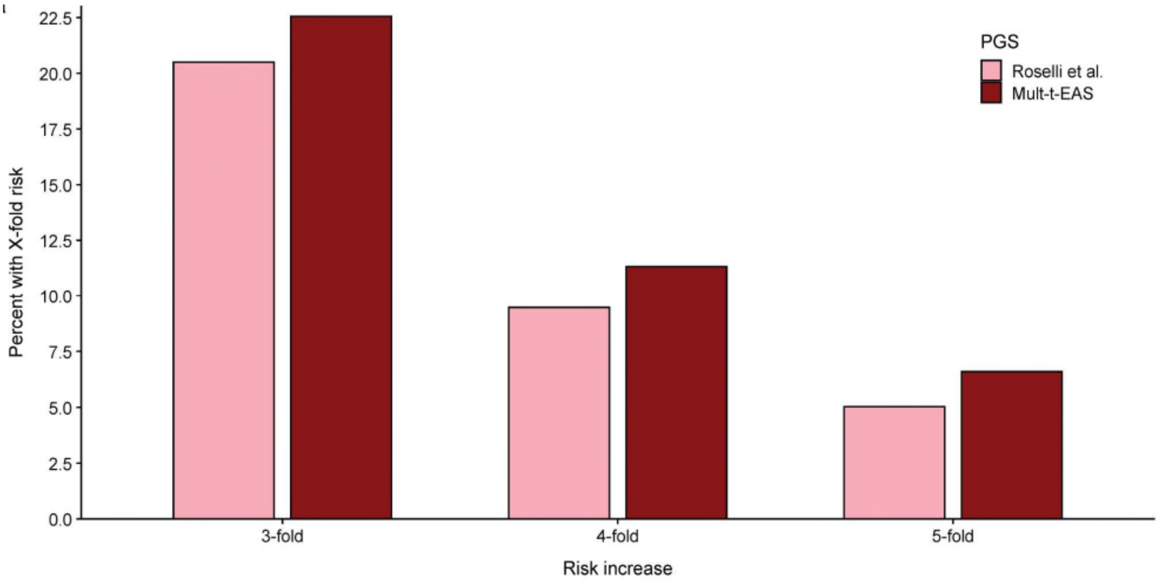

Figure R8 | Proportion of individuals with 3-, 4-, or 5-fold increased AF risk compared to the middle quintile within BBJ. All panels show the percentage of East Asian ancestry participants in the 70% validation dataset of Biobank Japan 2nd cohort (3,944 cases / 41,460 controls) with a 3-, 4-, or 5-fold higher risk of AF relative to the middle PGS quintile. **a,** Bar plots display the proportions in each risk group for the Roselli et al. PGS (pink) and the Mult-t-EAS PGS (dark red). **b,c,** Density plots of the Roselli et al.

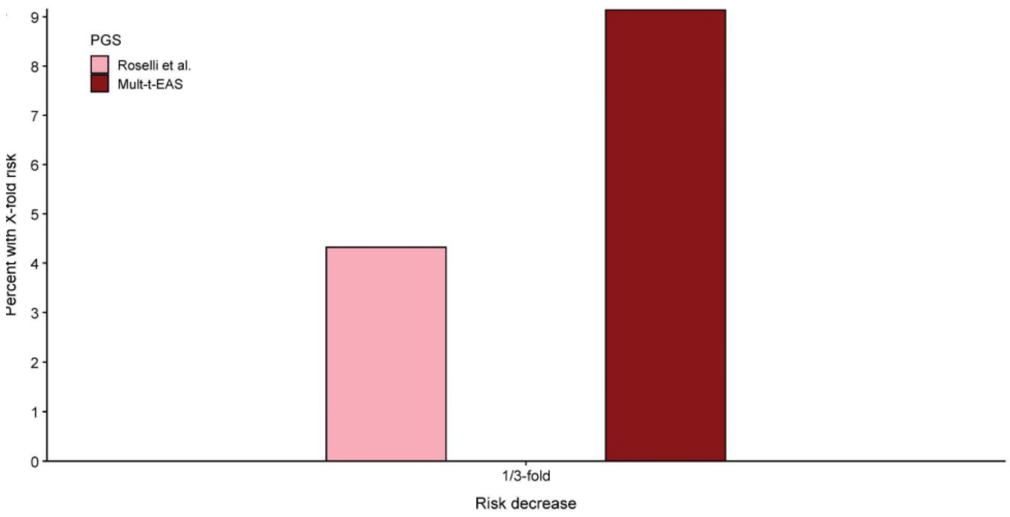

Figure R9 | Proportion of individuals with 1/3-fold decreased AF risk compared to the middle quintile within BBJ. All panels show the percentage of East Asian ancestry participants in the 70% validation dataset of Biobank Japan 2nd cohort (3,944 cases / 41,460 controls) with a 1/3-fold lower risk of AF relative to the middle PGS quintile. **a,** Bar plots display the proportion with a 1/3-fold lower risk for the Roselli et al. PGS (pink) and the Mult-t-EAS PGS (dark red).

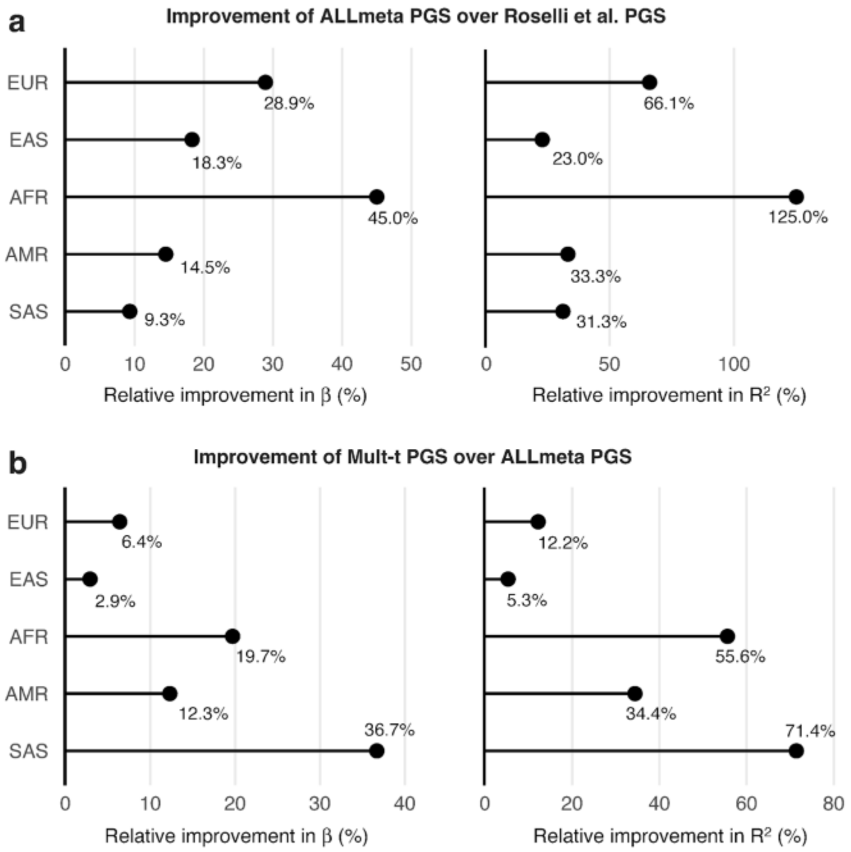

Figure R10 | Relative improvements of novel PGSs per ancestry group in *All of Us*. All panels represent lollipop charts, with relative improvements (%) in prediction on the x-axes and different ancestry groups on the y-axes. In both parts, the left plot shows prediction as measured by logistic regression beta coefficients, while the right plots shows prediction as measured by liability-scale variance explained. Part **a** shows the relative prediction improvements of the ALLmeta SBayesRC score over the Roselli et al. PGS, while part **b** shows relative prediction improvements of our ancestry-optimized Mult-t PGSs over the ALLmeta PGS. All estimates are based on logistic regression models adjusted for the first 20 PCs, age, and sex, using case/control counts

from the 70% validation dataset of All of Us (**Table S1**). Due to limited sample size, the full 100% validation set was used for SAS and EAS ancestry. Data are also provided in **Table S8**.

4.2) Moreover, an AUC of 0.6 is not impressive when it comes to risk prediction in general, so it would also be interesting to see how the model performs adding clinical and other known risk factors.

We think this is a great suggestion by the Reviewer: Evaluating PGS in combination with clinical risk factors is an important direction for potential clinical utility. We examined this by adding either the CHARGE-AF or HARMS-AF clinical risk scores to our PGS models (**Figure R1-R2**). As detailed in our response to Reviewer #1 (comment 1.1), incorporating clinical risk scores improved model performance, and the combined models demonstrated significantly higher predictive accuracy than the PGS or clinical scores alone in most models. For instance, among EUR, the combined model achieved a C-index of 0.82 (as compared to 0.80 for a clinical-only model; P=8x10^{-34}).

Manuscript change:
Page 16:
Finally, we assessed whether our novel PGSs improve prediction of AF on top of existing clinical risk prediction tools. Within the All of Us validation sets, focussing on incident AF, we found that both the ALLmeta and Mult-t scores remained strongly associated with AF when conditioned on the CHARGE-AF clinical model[30], across EUR, AMR and AFR ancestries (Figure S20 and S21; Table S19 and S20). We found that Hazard ratios for the Mult-t scores were more attenuated than for ALLmeta upon conditioning for CHARGE-AF; nevertheless, Mult-t scores still yielded higher Hazard ratios than ALLmeta across the three ancestry groups, even after conditioning (Figure S20; Table S19). Similar patterns were observed for the HARMS2-AF clinical risk score[31] (Figure S22 and S23; Table S21 and S22).
In terms of C-index, we found that the addition of ALLmeta or Mult-t scores to CHARGE-AF improved prediction of incident AF (Figure S21; Table S20). For instance, the C-index among EUR participants increased from 0.80 (95%CI [0.78; 0.82]) to 0.82 ([0.80; 0.84]) when adding Mult-t-EUR to the model, a significant difference by Noether's test (one-sided P=7.7x10^{-34}; Supplementary Table S23). For AFR and AMR, significant (one-sided P=8.2x10-5) and suggestive (one-sided P=0.09) improvements were observed upon adding Mult-t-AFR and Mult-t-AMR, respectively. The Mult-t scores also improved over ALLmeta PGS in models alongside CHARGE-AF (one-sided P<0.05 in EUR and AFR). Similar patterns were observed for the HARMS2-AF clinical score (Supplementary Table S23). These findings indicate that our novel PGSs represent independent risk indicators that improve discrimination of AF alongside clinical risk models. Future work should now assess whether clinical model-informed reweighting of PGS components may improve prediction further.

Pages 25-26:
Finally, we assessed whether our novel PGS would improve AF prediction on top of existing clinical primary risk prediction models. To this end, we assessed EUR, AMR and AFR ancestry groups in the All of Us dataset, assessing the utility of our Mult-t scores (mult-t-EUR, mult-t-AMR, mult-t-AFR) alongside both the CHARGE-AF and HARMS2-AF clinical scores[30,31], using Cox time-to-event regression models, testing for incident AF events (events after enrollment to All of Us). Models including the PGS as the sole predictor were adjusted for age, sex, and the first 20 principal components (PCs). Models including a clinical risk score alone, as well as models jointly including a clinical risk score and a PGS (residualized for the first 20 PCs), were adjusted for the first 10 PCs. The phenotypic components used to derive the CHARGE-AF and HARMS2-AF clinical risk scores are described in detail in Table

S27. In particular, we assessed (i) the change in hazard ratios for PGS upon adjusting for either clinical risk model, and (ii) the change in model C-statistic upon addition of PGS to either clinical risk model.

4.3) The results from the novel GWAS meta-analysis of data from the AFGen cohort and the MVP cohort are not described at all in the text or supplement and there are no tables or figures. The results would be very interesting to the reader - particularly any novel loci for AF - and should be added.

Thank you for the suggestion. First, we have added Manhattan plots and QQ-plots to visualize the GWAS results, as shown in **Figure R11-12** (new **Figure S26-27**). Second, we have applied (i) a systematic locus definition pipeline and (ii) a machine-learning gene-prioritization pipeline - identify significant loci and potential effector genes (overview of both presented in new **Table S28**). Notably, the GWAS identified 486 significant loci, with machine-learning prioritized genes available for ~85% of these. Compared to a recent GWAS of comparable size[6], we identified ~10% fewer loci, although analyses also suggest that our GWAS is affected 2- to 3-fold less by inflation/bias (substantial discussion added to the **Supplementary Note**).

Supplementary Note change:
Page 29-31:

To comprehensively characterise genome-wide significant signals and prioritise candidate genes, we applied two complementary approaches for locus definition and gene assignment:

- *a distance-based nearest-gene approach using GWAS summary statistics, and*
- *a fine-mapping– and machine-learning-informed gene prioritisation approach.*

For the distance-based approach, genome-wide significant loci were defined from AFib GWAS summary statistics using the get_loci() function from the gwasRtools R package. Variants were first filtered to retain those with valid chromosome, position, P-value, and allele frequency information. Genome-wide significant variants ($P < 5 \times 10^{-8}$) were identified, and for each variant a ±500 kb window (1 Mb total) was constructed. Overlapping windows were merged to define independent GWAS loci. Within each locus, the lead SNP was defined as the variant with the smallest p-value.
For each lead SNP, the nearest gene was annotated based on physical distance to the gene body using get_nearest_gene() (GRCh37 build). This approach provides a simple and interpretable baseline gene assignment based solely on genomic proximity to the association signal.
For machine-learning-based gene prioritization, in parallel, we applied FLAMES, a fine-mapping–based gene prioritisation framework that integrates statistical fine-mapping results with gene-level aggregation[25]. FLAMES leverages posterior inclusion probabilities derived from SuSiE fine-mapping to quantify the contribution of variants within a locus and assigns gene-level scores by aggregating evidence across variants mapped to each gene. This approach prioritises genes based on the overall fine-mapping signal rather than reliance on a single lead variant.
Fine-mapping and FLAMES analyses were performed using linkage disequilibrium estimated from the UK Biobank European (EUR) reference panel, providing a consistent LD structure for probabilistic inference across loci.
To assess concordance between GWAS-defined loci and FLAMES fine-mapping results, we implemented a positional overlap strategy. For each GWAS locus defined by get_loci(), a ±500 kb window was constructed around the lead SNP. FLAMES fine-mapped variants from credible sets with FLAMES score > 0 and P < 5e-8 were then considered overlapping with lead get_loci() variants if they fell within this window of 500 kb bp on the same chromosome. For loci with multiple overlapping FLAMES variants, a single representative hit was selected based on the highest SuSiE posterior inclusion probability, with ties further prioritised by higher FLAMES score and lower GWAS P-value.

Loci without any FLAMES variants in the ±500 kb window were retained and marked as non-overlapping.
This dual strategy allowed us to contrast simple proximity-based gene assignment with a more sophisticated fine-mapping–informed approach, and to identify loci where gene prioritisation is sensitive to LD reference structure and fine-mapping resolution. The summary of this approach is provided in Table S28.
The distance-based approach led to the discovery of 486 distinct loci in our novel AF GWAS meta-analysis (Figure S26 and S27). FLAMES identified 1029 credible sets that (i) contain at least one genome-wide significant variant and (ii) that could be mapped to a machine-learning prioritized gene (FLAMES_score > 0). Of the 486 distinct loci based on distance, 416 loci (85.6%) overlapped with such a credible-set with a FLAMES-prioritized gene (Tables S28). Of note: (i) several credible sets may map to a single locus - explaining why 1029 credible sets become 416 loci - and (ii) some loci cannot be reliably fine-mapped (due to mismatches between LD reference and our multi-ancestry GWAS data) and therefore did not undergo FLAMES prioritization.
Compared to a recent AF GWAS with comparable case numbers[26], we identified fewer loci: Yuan et al. analyzed 252,438 AF cases and identified 525 significant loci. However, we note that Yuan et al. reported a substantial LD score regression intercept of 1.34 (with an attenuation ratio of 15%). In contrast, using LD score regression and using a European LD reference, we estimate an LD score intercept of our GWAS of 1.11 (SE=0.018; attenuation ratio 7.3%). Therefore, our GWAS data seem to contain 2-3-fold less bias, potentially explaining some difference in locus discovery and hinting at better locus specificity in our GWAS as compared to Yuan et al.

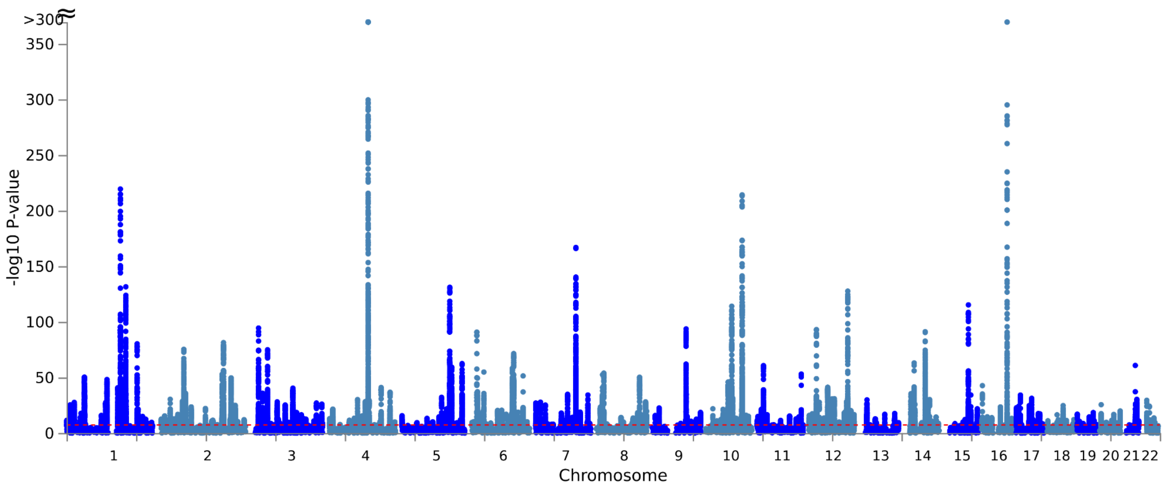

Figure R11 | Manhattan plot for novel AF GWAS meta-analysis discovering 486 loci. The figure shows a Manhattan plot with each dot representing a single variant association test, while the y-axis represents the -log10 of the association P-value for the given variant and the x-axis represents the genomic position of the variant. The dotted line shows the genome-wide significance (*P*<5x10^{-8}) threshold. Variants in this plot are filtered to MAF>1%.

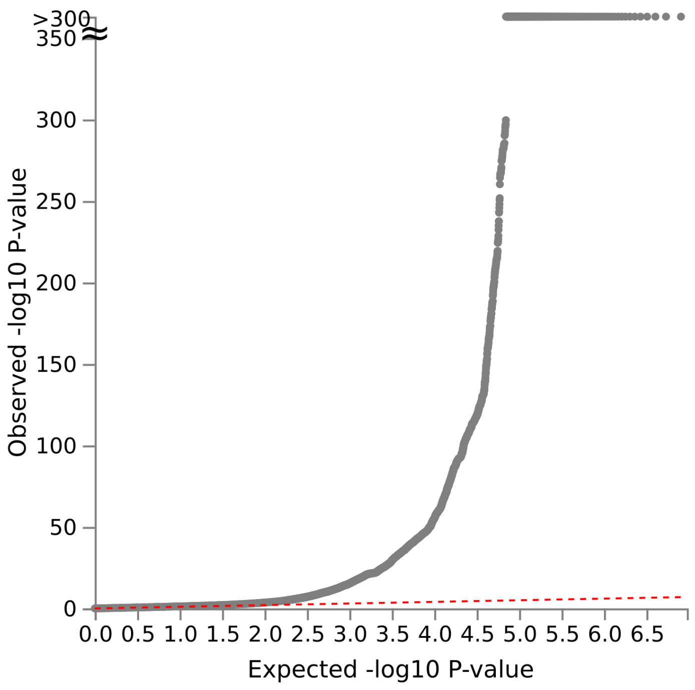

Figure R12 | Quantile-quantile plot for novel AF GWAS meta-analysis. The figure shows a quantile-quantile plot with each dot representing a single variant association test, while the y-axis represents the observed -log10 of the association P-value for the given variant and the x-axis expected -log10 of the association P-value under the null hypothesis of no association. The dotted red line shows x=y line.

4.4) Many other efforts have been made to develop PRS for AF. The authors state in the introduction that PRSs face several major barriers that limit their clinical utility, for instance the lack of clinical use-cases and lack of reproducibility to diverse ancestry groups. The authors should be commended for aiming to build a PRS that performs better across ancestries, but it would be of importance to further assess how the mult-t model would perform in a clinical-use case, and I think that the manuscript would improve if that was added.

Author response:
As discussed also in our response to Reviewer #3 (comment 3.9), the main goal of our study was to improve global PGS performance and equitability, and not to identify concrete clinical use-cases for PGS. That being said, we agree that clinical use-cases are important to consider, and therefore we have added two important analyses to our manuscript to address this point.

First, as mentioned above, we have added investigations into the joint predictive performance of PGS and clinical risk factors (**Figure R1-R2**). These results indicate that combining PGS with clinical risk models may improve primary prediction of AF. Second, we have added data from the LOOP study, a clinical trial assessing implantableloop recorder screening in older participants without AF but at elevated cardiovascular risk. We found that ILR screening was associated with lower rates of stroke or systemic embolism

in participants with higher Mult-t-EUR PGS (HR = 0.66, P=0.048), with no benefit in participants with lower Mult-t-EUR PGS (HR = 1.00, P=0.98). Data are presented in **Figure R7** (new **Figure S15**). Together, these results suggest that PGS can be used for pre-screening to identify individuals where targeted AF detection has benefit.

Manuscript change:

Pages 11:

We also assessed the Mult-t-EUR score in the LOOP study, a Danish clinical trial assessing implantableloop recorder (ILR) screening in older participants without AF but at elevated cardiovascular risk[28]. In this high-risk population, the Mult-t-EUR score strongly outperformed the Roselli score (OR/SD = 1.54; AUROC = 0.607; Figure 5c; Table S15). Furthermore, recapitulating recent findings29, we found that ILR screening was associated with lower rates of stroke or systemic embolism in participants with higher Mult-t-EUR PGS (HR = 0.66, P=0.048), with no benefit in participants with lower Mult-t-EUR PGS (HR = 1.00, P=0.98) (Figure S15).

Pages 20:

For AF, a care pathway can be envisioned where individuals with a high PGS are prioritized for aggressive risk factor management and/or earlier routine AF screening[66], potentially to prevent strokes[29]. Still, further research in real-world settings is needed to assess the clinical utility, implementation, and ethical considerations of AF PGSs across diverse populations. At the same time, the field will need to firmly establish those clinical use-cases where PGS implementation stands to change standard care[1].

4.5) I would suggest to use the abbreviation PRS in stead of PGS to avoid confusion, since the latter is more widespread.

Author response:

We appreciate the suggestion. We have decided to retain the abbreviation PGS throughout the manuscript, given that PGS is a term that can be used more generally (e.g., also for non-binary/non-disease traits). However, if the Reviewer and Editors feel strongly, we can change this.

References

1. Polygenic Risk Test. https://www.massgeneralbrigham.org/en/research-and-innovation/centers-and-programs/personalized-medicine/molecular-medicine/tests/polygenic-risk#accordion-a808d6fe3e-item-5de45a7c54.
2. Andersen, J. H., Andreasen, L. & Olesen, M. S. Atrial fibrillation-a complex polygenetic disease. *Eur J Hum Genet* **29**, 1051–1060 (2021).
3. Mosley, J. D. *et al.* Investigating the Genetic Architecture of the PR Interval Using Clinical Phenotypes. *Circ Cardiovasc Genet* **10**, (2017).
4. Segan, L. *et al.* Combining polygenic and clinical risk scores in atrial fibrillation risk prediction: Implications for population screening. *Heart rhythm* (2025) doi:10.1016/j.hrthm.2025.04.032.
5. Marston, N. A. *et al.* A polygenic risk score predicts atrial fibrillation in cardiovascular disease. *European heart journal* **44**, (2023).
6. Yuan, S. *et al.* Cross-population GWAS and proteomics improve risk prediction and reveal mechanisms in atrial fibrillation. *Nat Commun* **16**, 6426 (2025).

Reviewer #1:

1.1) All of my comments have been addressed thoroughly, and I think all of Reviewer 3's comment have been addressed, too.

Author response:
We would like to thank the Reviewer for the positive evaluation and for recognizing that all comments have been thoroughly addressed.

Reviewer #2:

2.1) I co-reviewed this manuscript with one of the reviewers who provided the listed reports. This is part of the Nature Communications initiative to facilitate training in peer review and to provide appropriate recognition for Early Career Researchers who co-review manuscripts.

Author response:
We thank the Reviewer for their contribution to the peer review process and appreciate the involvement in the Nature Communications initiative to support training and recognition of Early Career Researchers in peer review.

Reviewer #4:

4.1) The authors have addressed my comments in a very good way, and the manuscript has improved substantively after the major revisions performed.
I would still have liked to see a more tangible clinical-use case beyond the replication of previous results from Vad et al in the Loop study. But I also agree that it may be beyond the scope of the current manuscript.

Author response:
We very much appreciate the Reviewer's acknowledgment that the manuscript has improved substantively following the revisions. We agree that demonstrating a more tangible clinical use case for our polygenic score would be very interesting.While this was beyond the scope of the current manuscript, it is an important direction that we will consider in future work.

I only have a few minor comments:

In my previous comment 4.2, I suggested that the authors should evaluate the PGS performance compared with clinical risk models, which the authors have done. In the manuscript change on page 16, the authors state that "In terms of C-index, we found that the addition of ALLmeta or Mult-t scores to CHARGE-AF improved prediction of incident AF (Figure S21; Table S20). For instance, the C-index among EUR participants increased from 0.80 (95%CI [0.78; 0.82]) to 0.82 ([0.80; 0.84]) when adding Mult-t-EUR to the model, a significant difference by Noether's test (one-sided P=7.7x10-34; Supplementary Table

S23). For AFR and AMR, significant (one-sided P=8.2x10-5) and suggestive (one-sided P=0.09) improvements were observed upon adding Mult-t-AFR and Mult-t-AMR, respectively."

4.2) I would omit "suggestive" significance here, with a p-value of 0.09, which I would say is not significant.

Author response:
We thank the reviewer for sharply pointing out that the improvement observed in the AMR cohort (P = 0.09) should not be described as "suggestive significance," and we have revised the manuscript accordingly.

Manuscript change:
Page 15:
For AMR and AFR, non-significant (one-sided P=0.09) and significant (one-sided P=8.2x10-5) improvements were observed upon adding Mult-t-AMR and Mult-t-AFR, respectively.

4.3) The differences in across ancestry groups in table S23 are interesting. The authors discuss the significance of improving genetic prediction of AF in underrepresented populations in the discussion section, but do not reflect on the fact that the new mult-t PGS adds relatively little to AF risk prediction beyond the clinical risk scores in the AMR and AFR populations, compared with EUR, as shown in figure S21 and Table S23. Can the authors speculate on the causes of the differences? If one takes into account the cost of genotyping, it may look like using a clinical risk score may be preferable or just as good as a PGS in certain population, especially in a cost-benefit view.

Author response:

Regarding the smaller incremental improvement in AF risk prediction beyond clinical risk scores in AMR and AFR populations compared with EUR participants (Figure S21 and S23; Table S23), we agree that this observation warrants further consideration. Several factors may contribute to these differences.

First, as illustrated in Figure 4, the Mult-t-EUR score derives a relatively large contribution from the AF-specific PGS, whereas the Mult-t-AMR and Mult-t-AFR scores rely more heavily on PGSs for AF-correlated traits. Many of these correlated traits (e.g., height, body mass index, heart failure, and systolic blood pressure) overlap with components of the clinical risk models, CHARGE-AF and HARMS-AF. Consequently, in AMR and AFR populations the multi-trait PGS may capture more information that is already partially represented by the clinical predictors, resulting in a smaller incremental improvement in predictive performance beyond the clinical model.

Second, the number of incident AF cases was substantially lower in the AMR and AFR groups (509 and 794 cases, respectively) compared with EUR participants (3,006 cases).

The smaller number of events likely reduces the precision of the C-index estimates and limits statistical power to detect incremental improvements in predictive performance.

Finally, the relative contribution of the AF-specific PGS component is substantially influenced by the current imbalance in GWAS sample sizes across ancestries. Larger AF GWAS datasets in non-European populations should improve the performance of ancestry-specific AF PGS components, which in turn could increase the incremental value of multi-trait PGS beyond clinical risk scores in these populations.

We also agree with the reviewer that, in certain contexts—such as in older individuals with multiple established clinical risk factors—a clinical risk score alone may provide sufficient risk stratification and may be more cost-effective than genotyping. However, PGS may offer particular value for identifying elevated lifetime risk earlier in life, before the development of clinical risk factors. Moreover, although our multi-trait AF PGS captures approximately half of the common variant heritability, predictive performance is expected to improve as GWAS sample sizes increase and polygenic score methods continue to evolve. Our study contributes to this progress by developing multi-trait PGS that bring genetic risk prediction closer to capturing the heritable component of AF risk.

4.4) Would it be possible for the authors to incorporate p-values for comparison between models in their figures showing C-statistics? It would be helpful to see it directly in the figure instead of digging though supplemental material. E.g. in figure 2 – adding an asterisk denoting a significant improvement in predictive accuracy for the mult-t-EUR vs the Roselli PGS, etc.

We thank the reviewer for this helpful suggestion. We have revised figures in question to include an additional column reporting the P-values for comparisons with the CHARGE-AF/HARMS-AF models using a one-sided Noether test (Figures R1 and R2, formerly Figures S21 and S23), and for comparisons with the Roselli et al. PGS using a one-sided DeLong test (Figure R3, formerly Figure 2). This allows readers to directly assess whether differences in C-index or AUROC between models are statistically significant without needing to consult the supplementary tables.

Manuscript change:
Page 8:
P-values were calculated using two-sided Wald tests, except for comparisons with the Roselli et al. PGS, which used a one-sided DeLong test.

Supplementary Information change:
Page 44:
The right panel reports the C-index values numerically and the P-values for comparisons with the CHARGE-AF model using a one-sided Noether test.
Page 45:
The right panel reports the C-index values numerically and the P-values for comparisons with the HARMS2-AF model using a one-sided Noether test.

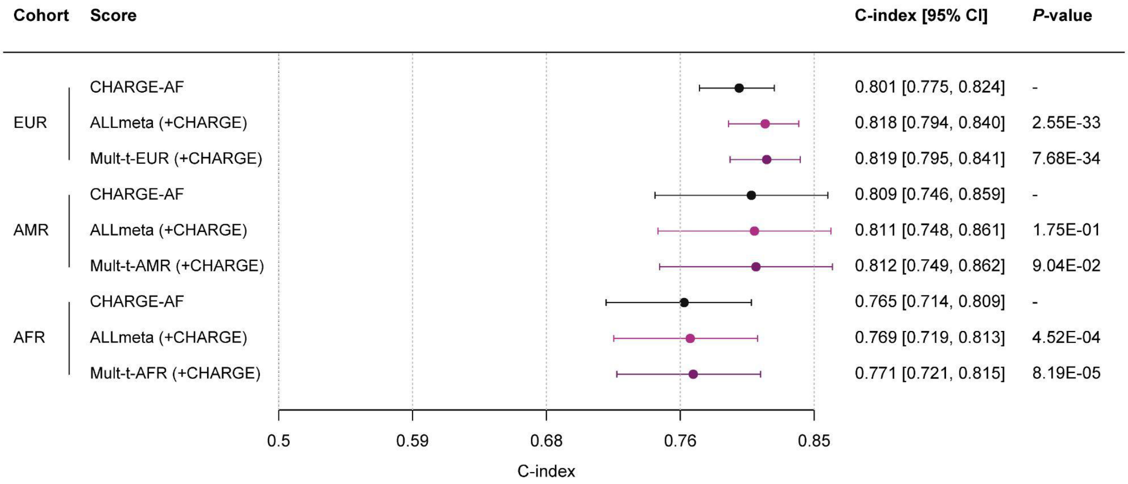

Figure R1 | Harrell's C-indices for incident AF prediction using CHARGE-AF and PGS + CHARGE-AF models. The left panel displays a forest plot of Harrell's C-index with 95% CIs on the x-axis. The y-axis lists ancestry-specific cohorts from All of Us (Supplementary Table 19): EUR (n=103,601 of which 2,749 incident cases), AMR (n=33,610 of which 449 incident cases), and AFR (n=39,697 of which 758 incident cases), each paired with its scores. The right panel reports the C-index values numerically and the *P*-values for comparisons with the CHARGE-AF model using a one-sided Noether test. The CHARGE-AF score is a clinical risk score for atrial fibrillation derived from multiple phenotypes Supplementary Table 27). Estimates are based on cox regression models using CHARGE-AF alone (adjusted for the first 10 PCs) or models including the ALLmeta or multi-trait PGSs (each residualized for the first 20 PCs) with CHARGE-AF included as a covariate, with the full model adjusted for the first 10 PCs. Source Data are provided (Supplementary Table 20). *P*-values for model comparisons are available (Supplementary Table 23).

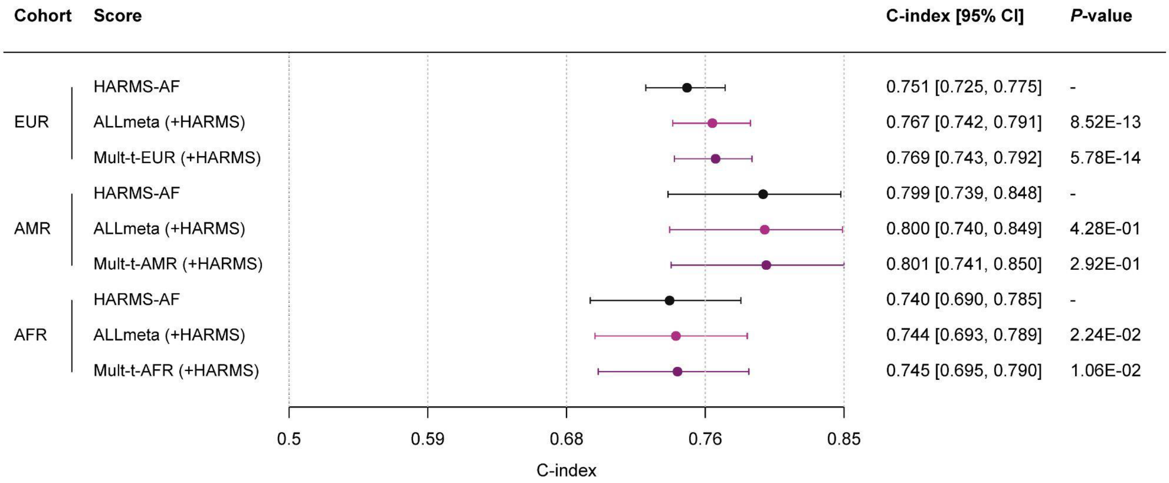

Figure R2 | Harrell's C-indices for incident AF prediction using HARMS2-AF and PGS + HARMS2-AF models. The left panel displays a forest plot of Harrell's C-index with 95% CIs on the x-axis. The y-axis lists ancestry-specific cohorts from All of Us (Supplementary Table 21): EUR (n=113,525 of which 3,006 incident cases), AMR (n=37,585 of which 509 incident cases), and AFR (n=40,984 of which 794 incident cases), each paired with its scores. The right panel reports the C-index values numerically and the *P*-values for comparisons with the HARMS2-AF model using a one-sided Noether test. The HARMS2-AF score is a clinical risk score for atrial fibrillation derived from multiple phenotypes (Supplementary Table 27). Estimates are based on cox regression models using HARMS2-AF alone (adjusted for the first 10 PCs) or models including the ALLmeta or multi-trait PGSs (each residualized for the first 20 PCs) with HARMS2-AF included as a covariate, with the full model adjusted for the first 10 PCs. Source Data are provided (Supplementary Table 22). *P*-values for model comparisons are available (Supplementary Table 23).

Cohort	PGS	OR [95% CI]	*P*-value	AUROC	AUPRC	Ngk-R^2	Liab-R^2	DeLong
EUR	Roselli et al.	1.59 [1.56, 1.62]	<1.00E-300	0.613	0.137	0.040	0.059	-
	ALLmeta	1.82 [1.78, 1.85]	<1.00E-300	0.642	0.155	0.065	0.098	4.55E-53
	Mult-t-EUR	1.89 [1.85, 1.93]	<1.00E-300	0.646	0.157	0.071	0.110	8.52E-59
AMR	Roselli et al.	1.34 [1.27, 1.41]	6.98E-28	0.578	0.050	0.012	0.024	-
	ALLmeta	1.39 [1.32, 1.47]	2.93E-37	0.591	0.053	0.017	0.032	4.23E-03
	Mult-t-AMR	1.45 [1.38, 1.53]	7.11E-45	0.595	0.054	0.021	0.043	5.09E-03
AFR	Roselli et al.	1.21 [1.15, 1.27]	1.43E-14	0.548	0.056	0.004	0.008	-
	ALLmeta	1.32 [1.25, 1.38]	3.76E-30	0.566	0.061	0.010	0.018	4.84E-04
	Mult-t-AFR	1.39 [1.32, 1.45]	6.42E-43	0.573	0.064	0.014	0.028	6.15E-05
SAS	Roselli et al.	1.28 [1.03, 1.59]	2.42E-02	0.583	0.030	0.008	0.014	-
	ALLmeta	1.31 [1.05, 1.63]	1.73E-02	0.579	0.030	0.009	0.020	5.78E-01
	Mult-t-SAS	1.45 [1.16, 1.81]	1.29E-03	0.600	0.033	0.016	0.038	2.28E-01
EAS	Roselli et al.	1.64 [1.42, 1.89]	1.90E-11	0.624	0.052	0.034	0.061	-
	ALLmeta	1.79 [1.55, 2.07]	2.67E-15	0.657	0.055	0.047	0.075	3.44E-03
	Mult-t-EAS	1.82 [1.58, 2.11]	4.55E-16	0.659	0.061	0.050	0.079	3.68E-03

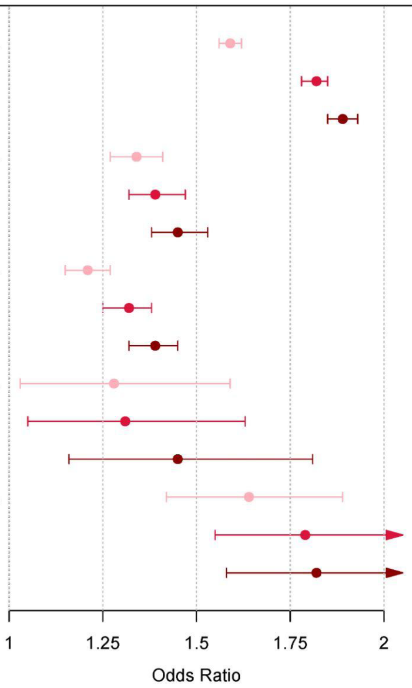

Figure R3 | Comparison of the Roselli et al., ALLmeta, and Mult-t PGSs for AF prediction in All of Us. The left panel displays a forest plot of the OR/SD increase in PGS for AF, with 95% CIs on the x-axis (Supplementary Table 7). The y-axis lists ancestry-specific cohorts from the All of Us validation dataset (Supplementary Table 1): EUR (n=124,367 of which 11,087 cases), AMR (n=40,219 of which 1,449 cases), AFR (n=44,692 of which 2,112 cases), SAS (n=3,806 of which 90 cases), and EAS (n=6,832 of which 198 cases), each paired with its PGSs. EUR, AMR, and AFR are based on the 70% validation subset, whereas the full 100% validation set was used for SAS and EAS due to limited sample size. The right panel summarizes key performance metrics. All estimates are based on logistic regression models adjusted for the first 20 PCs, age, and sex, except for AUROC and AUPRC values which are based on univariate models (to show prediction from PGS alone). *P*-values were calculated using two-sided Wald tests, except for comparisons with the Roselli et al. PGS, which used a one-sided DeLong test. Source Data are provided (Supplementary Table 7).
